# Synthesis and Biological Assessment of New Thiazoles for Mycobacterial Infections and Biofilm Disruption

**DOI:** 10.1021/acsomega.5c06421

**Published:** 2025-10-18

**Authors:** Laís Regina dos Santos Folquitto, Fallon dos Santos Siqueira, Tayná Roberta Nunes, Thiago Belarmino de Souza, Diogo Teixeira Carvalho, Rafael Pereira Machado, Antonio Carlos Doriguetto, Marli Matiko Anraku De Campos, Livia de Figueiredo Diniz, Marisi Gomes Soares, Daniela Aparecida Chagas de Paula, Danielle Ferreira Dias

**Affiliations:** a Instituto de Química, 74347 Universidade Federal de Alfenas, Alfenas, MG 37130-001, Brazil; b Departamento de Análises Clínicas e Toxicológicas, 28118 Universidade Federal de Santa Maria, Santa Maria, RS Santa Maria, Brazil; c Escola de Farmácia, 28115 Universidade Federal de Ouro Preto, Ouro Preto, MG 35400-000, Brazil; d Faculdade de Ciências Farmacêuticas, 74347 Universidade Federal de Alfenas, Alfenas, MG 37130-001, Brazil; e Instituto de Ciências Biomédicas, 74347 Universidade Federal de Alfenas, Alfenas, MG 37130-001, Brazil

## Abstract

Sixteen thiazoles, of which nine are unprecedented substances (**11**, **12**, **15**, **16**, **17**, **19**, **20**, **23**, and **24**), were obtained by a cyclocondensation reaction between a thioamide and an α-bromoketone, via Hantzsch synthesis. All thiazoles (**11**–**26**), along with four thiosemicarbazone derivatives (**7**–**10**) and their precursors (**1**–**6**), were evaluated for their activity against *Mycobacterium* species *Mycobacterium abscessus*, *Mycobacterium massiliense*, *Mycobacterium fortuitum*, and *Mycobacterium smegmatis*, as well as for their antibiofilm properties. Among them, compounds **7, 8**, **14**, **17**, **18**, **19**, **20**, and **21** showed promising results in minimum inhibitory concentration (MIC) assays, demonstrating bactericidal activity within 48 h. Moreover, all these compounds inhibited biofilm formation. Notably, the unprecedented thiazole **17**, along with **18** (MIC = 36 μmol L^–1^) and **21** (MIC = 65 μmol L^–1^), exhibited the lowest MIC values against all tested species, outperforming the reference drugs. Furthermore, these compounds showed a high degree of selectivity toward mycobacterial cells, as confirmed by cytotoxicity assays using peripheral blood mononuclear cells (PBMC) and Vero cells. These findings highlight the strong antimycobacterial potential of the new thiazole derivatives, warranting further investigation.

## Graphical abstract

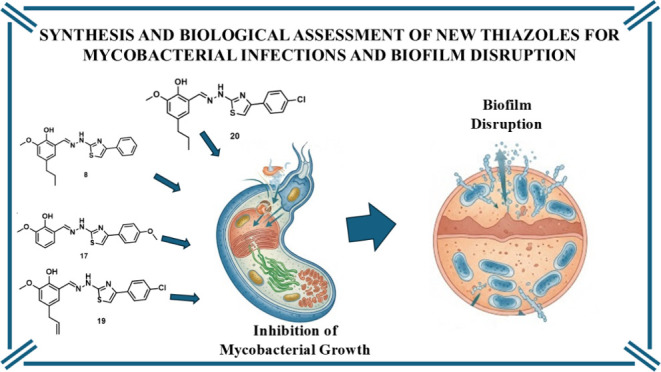

## 1 Introduction

Mycobacteria are significant pathogens responsible for infections in humans^1^ and represent a major concern, posing serious and frequent challenges to health systems.^2^ As their natural habitat is the environment, human exposure to mycobacteria is extensive, and they are considered opportunistic pathogens.^3^ In recent years, infections caused by nontuberculous mycobacteria (NTM) have been on the rise and are associated with a wide range of clinical syndromes in both immunocompromised and immunocompetent individuals.^1^

Postoperative infections caused by rapidly growing mycobacteria (RGM) have been widely reported. Species such as *Mycobacterium fortuitum*, *Mycobacterium abscessus*, *Mycobacterium chelonae*, and subspecies of the *M. abscessus* group—such as *abscessus sensu stricto*, *massiliense*, and *bolletii*—are increasingly identified as causes of skin, ocular, or subcutaneous infections.^3,4^ These infections can be acquired during a wide range of clinical procedures, including cardiothoracic, ophthalmologic, orthopedic, laparoscopic, and aesthetic surgeries.^4,5^

The ability to form biofilms, by adhering to each other and producing a resistant extracellular matrix, confers high resistance to these microorganisms, both against the antimicrobials used to treat infections and the disinfecting agents used in the sterilization of hospital materials. Their rigid, lipid-rich cell wall facilitates biofilm formation through hydrophobic interactions, making eradication particularly difficult.^5^ *M. abscessus* is recognized as the most virulent among them, capable of forming biofilms even faster than *M. fortuitum* and *M. chelonae*, which can form biofilms within 48 h.^1^

These characteristics of RGM make treatment even more difficult.^5^ The high resistance to commercially available antimicrobials—for example, rifampicin, isoniazid, and macrolides—as well as the challenges faced in treating the infections they cause, drive the ongoing search for new agents with antimycobacterial activity.^1,6,7^

Given the structural relevance of heterocycles—widely described in the literature for their diverse biological activities, including well-documented antimicrobial effects^8^—heterocycles containing sulfur, nitrogen, or oxygen, such as imidazoles, thiazoles, oxadiazoles, and pyridine rings, have been reported with antimycobacterial activity.^6–9^ Among these, thiazole derivatives have been developed and reported for their antimicrobial properties,^6,10,11^ as well as other biological activities such as anti-inflammatory, analgesic, antitumor, antihypertensive, antitrypanocidal, and antimalarial effects.^11,12^ Consequently, numerous researchers have investigated the antimycobacterial potential of thiazole derivatives, with particular emphasis on 2-aminothiazole compounds.^6,10,11,13^

Thiazole-based compounds have attracted considerable attention in medicinal chemistry due to their wide range of biological activities, particularly their promising antimicrobial potential.^14,15^ The thiazole ring, a five-membered heterocycle containing both sulfur and nitrogen atoms, plays a crucial role in modulating the physicochemical and pharmacokinetic properties of bioactive molecules. Its electron-rich structure and conformational rigidity enable favorable interactions with various biological targets, including bacterial enzymes and membrane components.^16^ The thiazole ring can form hydrogen bonds and coordination interactions with metalloenzymes, which provide an increased affinity for microbial targets, while substitutions that enhance lipophilicity may favor permeation through the hydrophobic barrier of the mycobacterial cell wall. These structural features may contribute to the potential antibacterial activity of thiazole derivatives, especially against resistant strains. Thus, the hydrophobicity of thiazole compounds appears to be a relevant factor in overcoming the mycobacterial lipid barrier and improving their therapeutic efficacy.^17^ NTM, including *M. abscessus*, *Mycobacterium massiliense*, *M. fortuitum*, and *Mycobacterium smegmatis*, pose an increasing clinical challenge due to their intrinsic resistance to conventional antibiotics and their capacity to form robust biofilms.^18,19^ These organisms possess a thick, hydrophobic cell wall rich in long-chain mycolic acids, which severely limits drug permeability and contributes to their persistence in hostile environments.^20^ Given this context, the thiazole ring emerges as a promising pharmacophore in the rational design of new antimycobacterial agents.^21^ Furthermore, our research group has gained experience in the chemical modification of eugenol, a naturally occurring phenylpropanoid known for its wide range of biological activities, and which has demonstrated notable antimicrobial activity.^9,12,22–30^

This study reports the synthesis and biological evaluation of a series of thiazoles (compounds **11**–**26**), along with four thiosemicarbazone derivatives and their precursors (compounds **1**–**10**) obtained from eugenol and eugenol analogues. These compounds were evaluated for antimicrobial activity against clinically relevant NTM species and for their antibiofilm properties. The correlation between structural features and biological activity is discussed, with particular emphasis on the role of the thiazole ring in enhancing efficacy against these difficult-to-treat pathogens.

## 2 Results and Discussion

### 2.1 Chemistry

The new thiazoles were synthesized using classical methods, as shown in Figure 1. The nature of R_1_, R_2_, and R_3_ in the synthesized compounds is presented in Table 1.

**Figure 1:**
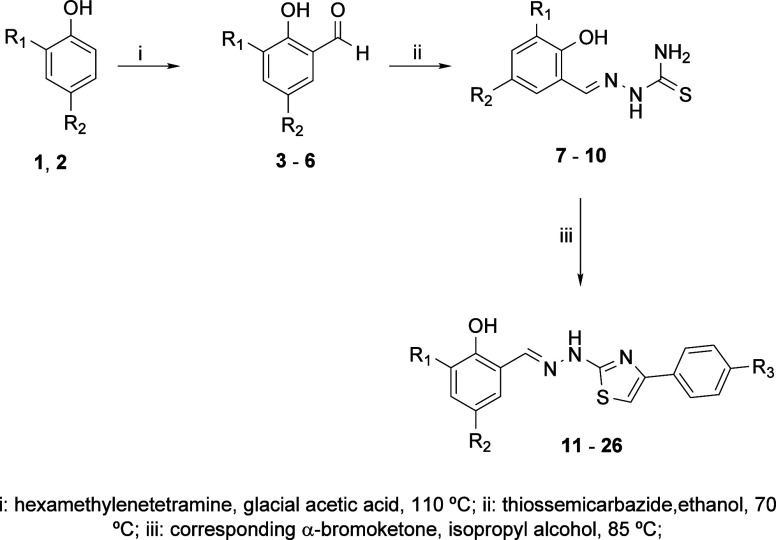
Synthesis of new thiazole compounds.

**Table 1:** Corresponding R_1_, R_2_, and R_3_ for Each Substance (**1**–**26**)

compound	R_1_	R_2_	R_3_	compound	R_1_	R_2_	R_3_
**1**	OCH_3_	allyl		**14**	H	H	H
**2**	OCH_3_	propyl		**15**	OCH_3_	allyl	OCH_3_
**3**	OCH_3_	allyl		**16**	OCH_3_	propyl	OCH_3_
**4**	OCH_3_	propyl		**17**	OCH_3_	H	OCH_3_
**5**	OCH_3_	H		**18**	H	H	OCH_3_
**6**	H	H		**19**	OCH_3_	allyl	Cl
**7**	OCH_3_	allyl		**20**	OCH_3_	propyl	Cl
**8**	OCH_3_	propyl		**21**	OCH_3_	H	Cl
**9**	OCH_3_	H		**22**	H	H	Cl
**10**	H	H		**23**	OCH_3_	allyl	NO_2_
**11**	OCH_3_	allyl	H	**24**	OCH_3_	propyl	NO_2_
**12**	OCH_3_	propyl	H	**25**	OCH_3_	H	NO_2_
**13**	OCH_3_	H	H	**26**	H	H	NO_2_

There are several methodologies in the literature for the synthesis of thiazoles, including those named after Cook–Heilsborn, Gabriel, and Hantzch.^31^ The Cook–Heilsborn synthesis is employed for the preparation of 5-aminothiazoles, while the Gabriel synthesis is typically used to obtain 2-, 5-, or 2,5-disubstituted alkyl, aryl, or alkoxy-thiazoles. On the other hand, the Hantzch synthesis involves the cyclocondensation of a thioamide with an α-bromoketone, facilitating the formation of iminothiazole derivatives.^32^

For the present study, the Hantzch synthesis was selected. The synthesis began with the preparation of formyleugenol (**3**) and formyldihydroeugenol (**4**) through the well-established Duff reaction.^33^ Subsequently, the thiosemicarbazones **7**, **8**, **9**, and **10** were synthesized by reacting these formylated compounds with two commercially available aldehydes, *o*-vanillin and salicylaldehyde, in ethanol under heating conditions, as described by Haque and Salam.^34^ Finally, the target thiazole derivatives (**11**–**26**) were obtained by reacting to thiosemicarbazones **7**, **8**, **9**, or **10** with various α-bromoketone in isopropanol under heat, following the procedure outlined by Fonseca et al.^35^ In this way, four compounds were commercially acquired from Sigma-Aldrich as starting materials. Eugenol and dihydroeugenol were purchased for the synthesis of formyl derivatives, and *o*-vanillin and salicylaldehyde were purchased for the synthesis of tiosemicarbazone derivatives. As a result, twenty-two compounds were synthesized, with the final twenty-six compounds to be biologically evaluated.

The synthesized derivatives were obtained in good yields (45–100%), further studies can be done to improve some of these yields. The spectral data confirmed the formation of the designed products. For instance, the infrared (IR) spectrum of compound **11** did not display the band at 1530 cm^–1^, which is characteristic of the C=S bond, suggesting the successful formation of the thiazole ring. Additionally, a band at 1624 cm^–1^, indicative of the C=N bond, was observed. The ^1^H and ^13^C nuclear magnetic resonance (NMR) spectra of compound **11** further corroborated the formation of the heterocyclic structure. In the ^1^H NMR spectrum, a singlet at δ 7.92 was assigned to the imine proton, and a singlet at δ 6.79 corresponded to the hydrogen of the thiazole ring. Furthermore, multiplets in the ranges δ 7.76–7.73 and δ 7.46–7.36 were observed, which were attributed to the aromatic hydrogens directly attached to the thiazole ring. In the ^13^C NMR spectrum, signals at δ 168.8, 147.2, and 102.2 were assigned to the carbons of the thiazole ring, while the signal at δ 149.2 was attributed to the imine carbon. For the other final products, similar patterns were observed, with slight variations in the chemical shifts due to the different substituents on the aromatic ring.

### 2.2 X-ray Diffraction Analyses

Figure 2 presents the crystallographic structure of compound **19**, which crystallized in the monoclinic system, specifically in the *P*2_1_/*n* space group. The presence of two molecules in the asymmetric unit arises from distinct conformers. The structure unequivocally confirms the (*E*) relative stereochemistry of the imine double bond (N=CH), labeled as C10A–N3A and C10–N3.

**Figure 2:**
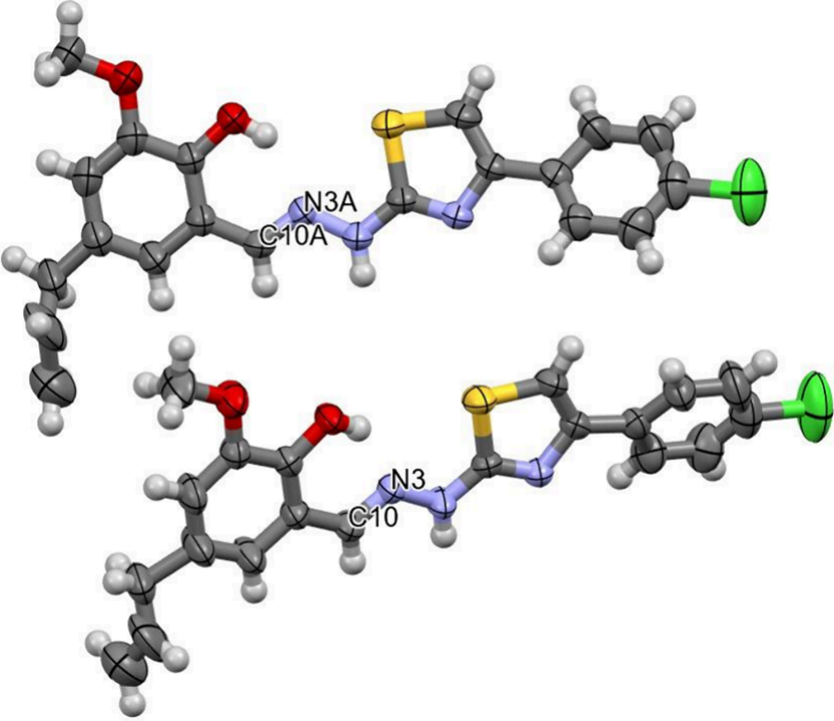
Asymmetric unit of compound **19** elucidated in the present study.

### 2.3 Antimycobacterial Results

Full details of the procedures relating to biological assessments can be found in the Section Cytotoxicity Activity. The antimycobacterial potential of the synthesized compounds was evaluated using the broth microdilution assay, following the guidelines established by the Clinical & Laboratory Standards Institute (CLSI, 2021).^36^ Standard strains of *M. abscessus* (ATCC 19977), *M. massiliense* (ATCC 48898), *M. fortuitum* (ATCC 6841), and *M. smegmatis* (ATCC 700084) were employed in this study. The results are summarized in Table 2.

**Table 2:** Minimum Inhibitory Concentration (MIC) Values of Compounds **1**–**26** against Different Species of Rapidly Growing Mycobacteria (RGM)

	MIC_50_ (μmol L^–1^)			
compound	*M. abcessus*	*M. massiliense*	*M. fortuitum*	*M. smegmatis*
**1**	1142.7	2285.4	2285.4	2285.4
**2**	1128.8	1128.8	1128.8	1128.8
**3**	488.1	488.1	488.1	488.1
**4**	120.7	120.7	120.7	120.7
**5**	77.0	77.0	77.0	154.1
**6**	192.0	95.9	95.9	192.0
**7**	176.8	176.8	176.8	176.8
**8**	175.5	175.5	175.5	175.5
**9**	833.1	1666.2	833.1	833.1
**10**	1922.6	1922.6	1922.6	1922.6
**11**	513.5	513.5	513.5	513.5
**12**	510.7	510.7	510.7	510.7
**13**	288.4	288.4	288.4	288.4
**14**	79.4	79.4	79.4	79.4
**15**	949.1	949.1	949.1	949.1
**16**	944.2	944.2	944.2	944.2
**17**	66.0	66.0	66.0	132.0
**18**	36.0	36.0	36.0	36.0
**19**	117.4	117.4	117.4	117.4
**20**	116.8	116.8	116.8	116.8
**21**	65.3	65.3	65.3	65.3
**22**	1139.7	1139.7	1139.7	1139.7
**23**	914.4	914.4	914.4	914.4
**24**	909.9	909.9	909.9	909.9
**25**	1013.3	1013.3	1013.3	1013.3
**26**	1102.7	1102.7	1102.7	1102.7
clarithromycin	31.32^a^	62.66^a^	1.95	7.82
sulfamethoxazole	46.23	740.29^a^	740.29^a^	185.05

a Resistance profile according to CLSI breakpoints.

The broth microdilution technique, considered the gold standard for determining minimum inhibitory concentration (MIC) values of various compounds, is widely employed in clinical microbiology to assess the susceptibility or resistance of infectious agents to antimicrobial agents used in pharmacological therapies.^36,37^

Sulfamethoxazole and clarithromycin, two drugs commonly employed in the treatment of infections caused by *Mycobacterium* spp., were used as reference standards to assess antimycobacterial activity. The results revealed distinct susceptibility profiles among the four tested strains. According to CLSI breakpoints, *M. fortuitum* and *M. smegmatis* were susceptible to clarithromycin, while *M. abscessus* and *M. massiliense* exhibited resistance.^36^

In the case of sulfamethoxazole, CLSI guidelines indicate that *M. fortuitum* and *M. massiliense* were resistant, whereas *M. abscessus* and *M. smegmatis* remained susceptible. These results are consistent with previous susceptibility data reported by Flores et al.^38^ and Siqueira et al.,^39^ both in standard strains and clinical isolates of RGM. Given the increasing antimicrobial resistance observed in these pathogens, the pursuit of new molecules with antimycobacterial activity becomes imperative.

Overall, the tested thiazole derivatives exhibited a consistent pattern of activity across all four mycobacterial strains. Derivatives containing electron-donating substituents, such as methoxy and chloro groups, demonstrated superior activity, while nitro-substituted analogues showed reduced efficacy. Thiosemicarbazones derived from eugenol (**7**) and dihydroeugenol (**8**) displayed moderate activity, whereas compounds **9** and **10** were less effective. Notably, the most potent antimycobacterial activity was observed in the final thiazole derivatives, underscoring the relevance of the thiazole moiety in enhancing bioactivity.

Among them, compounds **17** and **18**, both featuring *para*-methoxy substitutions, demonstrated activity against all tested *Mycobacterium* species. Compound **18** exhibited the lowest MIC across the series. Similarly, compound **21** showed MIC values comparable to those of **17**, with the notable advantage of high activity against *M. smegmatis*, which was unresponsive to compound **17**. Additionally, compound **14** also stood out by exhibiting broad-spectrum activity across all strains.

Importantly, the *M. massiliense* standard strain—despite its resistance to both clarithromycin and sulfamethoxazole—was susceptible to several thiazole derivatives, particularly **14**, **17**, **18**, and **21**, which yielded promising MIC values. A similar trend was observed for *M. abscessus*, which was resistant to clarithromycin but exhibited sensitivity to the thiazole derivatives, often at MICs equal to or lower than that of sulfamethoxazole.

Likewise, *M. fortuitum* exhibited resistance to sulfamethoxazole yet remained sensitive to the thiazole compounds, particularly **14**, **17**, **18**, and **21**. Even though *M. smegmatis* did not show resistance to the reference drugs, thiazole derivatives still demonstrated strong activity, with compound **18** achieving a MIC value five times lower than that of sulfamethoxazole.

The most active compounds—**7**, **8**, **14**, **17**, **18**, **19**, **20**, and **21**—were selected for further biological evaluation, including time-kill kinetic assays and antibiofilm testing. Time-kill assays were performed using *M. smegmatis* as a model, with compounds tested at 0.5×, 1×, and 2× their MICs over a 72-h period (Figure 3). This strain was chosen as a representative model of RGM, based on genomic and biochemical similarities reported in the literature.^40^ The assay allowed us to monitor bacterial viability over time and assess bactericidal versus bacteriostatic effects.

**Figure 3:**
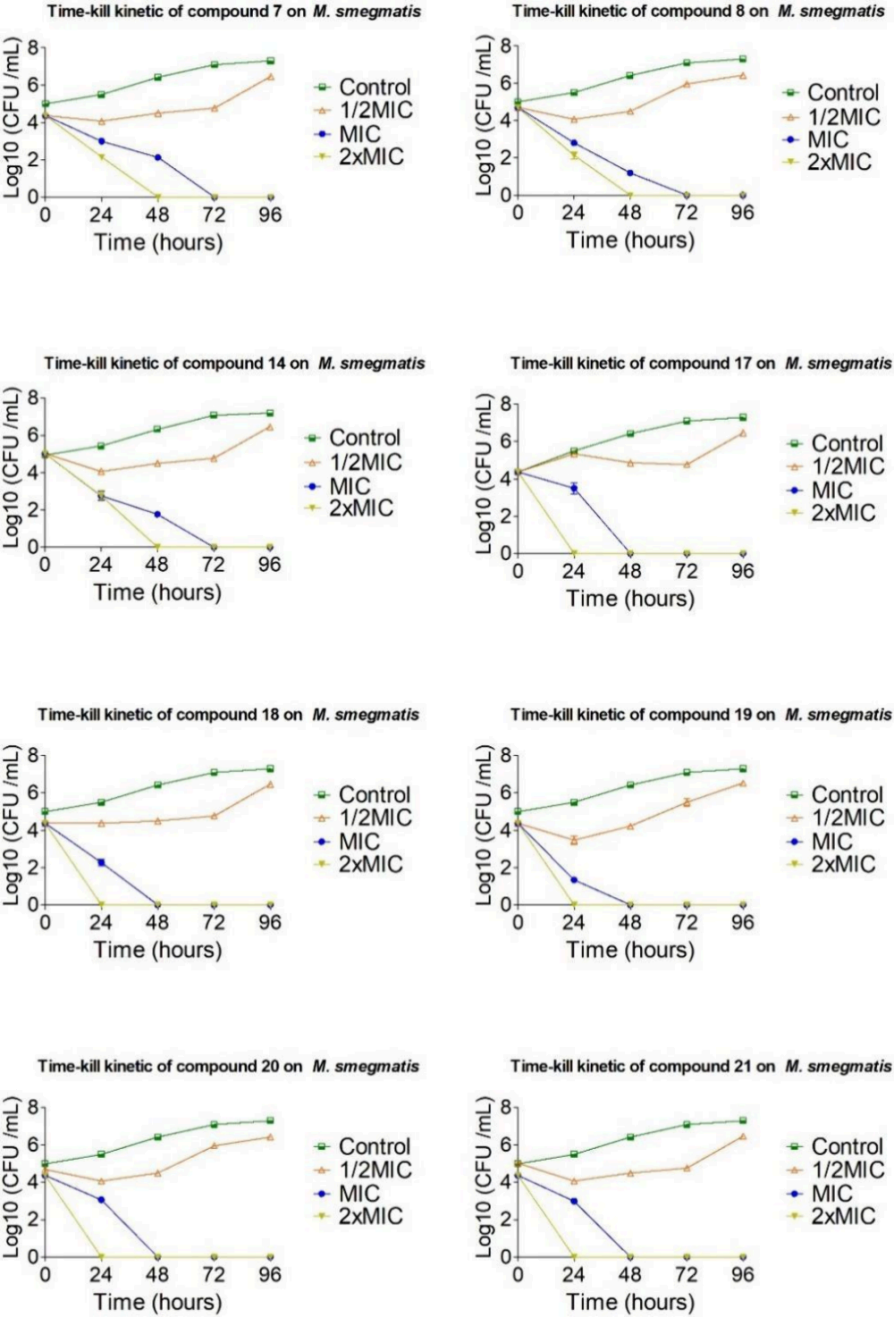
Time-kill kinetic curves for the substances **7**, **8**, **14**, **17**, **18**, **19**, **20**, and **21**.

The time-kill data confirmed that all tested compounds exhibited bactericidal activity at their respective MICs, leading to complete eradication of *M. smegmatis* within 72 h. Notably, compounds **17**, **18**, **19**, **20**, and **21** achieved this outcome within just 48 h, and within 24 h when tested at 2× MIC. These findings are consistent with the MIC results, reinforcing the superior bactericidal performance of compounds **18** and **21**, which showed the lowest MIC values across the tested series.

Due to the intrinsic ability of mycobacteria to form biofilms, complex structures composed of cellular aggregates embedded in a self-produced matrix of exopolysaccharides, the search for effective inhibitors or disruptors of these formations remains a key focus in antimicrobial research. Accordingly, compounds **7**, **8**, **14**, **17**, **18**, **19**, **20**, and **21** were evaluated for their antibiofilm properties. As *M. smegmatis* is not a biofilm-forming species, the assays were conducted using *M. massiliense*, which has demonstrated robust biofilm-forming capacity.

To evaluate the antibiofilm potential of the selected compounds, subinhibitory concentrations (below the MIC) were used to assess the inhibition of biofilm formation. In parallel, concentrations above the MIC were employed to investigate the compounds’ capacity to disrupt pre-established biofilms, promoting the dispersal of sessile microbial communities.

Biofilm formation is a multistep process, beginning with irreversible adhesion of bacterial cells to a surface, followed by microcolony organization and maturation into a structured biofilm, which may persist or undergo dispersal.^41,42^ Antibiofilm strategies generally target one or more of the following mechanisms:^1^ prevention of initial adhesion to surfaces, thereby halting biofilm development;^2^ inhibition of intercellular communication, or quorum sensing, to block biofilm maturation; and^3^ degradation of mature biofilms, leading to dispersion of microbial cells.^41,43^

Our findings allowed for the evaluation of each compound’s ability to either inhibit biofilm development (strategies 1 and 2) or disrupt established biofilms (strategy 3). While further mechanistic studies are warranted to elucidate the exact modes of action, data presented in Figure 3 indicate that all tested compounds significantly inhibited biofilm formation at 1/4 MIC, yielding biomass levels comparable to the negative control (culture medium only). The use of subinhibitory concentrations ensured that planktonic cells remained viable, confirming that the observed effects were due to inhibition of biofilm formation rather than bacteriostatic or bactericidal activity.

Conversely, none of the compounds demonstrated sufficient efficacy in eradicating mature mycobacterial biofilms. As shown in Figure 4, even at 2× MIC, the residual biofilm biomass remained comparable to that of the untreated positive control. This outcome highlights the inherent difficulty in disrupting mature biofilms, particularly those formed by mycobacteria. The highly hydrophobic biofilm matrix and the presence of abundant mycolic acids—long-chain fatty acids in the mycobacterial cell wall—pose significant barriers to the penetration and efficacy of hydrophilic molecules.^43,44^

**Figure 4:**
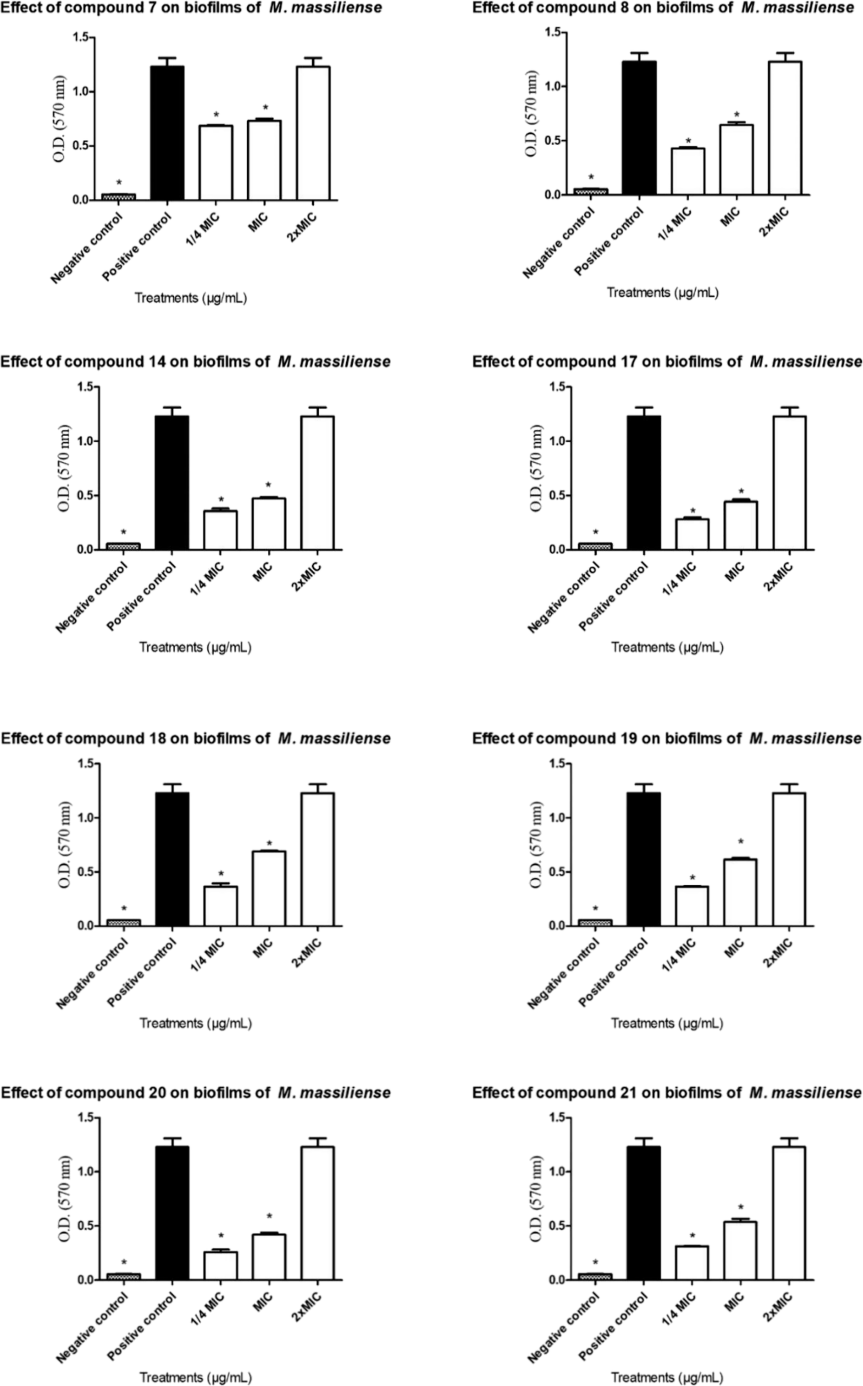
Antibiofilm activity of compounds **7**, **8**, **14**, **17**, **18**, **19**, **20**, and **21**.

### 2.4 Cytotoxicity Activity

Considering the promising results related to the antimycobacterial and antibiofilm activities, the toxicity of the selected compounds (**7**, **8**, **14**, **17**, **18**, **19**, **20**, and **21**) to mammalian cells was evaluated using both human peripheral blood mononuclear cells (PBMC) and the Vero cell line. Interestingly, most compounds exhibited little or no cytotoxicity against both cell types at concentrations up to 400 μmol L^–1^ (Table 3). Only compounds **14**, **19**, and **21** induced a reduction in cell viability, and in a concentration-dependent manner, with 50% cytotoxic concentration (CC_50_) observed in the high micromolar range (Table 3).

**Table 3:** Cytotoxicity of Compounds on PBMC and Vero Cells

	CC_50_ (μmol L^–1^)^a^		selectivity index^b^			
compound	PBMC	Vero	*M. abcessus*	*M. massieliense*	*M. fortuitum*	*M. smegmatis*
**7**	>400	>400	2.26	2.26	2.26	2.26
**8**	>400	>400	2.29	2.29	2.29	2.29
**14**	>400	383.7	5.04	5.04	5.04	5.04
**17**	>400	>400	6.06	6.06	6.06	3.03
**18**	>400	>400	11.10	11.10	11.10	11.10
**19**	291.4	>400	2.48	2.48	2.48	2.48
**20**	>400	>400	3.42	3.42	3.42	3.42
**21**	337.1	>400	5.17	5.17	5.17	5.17

a CC_50_: Concentration that reduces cell viability by 50% in a 48 h assay; CC_50_ were determined on PBMC and Vero cells.

b Selectivity index was calculated using CC_50_ values from PBMC.

Importantly, the following CC_50_ values are higher than the MIC tested against the microorganisms confirmed by the determination of the selectivity index (SI). The most selective compound was the *para*-methoxy substituted derivative **18**, with an SI exceeding 11 for all the mycobacteria studied (Table 3). This compound also exhibited an excellent toxicity profile in the time-kill and antibiofilm assays, as the active 2× MIC concentration used (72 μmol L^–1^) was completely innocuous to mammalian cells. Furthermore, it is noteworthy that compounds **17** and **18**, which showed excellent results in the antimycobacterial and antibiofilm assays, did not display any toxicity against PBMC and Vero cells at the evaluated concentrations.

## 3 Conclusions

After evaluating the 16 thiazoles and the four thiosemicarbazones derivatives, compounds **7**, **8**, **14**, **17**, **18**, **19**, **20**, and **21** showed good antimycobacterial activity against *M. abscessus*, *M. massiliense*, *M. fortuitum*, and *M. smegmatis*, with bactericidal activity within 48 h and inhibition of biofilm formation. Among them, the thiazole derivatives **18** (MIC = 36 μmol L^–1^) exhibited the lowest MIC values against all evaluated species, with results better than the reference drugs and no cytotoxic effects in the cytotoxicity assays, showing high selectivity toward mycobacterial cells. Notably, compound **17** is a new substance, as are **8**, **19**, and **20**, which also showed promising results in all the assays conducted. These findings emphasize the high antimycobacterial potential of the evaluated substances, warranting further studies to explore their therapeutic applications.

## 4 Materials and Methods

### 4.1 Synthesis and Characterization

#### 4.1.1 General

All reagents were obtained from Sigma-Aldrich and used without further purification. Melting points were determined using an Aaker PFM-II apparatus and are reported without correction. Infrared (IR) spectra were recorded on a Thermo Scientific Nicolet iS50 FT-IR spectrometer. Nuclear magnetic resonance (NMR) spectra were acquired on a Bruker AVANCE DRX 300 MHz spectrometer, operating at 300 MHz for ^1^H and 75 MHz for ^13^C, using deuterated dimethyl sulfoxide (DMSO-*d*_6_) as the solvent. Chemical shifts (δ) are reported in parts per million (ppm) relative to tetramethylsilane (TMS) as the internal standard. Coupling constants (*J*) are given in Hertz (Hz), and the following abbreviations were used to describe signal multiplicities: singlet (s), doublet (d), double doublet (dd), triplet (t), triple triplet (tt), and multiplet (m). The progress of the reactions and the purity of the products were monitored by thin-layer chromatography (TLC) using silica gel 60 F254 plates. Purification by column chromatography was carried out on silica gel 60 (230–400 mesh, Sigma-Aldrich). High-resolution mass spectrometry (HRMS) analyses were performed using a Bruker LC/MS-ESI-QTOF system, with samples dissolved in methanol and introduced via direct infusion.

#### 4.1.2 General Synthetic Procedure for Compound **1**

A solution of hexamethylenetetramine (30.4 mmol) in acetic acid (40 mL) was stirred at room temperature for 15 min. Subsequently, eugenol or dehydroeugenol (6.1 mmol) was added, and the reaction mixture was refluxed under continuous monitoring by thin-layer chromatography (TLC). Upon completion of the reaction, the mixture was cooled to room temperature, followed by the addition of concentrated HCl (12.2 mmol) with stirring for 40 min. The resulting mixture was then neutralized with solid NaHCO_3_ to pH 7, and the aqueous phase was extracted with dichloromethane (3 × 100 mL). The combined organic layers were dried over anhydrous Na_2_SO_4_ and concentrated under reduced pressure to remove the solvent. The crude product was used directly in the subsequent synthetic step without further purification.

#### 4.1.3 General Synthetic Procedure for Compound **2**

A solution of aldehydes **3**, **4**, **5**, or **6** (1.03 mmol) in ethanol (3.5 mL) was prepared in a 25 mL round-bottom flask. In a separate flask, thiosemicarbazide (1.03 mmol) was dissolved in absolute ethanol with the addition of a few drops of water (∼15 drops) under heating until complete dissolution. The thiosemicarbazide solution was then added dropwise to the ethanolic solution of the aldehyde. The resulting reaction mixture was refluxed and monitored by thin-layer chromatography (TLC) until complete consumption of the aldehyde. Upon completion, the reaction mixture was cooled to room temperature, and the resulting precipitation was isolated by vacuum filtration and dried under reduced pressure.

##### 4.1.3.1 (*E*)-2-(5-Allyl-2-hydroxi-3-methoxibenzylidene)­hydrazinecarbothioamide (**7**)

This product was obtained in 40% yield as a white solid. mp 222–224 °C. IR (υ̅/cm^–1^) 3457, 3430, 3351, 3313, 3152, 2962, 1609, 1578, 1453, 1433, 1531, 1109, 1268, 1059. ^1^H NMR (DMSO-*d*_6_, 300 MHz) δ 11.37 (s, 1H, OH), 9.01 (s, 1H, =NNH), 8.37 (s, 1H, H–C=N), 8.12 (s, 1H, NH_2_), 7.86 (s, 1H, NH_2_ ), 7.36 (s, 1H, Ar–H), 6.78 (d, 2H, Ar–H, ^4^ *J* = 1.8 Hz), 6.04–5.91 (m, 1H, allyl), 5.10–5.00 (m, 2H, allyl), 3.79 (s, 3H, OCH_3_), 3.27 (d, 2H, allyl, ^3^ *J* = 6.9 Hz). ^13^C NMR (DMSO-*d*_6_, 75 MHz) δ 177.6 (1C, C=S), 147.8 (Ar.), 144.3 (Ar.), 139.5 (1C, HC=N), 138.1 (1C, allyl), 130.5 (Ar.), 120.4 (1C, Ar.), 117.5 (Ar.) 115.4 (1C, allyl), 113.2 (Ar.), 55.8 (1C, OCH_3_), 39.2 (1C, allyl).

##### 4.1.3.2 (*E*)-2-(2-Hydroxi-3-methoxi-5-propylbenzylidene)­hydrazinecarbothioamide (**8**)

This product was obtained in 56% yield as a white solid. Mp 279–284 °C. IR (υ̅/cm^–1^) 3453, 3432, 3345, 3310, 3147, 2953, 1608, 1579, 1489, 1460, 1529, 1110, 1270, 1060. ^1^H NMR (DMSO-*d*_6_, 300 MHz) δ 11.35 (s, 1H, OH), 8.92 (s, 1H, =NNH), 8.37 (s, 1H, H–C=N), 8.10 (s, 1H, NH_2_ ), 7.89 (s, 1H, NH_2_ ), 7.36 (s, 1H, Ar–H), 6.79 (d, 1H, Ar, ^4^ *J* = 1.8 Hz), 3.79 (s, 3H, OCH_3_), 2.49–2.44 (m, 2H, propyl), 1.58 (sex, 2H, propyl, ^3^ *J* = 7.5 Hz), 0.89 (t, 3H, propyl, ^3^ *J* = 7.2 Hz). ^13^C NMR (DMSO-*d*_6_, 75 MHz) δ 177.6 (C=S), 147.7 (Ar.), 144.1 (Ar.), 139.7 (HC=N), 132.9 (Ar.), 120.2 (Ar.), 117.3 (Ar.), 113.2 (Ar.), 55.8 (OCH_3_), 37.0 (propyl), 24.3 (propyl), 13.7 (propyl). HRMS-ESI calcd for C_12_H_18_N_3_O_2_S [M–H]^−^ 266.0968, found 266.0962.

##### 4.1.3.3 (*E*)-2-(2-Hydroxi-3-methoxibenzylidene)­hydrazinecarbothioamide (**9**)

This product was obtained in 93% yield as a white solid. Mp 189–191 °C. IR (υ̅/cm^–1^) 3456, 3337, 3152, 1585, 1479, 1457, 1529, 1113, 1278, 1054. ^1^H NMR (DMSO-*d*_6_, 300 MHz) δ 11.39 (s, 1H, OH), 9.17 (s, 1H, =NNH), 8.39 (s, 1H, HC=N), 8.09 (s, 1H, NH_2_ ), 7.87 (s, 1H, NH_2_ ), 7.52 (d, 1H, Ar–H, ^3^ *J* = 7.8 Hz), 6.95 (dd, 1H, Ar–H, ^3^ *J* = 8.1 Hz; ^4^ *J* = 1.2 Hz), 6.76 (t, 1H, Ar–H, ^3^ *J* = 7.8 Hz), 3.80 (s, 3H, OCH_3_). ^13^C NMR (DMSO-*d*_6_, 75 MHz) δ 177.7 (1C, C=S), 147.9 (1C, Ar.), 145.9 (1C, Ar.), 139.5 (1C, HC=N), 120.8 (1C, Ar.), 119.0 (1C, Ar.), 118.1 (1C, Ar.), 112.8 (1C, Ar.), 55.9 (1C, OCH_3_).

##### 4.1.3.4 (*E*)-2-(2-Hydroxibenzylidene)­hydrazinecarbothioamide (**10**)

This product was obtained in 88% yield as a white solid. Mp 268–272 °C. IR (υ̅/cm^–1^) 3440, 3314, 3134, 1601, 1488, 1461, 1536, 1109. ^1^H NMR (DMSO-*d*_6_, 300 MHz) δ 11.36 (s, 1H, OH), 9.88 (s, 1H, =NNH), 8.37 (s, 1H, H–C=N), 8.08 (s, 1H, NH_2_ ), 7.92 (s, 1H, NH_2_ ), 7.89 (s, 1H, Ar–H), 7.24–7.18 (m, 1H, Ar–H), 6.87–6.78 (m, 2H, Ar–H). ^13^C NMR (DMSO-*d*_6_, 75 MHz) δ 177.7 (1C, C=S), 156.4 (1C, Ar.), 139.7 (HC=N), 131.11 (1C, Ar.), 126.8 (1C, Ar.), 120.4 (1C, Ar.), 119.3 (1C, Ar.), 116.0 (1C, Ar.).

#### 4.1.4 General Synthetic Procedure for Compound **3**

A solution of compounds **7**, **8**, **9**, or **10** (0.75 mmol) in isopropyl alcohol (20 mL) was prepared and stirred under heating for 5 min. Subsequently, α-bromoketone (0.75 mmol) was added, and the reaction progress was monitored by thin-layer chromatography (TLC). Upon complete consumption of the starting material, the reaction mixture was cooled to 5 °C, and the resulting precipitate was collected by vacuum filtration. The solid was washed successively with 10% aqueous sodium bicarbonate (NaHCO_3_) solution and distilled water, then dried under reduced pressure.

##### 4.1.4.1 (*E*)-4-Allyl-2-methoxi-6-((2-(4-phenylthiazol-2-yl)­hydrazone)­methyl)­phenol (**11**)

This product was obtained in 75% yield as a beige solid. Mp 185–189 °C. IR (υ̅/cm^–1^) 3070, 2841, 1624, 1573, 1485, 1458, 1252, 1138. ^1^H NMR (CDCl_3_, 300 MHz) δ 7.92 (s, 1H, H–C=N), 7.76–7.73 (m, 2H, Ar–H), 7.46–7.36 (m, 3H, Ar–H), 6.79 (s, 1H, thiazole), 6.73 (d, 1H, Ar–H, ^4^ *J* = 1.8 Hz), 6.35 (d, 1H, Ar–H, ^4^ *J* = 1.8 Hz), 5.98–5.85 (m, 1H, allyl), 5.12–5.05 (m, 2H, allyl), 3.89 (s, 3H, OCH_3_), 3.28 (d, 2H, allyl, ^3^ *J* = 6.9 Hz). ^13^C NMR (CDCl_3_, 75 MHz) δ 168.8 (1C, thiazole), 149.2 (1C, HC=N), 147.9 (1C, thiazole), 146.6 (1C, Ar.), 145.6 (1C, Ar.), 137.2 (1C, allyl), 131.4 (Ar.), 131.1 (1C, Ar.), 129.6 (1C, Ar.), 129.4 (2C, Ar.), 126.3 (2C, Ar.), 121.6 (1C, Ar.), 117.1 (1C, allyl), 116.2 (1C, Ar.), 114.5 (1C, Ar.), 102.2 (1C, thiazole), 56.3 (1C, OCH_3_), 39.7 (1C, allyl). HRMS-ESI calcd for C_20_H_19_N_3_O_2_S [M–H]^−^ 364.1125, found 364.1110.

##### 4.1.4.2 (*E*)-2-Methoxi-6-((2-(4-phenylthiazol-2-yl)­hydrazone)­methyl)-4-propylphenol (**12**)

This product was obtained in 92% yield as a white solid. Mp 195–200 °C with degradation. IR (υ̅/cm^–1^) 3311, 3058, 2954, 2927, 2856, 1617, 1585, 1480, 1267, 1110. ^1^H NMR (DMSO-*d*_6_, 300 MHz) δ 8.37 (s, 1H, H–C=N), 7.85–7.82 (m, 2H, Ar–H), 7.43 (t, 2H, Ar–H, ^3^ *J* = 7.5 Hz), 7.33–7.28 (m, 2H, thiazole and Ar–H), 7.04 (d, 1H, Ar–H, ^4^ *J* = 1.8), 6.81 (d, 1H, Ar–H, ^4^ *J* = 1.8 Hz), 3.80 (s, 3H, OCH_3_), 2.49–2.45 (m, 2H, propyl), 1.57 (sex, 2H, propyl, ^3^ *J* = 7.5 Hz), 0.90 (t, 3H, propyl, ^3^ *J* = 7.2 Hz). ^13^C NMR (DMSO-*d*_6_, 75 MHz) δ 168.1 (1C, thiazole), 149.6 (1C, thiazole), 147.8 (1C, Ar.), 143.7 (1C, Ar.), 140.5 (HC–N), 134.1 (1C, Ar.), 133.0 (1C, Ar.), 128.7 (2C, Ar.), 127.8 (1C, Ar.), 125.6 (2C, Ar.), 119.9 (1C, Ar.), 117.0 (1C, Ar.), 113.1 (1C, Ar.), 103.5 (1C, thiazole), 55.9 (1C OCH_3_), 37.1 (1C, propyl), 24.3 (1C, propyl), 13.8 (1C, propyl). HRMS-ESI calcd for C_20_H_21_N_3_O_2_S [M–H]^−^ 366.1281, found 366.1288.

##### 4.1.4.3 (*E*)-2-Methoxi-6-((2-(4-phenylthiazol-2-yl)­hydrazone)­methyl)­phenol (**13**)

This product was obtained in 45% yield as a beige solid. Mp 194–199 °C. IR. (υ̅/cm^–1^) 3121, 3050, 2991, 2941, 2837, 1604, 1578, 1559, 1458, 1431, 1247, 1134. ^1^H NMR (DMSO-*d*_6_, 300 MHz) δ 8.35 (s, 1H, H–C=N), 7.87–7.84 (m, 2H, Ar–H), 7.40 (t, 2H, Ar–H, ^3^ *J* = 7.2 Hz), 7.32–7.29 (m, 2H, thiazole and Ar–H), 7.24 (dd, 1H, Ar–H, ^3^ *J* = 7.9 Hz, ^4^ *J* = 1.5 Hz), 6.97 (dd, 1H, Ar–H, ^3^ *J* = 7.9 Hz, ^4^ *J* = 1.5 Hz), 6.83 (t, 1H, Ar–H, ^3^ *J* = 7.8 Hz), 3.82 (s, 3H, OCH_3_). ^13^C NMR (DMSO-*d*_6_, 75 MHz) δ 168.0 (1C, thiazole), 150.4 (1C, thiazole), 147.9 (1C, Ar.), 145.5 (1C, Ar.), 139.6 (1C, HC=N), 134.6 (1C, Ar.), 128.6 (2C, Ar.), 127.6 (1C, Ar.), 125.4 (2C, Ar.), 120.5 (1C, Ar.), 119.3 (1C, Ar.), 117.9 (1C, Ar.), 112.5 (1C, Ar.), 103.3 (1C, thiazole), 55.9 (1C, OCH_3_). HRMS-ESI calcd for C_17_H_15_N_3_O_2_S [M–H]^−^ 324.0812, found 324.0795.

##### 4.1.4.4 (*E*)-2-((2-(4-Phenylthiazol-2-yl)­hydrazone)­methyl)­phenol (**14**)

This product was obtained in quantitative yield as a white solid. Mp 214–219 °C com degradação. IR. (υ̅/cm^–1^) 1620, 1558, 1507, 1494, 1463. ^1^H NMR (DMSO-*d*_6_, 300 MHz) δ 8.35 (s, 1H, H–C=N), 7.86–7.83 (m, 2H, Ar–H), 7.63 (dd, 1H, Ar–H, ^3^ *J* = 7.8 Hz, ^4^ *J* = 1.5 Hz), 7.41 (t, 2H, Ar–H, ^3^ *J* = 7.2 Hz), 7.33–7.28 (m, 2H, thiazole and Ar–H), 7.25–7.20 (m, 1H, Ar–H), 6.92–6.86 (m, 2H, Ar–H). ^13^C NMR (DMSO-*d*_6_, 75 MHz) δ 168.0 (1C, thiazole), 156.1 (1C, Ar.), 149.8 (1C, thiazole), 140.4 (1C, HC=N), 134.2 (1C, Ar.), 130.7 (1C, Ar.), 128.7 (2C, Ar.), 127.7 (1C, Ar.), 126.5 (1C, Ar.), 125.6 (2C, Ar.), 120.1 (1C, Ar.), 119.5 (1C, Ar.), 116.2 (1C, Ar.), 103.4 (1C, thiazole). HRMS-ESI calcd for C_16_H_13_N_3_OS [M–H]^−^ 294.0706, found 294.0683

##### 4.1.4.5 (*E*)-4-Allyl-2-methoxi-6-((2-(4-(4-methoxiphenyl)­thiazol-2-yl)­hydrazone)­methyl)­phenol (**15**)

This product was obtained in 80% yield as a beige solid. Mp 231–236 °C with degradation. IR. (υ̅/cm^–1^) 2833, 1578, 1494, 1459, 1250, 1054. ^1^H NMR (DMSO-*d*_6_, 300 MHz) δ 8.27 (s, 1H, HC=N), 7.78 (d, 2H, Ar–H, ^3^ *J* = 8.7 Hz), 7.03 (s, 1H, thiazole), 6.97–6.94 (m, 3H, Ar–H), 6.76 (d, 1H, Ar–H, ^4^ *J* = 1.2 Hz), 6.03–5.89 (m, 1H, allyl), 5.13–5.04 (m, 2H, allyl), 3.80 (s, 3H, OCH_3_), 3.78 (s, 3H, OCH_3_), 3.30 (d, 2H, allyl, ^3^ *J* = 6.6 Hz). ^13^C NMR (DMSO-*d*_6_, 75 MHz) δ 169.4 (1C, thiazole), 158.7 (1C, Ar.), 150.2 (1C, Ar.), 147.8 (1C, thiazole), 143.9 (1C, Ar.), 139.5 (1C, HC=N), 137.9 (1C, allyl), 130.3 (1C, Ar.), 127.7 (1C, Ar.), 126.8 (2C, Ar.), 120.3 (1C, Ar.), 117.6 (1C, Ar.), 115.6 (1C, Ar.), 113.9 (2C, Ar.), 112.5 (1C, allyl), 100.2 (1C, thiazole), 55.8 (1C, OCH_3_), 55.1 (1C, OCH_3_), 39.2 (1C, allyl). HRMS-ESI calcd for C_21_H_21_N_3_O_3_S [M–H]^−^ 394.1230, found 394.1221.

##### 4.1.4.6 (*E*)-2-Methoxi-6-((2-(4-(4-methoxiphenyl)­thiazol-2-yl)­hydrazone)­methyl)-4-propylphenol (**16**)

This product was obtained in 52% yield as a beige solid. Mp 200–204 °C with degradation. IR. (υ̅/cm^–1^) 3065, 2955, 2931, 2833, 1589, 1492, 1459, 1249, 1031. ^1^H NMR (DMSO-*d*_6_, 300 MHz) δ 8.30 (s, 1H, H–C=N), 7.78 (d, 2H, Ar–H, ^3^ *J* = 8.7 Hz), 7.09 (s, 1H, thiazole), 7.01 (d, 1H, Ar–H, ^4^ *J* = 1.8), 6.95 (d, 2H, Ar–H, ^3^ *J* = 8.7 Hz), 6.8 (d, 1H, Ar–H, ^4^ *J* = 1.5 Hz), 3.81 (s, 3H, OCH_3_), 3.78 (s, 3H, OCH_3_), 2.49–2.46 (m, 2H, propyl), 1.58 (sex, 2H, propyl, ^3^ *J* = 7.2 Hz), 0.9 (t, 3H, propyl, ^3^ *J* = 7.2 Hz). ^13^C NMR (DMSO-*d*_6_, 75 MHz) δ 168.1 (1C, thiazole), 158.8 (1C, Ar.), 150.1 (1C, Ar.), 147.8 (1C, thiazole), 143.6 (1C, Ar.), 140.1 (1C, HC=N), 132.9 (1C, Ar.), 127.5 (1C, Ar.), 126.9 (2C, Ar–H), 119.9 (1C, Ar.), 117.03 (1C, Ar.), 113.9 (2C, Ar.), 112.9 (1C, Ar.), 100.9 (1C, thiazole), 55.8 (1C, OCH_3_), 55.1 (1C, propyl), 37.0 (1C, OCH_3_), 24.2 (1C, propyl), 13.7 (1C, propyl). HRMS-ESI calcd for C_21_H_22_N_3_O_3_S [M–H]^−^ 396.1387, found 396.1379.

##### 4.1.4.7 (*E*)-2-Methoxi-6-((2-(4-(4-methoxiphenyl)­thiazol-2-yl)­hydrazone)­methyl)­phenol (**17**)

This product was obtained in 52% yield as a light-brown solid. Mp 170–174 °C. IR. (υ̅/cm^–1^) 3059, 2935, 2835, 1605, 1572, 1492, 1459, 1246, 1028. ^1^H NMR (DMSO-*d*_6_, 300 MHz) δ 8.34 (s, 1H, H–C=N), 7.78 (d, 2H, Ar–H, ^3^ *J* = 9 Hz), 7.24 (dd, 1H, Ar–H, ^3^ *J* = 7.8 Hz, ^4^ *J* = 1.2 Hz), 7.12 (s, 1H, thiazole), 6.98–6.95 (m, 3H, Ar–H), 6.83 (t, 1H, Ar–H, ^3^ *J* = 7.8 Hz), 3.82 (s, 3H, OCH_3_), 3.78 (s, 3H, OCH_3_). ^13^C NMR (DMSO-*d*_6_, 75 MHz) δ 167.9 (1C, thiazole), 158.9 (1C, Ar.), 149.9 (1C, thiazole), 147.9 (1C, Ar.), 145.5 (1C, Ar.), 139.8 (1C, HC=N), 127.3 (1C, Ar.), 126.9 (2C, Ar.), 120.5 (1C, Ar.), 119.2 (1C, Ar.), 117.9 (1C, Ar.), 113.9 (2C, Ar.), 112.5 (1C, Ar.), 101.2 (1C, thiazole), 55.8 (1C, OCH_3_), 55.1 (1C, OCH_3_). HRMS-ESI calcd for C_18_H_17_N_3_O_3_S [M–H]^−^ 354.0917, found 354.0907.

##### 4.1.4.8 (*E*)-2-((2-(4-(4-Methoxiphenyl)­thiazol-2-yl)­hydrazone)­methyl)­phenol (**18**)

This product was obtained in 86% yield as a brown solid. Mp 234–238 °C with degradation. IR. (υ̅/cm^–1^) 3100, 2833, 1621, 1569, 1511, 1492, 1464, 1246, 1030. ^1^H NMR (DMSO-*d*_6_, 300 MHz) δ 8.35 (s, 1H, H–C=N), 7.78 (d, 2H, Ar–H, ^3^ *J* = 8.7 Hz), 7.63 (dd, 1H, Ar–H, ^3^ *J* = 7.6 Hz, ^4^ *J* = 1.2 Hz), 7.25–7.20 (m, 1H, Ar–H), 7.13 (s, 1H, thiazole), 6.98 (d, 2H, Ar–H, ^3^ *J* = 9 Hz), 6.92–6.86 (m, 2H, Ar–H), 3.78 (s, 3H, OCH_3_). ^13^C NMR (DMSO-*d*_6_, 75 MHz) δ 167.9 (1C, thiazole), 158.9 (1C, Ar.), 156.1 (1C, Ar.), 149.5 (1C, thiazole), 140.4 (1C, HC=N), 130.6 (2C, Ar.), 126.9 (2C, Ar.), 126.6 (1C, Ar.), 120.0 (1C, Ar.), 119.5 (1C, Ar.), 116.2 (1C, Ar.), 114.0 (2C, Ar.), 101.2 (1C, thiazole), 55,2 (1C, OCH_3_). HRMS-ESI calcd for C_17_H_15_N_3_O_2_S [M–H]^−^ 324.0812, found 324.0802.

##### 4.1.4.9 (*E*)-4-Allyl-2-((2-(4-(4-chlorophenyl)­thiazol-2-yl)­hydrazone)­methyl)-6-methoxiphenol (**19**)

This product was obtained in 75% yield as a beige solid. Mp 245–249 °C with degradation. IR. (υ̅/cm^–1^) 3068, 2970, 1583, 1478, 1458, 1252, 1090, 1090. ^1^H NMR (DMSO-*d*_6_, 300 MHz) δ 12.16 (s, 1H, OH), 9.33 (s, 1H, NH), 8.32 (s, 1H, H–C=N), 7.86 (d, 2H, Ar–H, ^3^ *J* = 7.5 Hz), 7.45 (d, 2H, Ar–H, ^3^ *J* = 7.5 Hz), 7.38 (s, 1H, thiazole), 7.06 (s, 1H, Ar–H), 6.8 (s, 1H, Ar–H), 6.03–5.90 (m, 1H, allyl), 5.13–5.04 (m, 2H, allyl), 3.80 (s, 3H, OCH_3_), 3.31 (d, 2H, allyl, ^3^ *J* = 5.1 Hz). ^13^C NMR (DMSO-*d*_6_, 75 MHz) δ 168.1 (1C, thiazole), 149.4 (1C, Ar.), 147.9 (1C, thiazole), 143.8 (1C, Ar.), 139.9 (1C, HC=N) 137.9 (1C, allyl), 133.5 (1C, Ar.), 131.9 (1C, Ar.), 130.5 (1C, Ar.), 128.6 (2C, Ar.), 127.2 (2C, Ar.), 120.2 (1C, Ar.), 117.1 (1C, Ar.), 115.7 (1C, Ar.), 112.9 (1C, allyl), 104.2 (1C, thiazole), 55.8 (1C, OCH_3_), 39.2 (1C, allyl). HRMS-ESI calcd for C_20_H_18_ClN_3_O_2_S [M–H]^−^ 398.0735, found 398.0738.

##### 4.1.4.10 (*E*)-2-((2-(4-(4-Chlorophenyl)­thiazol-2-yl)­hydrazone)­methyl)-6-methoxi-4-propylphenol (**20**)

This product was obtained in quantitative yield as a beige solid. Mp 193–196 °C with degradation. IR. (υ̅/cm^–1^) 3175, 3068, 2953, 2928, 2868, 1581, 1567, 1477, 1456, 1252, 1052, 1108. ^1^H NMR (DMSO-*d*_6_, 300 MHz) δ 8.26 (s, 1H, H–C=N), 7.85 (d, 2H, Ar–H, ^3^ *J* = 8.7 Hz), 7.42 (d, 2H, Ar–H, ^3^ *J* = 8.4 Hz), 7.19 (s, 1H, thiazole), 6.90 (d, 1H, Ar–H, ^4^ *J* = 1.2 Hz), 6.74 (d, 1H, Ar–H, ^4^ *J* = 1.2 Hz), 3.79 (s, 3H, OCH_3_), 2.46–2.44 (m, 2H, propyl), 1.56 (sex, 2H, propyl, ^3^ *J* = 7.5 Hz), 0.89 (t, 3H, propyl, ^3^ *J* = 7.2 Hz). ^13^C NMR (DMSO-*d*_6_, 75 MHz) δ 170.9 (1C, thiazole), 149.3 (1C, Ar.), 147.7 (1C, thiazole), 143.8 (1C, Ar.), 139.8 (1C, HC=N), 134.0 (1C, Ar.), 132.6 (1C, Ar.), 131.6 (1C, Ar.), 128.5 (2C, Ar.), 127.2 (2C, Ar.), 120.2 (2C, Ar.), 117.7 (1C, Ar.), 112.4 (1C, Ar.), 102.4 (1C, thiazole), 55.8 (1C, OCH_3_), 37.0 (1C, propyl), 24.2 (1C, propyl), 13.7 (1C, propyl). HRMS-ESI calcd for C_20_H_20_ClN_3_O_2_S [M–H]^−^ 400.0892, found 400.0887.

##### 4.1.4.11 (*E*)-2-((2-(4-(4-Chlorophenyl)­thiazol-2-yl)­hydrazone)­methyl)-6-methoxiphenol (**21**)

This product was obtained in 84% yield as a beige solid. Mp 243–248 °C with degradation. IR. (υ̅/cm^–1^) 3410, 3055, 2935, 2837, 1601, 1577, 1468, 1434, 1246, 1053, 1089. ^1^H NMR (DMSO-*d*_6_, 300 MHz) δ 8.32 (s, 1H, H–C=N), 7.85 (d, 2H, Ar–H, ^3^ *J* = 8.4 Hz), 7.44 (d, 2H, Ar–H, ^3^ *J* = 8.1 Hz), 7.30 (s, 1H, thiazole), 7.19 (d, 1H, Ar–H, ^3^ *J* = 7.8 Hz), 6.94 (d, 1H, Ar–H, ^3^ *J* = 7.8 Hz), 6.81 (t, 1H, Ar–H, ^3^ *J* = 7.8 Hz), 3.81 (s, 3H, OCH_3_). ^13^C NMR (DMSO-*d*_6_, 75 MHz) δ 169.2 (1C, thiazole), 149.3 (1C, thiazole), 148.0 (1C, Ar.), 145.6 (1C, Ar.), 139.6 (1C, HC=N), 133.7 (1C, Ar.), 131.9 (1C, Ar.), 128.6 (2C, Ar.), 127.3 (1C. Ar.), 120.6 (1C, Ar.), 119.2 (1C, Ar.), 118.1 (1C, Ar.), 112.3 (1C, Ar.), 103.6 (1C, thiazole), 55.9 (1C, OCH_3_). HRMS-ESI calcd for C_17_H_13_ClN_3_O_2_S [M–H]^−^ 358.0402, found 358.0414.

##### 4.1.4.12 (*E*)-2-((2-(4-(4-Chlorophenyl)­thiazol-2-yl)­hydrazone)­methyl)­phenol (**22**)

This product was obtained in 82% yield as a white solid. Mp 259–265 °C with degradation. IR. (υ̅/cm^–1^) 3314, 3109, 1621, 1579, 1491, 1476, 1111. ^1^H NMR (DMSO-*d*_6_, 300 MHz) δ 10.10 (s, 1H, NH), 8.34 (s, 1H, H–C=N), 7.86 (d, 2H, Ar–H, ^3^ *J* = 8.4 Hz), 7.63 (dd, 1H, Ar–H, ^3^ *J* = 7.5 Hz, ^4^ *J* = 1.2 Hz), 7.45 (d, 2H, Ar–H, ^3^ *J* = 8.7 Hz), 7.37 (s, 1H, thiazole), 7.25–7.19 (m, 1H, Ar–H), 6.92–6.95 (m, 2H, Ar–H). ^13^C NMR (DMSO-*d*_6_, 75 MHz) δ 168.1 (1C, thiazole), 156.0 (1C, Ar.), 149.1 (1C, thiazole), 140.0 (1C, HC=N), 133.4 (1C, Ar.), 132.0 (1C, Ar.), 130.6 (1C, Ar.), 128.6 (2C, Ar.), 127.3 (2C, Ar.), 126.5 (1C, Ar.), 120.1 (1C, Ar.), 119.5 (1C, Ar.), 116.2 (1C, Ar.), 104.2 (1C, thiazole). HRMS-ESI calcd for C_16_H_12_ClN_3_OS [M–H]^−^ 328.0316, found 328.0302.

##### 4.1.4.13 (*E*)-4-Allyl-2-methoxi-6-((2-(4-(4-nitrophenyl)­thiazol-2-yl)­hydrazone)­methyl)­phenol (**23**)

This product was obtained in 97% yield as a yellow solid. Mp 241–246 °C with degradation. IR. (υ̅/cm^–1^) 3098, 2917, 1601, 1632, 1339, 1583, 1513, 1471, 1434, 1251, 1106. ^1^H NMR (DMSO-*d*_6_, 300 MHz) δ 8.35 (s, 1H, H–C=N), 8.27 (d, 2H, Ar–H, ^3^ *J* = 9 Hz), 8.10 (d, 2H, Ar–H, ^3^ *J* = 9 Hz), 7.70 (s, 1H, thiazole), 7.07 (d, 1H, Ar–H, ^4^ *J* = 1.5 Hz), 6.80 (d, 1H, Ar–H, ^4^ *J* = 1.8 Hz), 6.04–5.90 (m, 1H, allyl), 5.14–5.05 (m, 2H, allyl), 3.81 (s, 3H, OCH_3_), 3.31 (d, 2H, allyl, ^3^ *J* = 6.6 Hz). ^13^C NMR (DMSO-*d*_6_, 75 MHz) δ 168.4 (1C, thiazole), 148.5 (1C, Ar.), 147.9 (1C, thiazole), 146.2 (1C, Ar.), 143.8 (1C, Ar.), 140.6 (1C, HC=N), 139.8 (1C, Ar.), 137.9 (1C, allyl), 130.5 (1C, Ar.), 126.4 (2C, Ar.), 124.1 (2C, Ar.), 120.2 (1C, Ar.), 116.9 (1C, Ar.), 115.7 (1C, Ar.), 113.0 (1C, thiazole), 108.3 (1C, thiazole), 55.8 (1C, OCH_3_). HRMS-ESI calcd for C_20_H_18_N_4_O_4_S [M–H]^−^ 409.0976, found 409.0970

##### 4.1.4.14 (*E*)-2-Methoxi-6-((2-(4-(4-nitrophenyl)­thiazol-2-yl)­hydrazone)­methyl)-4-propylphenol (**24**)

This product was obtained in quantitative yield as a light-yellow solid. Mp 226–230 °C. IR. (υ̅/cm^–1^) 3345, 2959, 2930, 2871, 1602, 1633, 1338, 1583, 1521, 1251, 1107. ^1^H NMR (DMSO-*d*_6_, 300 MHz) δ 8.34 (s, 1H, H–C=N), 8.26 (d, 2H, Ar–H, ^3^ *J* = 9 Hz), 8.10 (d, 2H, Ar–H, ^3^ *J* = 9 Hz), 7.69 (s, 1H, thiazole), 7.05 (d, 1H, Ar–H, ^4^ *J* = 1.5 Hz), 6.81 (d, 1H, Ar–H, ^4^ *J* = 1.5 Hz), 3.81 (s, 3H, OCH_3_), 2.49–2.46 (m, 2H, propyl), 1.59 (sex, 2H, propyl, ^3^ *J* = 7.5 Hz), 0.91 (t, 3H, propyl, ^3^ *J* = 7.2 Hz). ^13^C NMR (DMSO-*d*_6_, 75 MHz), 168.4 (1C, thiazole), 148.5 (1C, Ar.), 147.9 (1C, thiazole), 143.6 (1C, Ar.), 140.6 (2C, HC=N and Ar.), 140.1 (1C, Ar.), 133.0 (1C, Ar.), 126.4 (2C, Ar.), 124.2 (2C, Ar.), 119.9 (1C, Ar.), 116.9 (1C, Ar.), 113.1 (1C, Ar.), 108.3 (1C, thiazole), 55.9 (1C, OCH_3_), 37.0 (1C, propyl), 24.2 (1C, propyl), 13.7 (1C, propyl). HRMS-ESI calcd for C_20_H_20_N_4_O_4_S [M–H]^−^ 411.1132, found 411.1127.

##### 4.1.4.15 (*E*)-2-Methoxi-6-((2-(4-(4-nitrophenyl)­thiazol-2-yl)­hydrazone)­methyl)­phenol (**25**)

This product was obtained in 93% yield as a light-yellow solid. Mp 292–296 °C with degradation. IR (υ̅/cm^–1^) 3254, 3109, 2937, 2842, 1564, 1599, 1339, 1522, 1508, 1468, 1244, 1049. ^1^H NMR (DMSO-*d*_6_, 300 MHz) δ 12.94 (s, 1H, OH), 9.43 (s, 1H, NH), 8.37 (s, 1H, H–C=N), 8.27 (d, 2H, Ar–H, ^3^ *J* = 8.7 Hz), 8.10 (d, 2H, Ar–H, ^3^ *J* = 9 Hz), 7.70 (s, 1H, thiazole), 7.26 (dd, 1H, Ar–H, ^3^ *J* = 7.8 Hz, ^4^ *J* = 0.9 Hz), 6.97 (dd, 1H, Ar–H, ^3^ *J* = 8.1 Hz, ^4^ *J* = 1.2 Hz), 6.83 (t, 1H, Ar–H, ^3^ *J* = 7.8 Hz), 3.82 (s, 3H, OCH_3_). ^13^C NMR (DMSO-*d*_6_, 75 MHz) δ 168.4 (1C, thiazole), 148.6 (1C, thiazole), 148.0 (1C, Ar.), 146.2 (1C, Ar.), 145.5 (1C, Ar.), 140.6 (1C, HC=N), 139.6 (1C, Ar.), 126.3 (2C, Ar.), 124.1 (2C, Ar.), 120.5 (1C, Ar.), 119.3 (1C, Ar.), 117.6 (1C, Ar.), 112.6 (1C, Ar.), 108.3 (1C, thiazole), 55.8 (1C, OCH_3_). HRMS-ESI calcd for C_17_H_14_N_4_O_4_S [M–H]^−^ 369.0663, found 369.0657.

##### 4.1.4.16 (*E*)-2-((2-(4-(4-Nitrophenyl)­thiazol-2-yl)­hydrazone)­methyl)­phenol (**26**)

This product was obtained in 93% yield as an orange solid. Mp > 300 °C. IR. (υ̅/cm^–1^) 3312, 3111, 1620, 1598, 1522, 1471, 1570, 1318. ^1^H NMR (DMSO-*d*_6_, 300 MHz) δ 12.23 (s, 1H, OH), 10.08 (s, 1H, NH), 8.35 (s, 1H, H–C=N), 8.26 (d, 2H, Ar–H, ^3^ *J* = 8.7 Hz), 8.09 (d, 2H, Ar–H, ^3^ *J* = 8.7 Hz), 7.69 (s, 1H, thiazole), 7.64 (dd, 1H, Ar–H, ^3^ *J* = 7.8 Hz, ^4^ *J* = 1.2 Hz), 7.25–7.20 (m, 1H, Ar–H), 6.92–6.85 (m, 2H, Ar–H). ^13^C NMR (DMSO-*d*_6_, 75 MHz) δ 168.4 (1C, thiazole), 155.9 (1C, Ar.), 148.6 (1C, thiazole), 146.2 (1C, Ar.), 140.6 (1C, HC=N), 139.9 (1C, Ar.), 130.7 (2C, Ar.), 126.3 (2C, Ar.), 124.1 (2C, Ar.), 120.1 (1C, Ar.), 119.5 (1C, Ar.), 116.2 (1C, Ar.), 108.3 (1C, thiazole). HRMS-ESI calcd for C_16_H_12_N_4_O_3_S [M–H]^−^ 339.0557, found 339.0550.

#### 4.1.5 X-ray Diffraction Analysis

Single crystals of **19** were grown by slow evaporation of the solvent from a CH_2_Cl_2_/MeOH solution (2:1 v/v). Suitable crystals were chosen for the X-ray diffraction experiments that were performed with a RigakuXtaLAB mini II diffractometer using graphite-monochromatized Mo-Ka radiation. The Bruker programs SAINT and SADABS^45^ and the CrysalisPro^46^ were used for cell refinement, data indexing, integration, reduction, and absorption correction. Structure solution and refinement were performed using SHELX-2018/3 ^47^ within the OLEX2.^48^ Structure analysis and artwork preparation were performed with Mercury software.^49^ All non-hydrogen atoms of the asymmetric unit were directly located from the electronic density Fourier map, and the free anisotropic thermal displacement parameters were used to refine them. All CH hydrogens were added to their corresponding carbons following a riding model with fixed bond angles and lengths (0.96 Å, 0.97 Å, and 0.93 Å in methyl, methylene, and aromatic groups, respectively). In the case of OH, all hydrogens were constrained with fixed bond lengths of 0.82 Å with a torsion angle from electron density after they were found from the difference Fourier map and checked for the suitable directionality of hydrogen bonds. Hydrogens had their isotropic atomic displacement parameters set to 1.2 Uiso­(C) or 1.5 Uiso­(O).

#### 4.1.6 Biological Evaluations

##### 4.1.6.1 Antimycobacterial Activity

The antimycobacterial activity of the synthesized compounds was evaluated by the broth microdilution method, following CLSI guidelines.^36^ The tested strains included *M. abscessus* (ATCC 19977), *M. massiliense* (ATCC 48898), *M. fortuitum* (ATCC 6841), and *M. smegmatis* (ATCC 700084). Stock solutions of the compounds were prepared in DMSO and subsequently diluted in Mueller–Hinton (MH) broth to final concentrations ranging from 375.0 to 0.09 μg/mL. The bacterial inoculum was standardized using the 0.5 McFarland scale. After incubation at 30 °C for 72 h, the minimum inhibitory concentration (MIC) was determined as the lowest compound concentration that completely inhibited visible mycobacterial growth.

##### 4.1.6.2 Time-Kill Curve

The time-kill assay was conducted using a *M. smegmatis* inoculum standardized to 10^5^ CFU/mL. Selected compounds were tested at concentrations corresponding to MIC, 2× MIC, and 2× MIC. Cultures were incubated at 37 °C for 96 h, with aliquots collected at 0, 24, 48, 72, and 96 h for colony-forming unit (CFU) quantification. Drug-free cultures served as growth controls. Bactericidal activity was assessed by comparing CFU counts of treated versus untreated samples at each time point. The assay was performed in triplicate, following the methodology described by Siqueira and co-workers.^39^

##### 4.1.6.3 Antibiofilm Assay

The ability of the compounds to inhibit biofilm formation by *M. massiliense* was evaluated at subinhibitory concentrations (2× MIC). Polystyrene test tubes (5 mL) were filled with 1 mL of Middlebrook 7H9 medium containing 1 × 10^7^ CFU/mL and 1 mL of compound solution. Tubes were sealed with Parafilm and incubated at 30 °C for 7 days, following the protocol described by Flores and co-workers.^38^

For the biofilm disruption assay, biofilms were first allowed to form under the same conditions. After 7 days, 1 mL of compound (at 2× MIC) was added, and tubes were incubated for an additional 24 h at 30 °C.

Biofilms were quantified by crystal violet staining. Weakly adhered cells were removed by rinsing with saline, and the remaining biofilm was stained with 2 mL of 0.1% crystal violet for 10 min. Excess dye and planktonic cells were removed by washing with saline. The bound dye was solubilized with 2 mL of 95% ethanol for 15 min, and absorbance was measured at 570 nm using a spectrophotometer (U-1800, Hitachi).

Biofilm formation was confirmed by a and significant difference in optical density (OD) between the positive control (medium + bacteria) and the negative control (medium only). Experiments were performed in triplicate. Results are expressed as mean ± standard error (SE), and statistical significance was assessed by Student’s *t* test (*P* > 0.05). Graphs were generated using GraphPad Prism version 8.01.

##### 4.1.6.4 Cytotoxicity Activity

The cytotoxicity of the compounds (**7**, **8**, **14**, **17**, **18**, **19**, **20**, and **21**) to peripheral blood mononuclear cells (PBMCs) and Vero cell line were determined by resazurin method.^50^ The PBMCs were obtained from healthy volunteers by Ficoll–Hypaque density gradient centrifugation as previously described.^51^ Briefly, 2.0 × 10^6^ cells/mL PBMC or 2.0 × 10^3^ cells/mL Vero were distributed in a 96-well plate. The cells were incubated with test compounds ranging from 400 to 25 μmol/L, at 37 °C, 5% CO_2_ for 48 h. After, it was added 10 μL of resazurin Sigma (10 mM) and the cells were incubated again for an additional 6–8 h period. Absorbances were measured in a spectrophotometric microplate reader at 570 and 600 nm and the cell viability were calculated considering the difference in resazurin reduction between treated and nontreated cells. The CC_50_, which corresponds to the concentration that reduces cell viability by 50%, was calculated by nonlinear regression using CompuSyn software. All experiments were run in duplicate or triplicate, and results are averages from at least two independent experiments.

## Supplementary Material



## Notes

Structural characterization data (infrared, NMR, and HRM spectra and X-ray crystallography) (PDF)
