# Supplementary material for: Synthesis and Biological Assessment of New Thiazoles for Mycobacterial Infections and Biofilm Disruption
Source: ACS Omega. 2025 Oct 18;10(42):50007–18. doi: 10.1021/acsomega.5c06421 (PMC12572995; doi:10.1021/acsomega.5c06421)
Supplement: Supplementary file 1 [file ao5c06421_si_001.pdf]

SUPPORTING INFORMATION

Synthesis and biological assessment of new thiazoles for mycobacterial infections and biofilm disruption

*Laís Regina dos Santos Folquitto[a], Fallon dos Santos Siqueira[b], Tayná Roberta Nunes[a], Thiago Belarmino de Souza[c], Diogo Teixeira Carvalho[d], Rafael Pereira Machado[a], Antonio Carlos Doriguetto[a], Marli Matiko Anraku De Campos[b], Livia de Figueiredo Diniz[e], Marisi Gomes Soares[a], Daniela Aparecida Chagas de Paula[a], Danielle Ferreira Dias[a]**

[a]*Instituto de Química, Universidade Federal de Alfenas, MG, Brazil*

[b]*Departamento de Análises Clínicas e Toxicológicas, Universidade Federal de Santa Maria, RS, Brazil*

[c]*Escola de Farmácia, Universidade Federal de Ouro Preto, MG, Brazil*

[d]*Faculdade de Ciências Farmacêuticas, Universidade Federal de Alfenas, MG, Brazil*

[e]*Instituto de Ciências Biomédicas, Universidade Federal de Alfenas, MG, Brazil*

Contents:

Infrared, ^{1}H and ^{13}C NMR, and HRMS spectra of compounds

Infrared, ^{1}H and ^{13}C NMR, and HRMS spectra of compounds

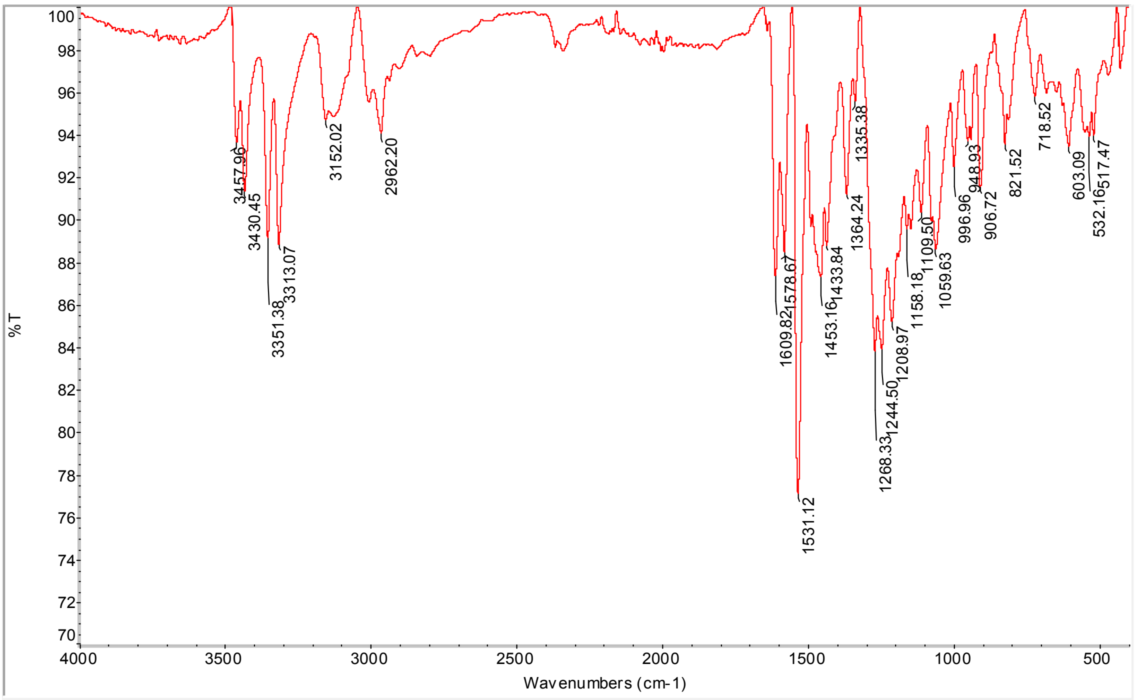

Figure S1 Infrared spectrum of compound **7**

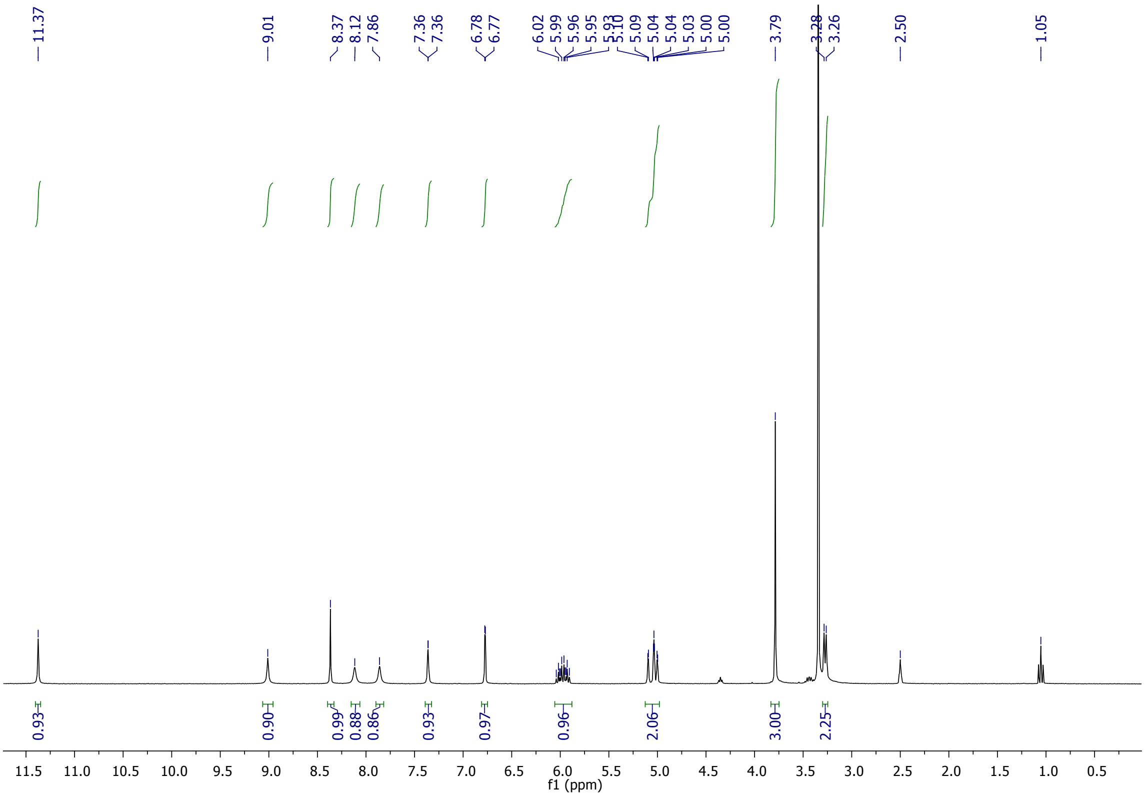

Figure S2 ^{1}H NMR spectrum of compound **7**

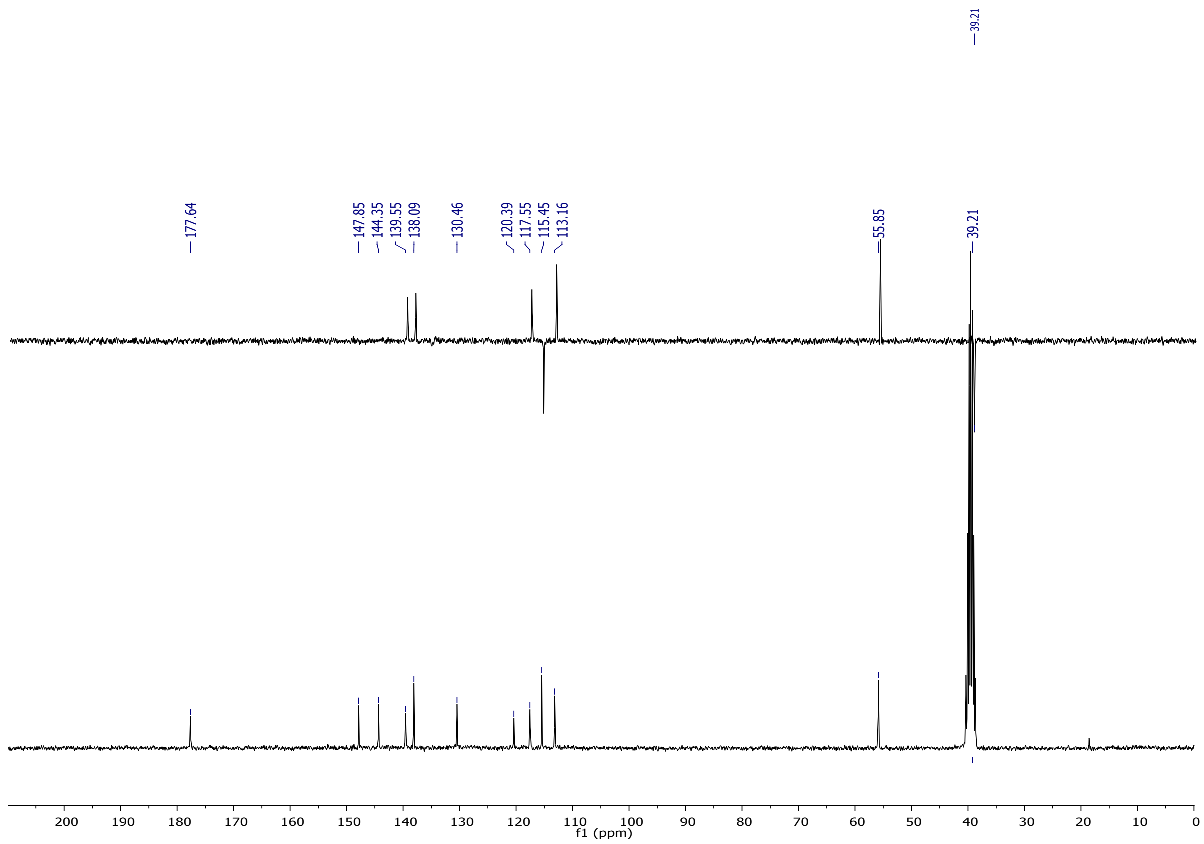

Figure S3 DEPT 135 and ^{13}C NMR spectrum of compound **7**

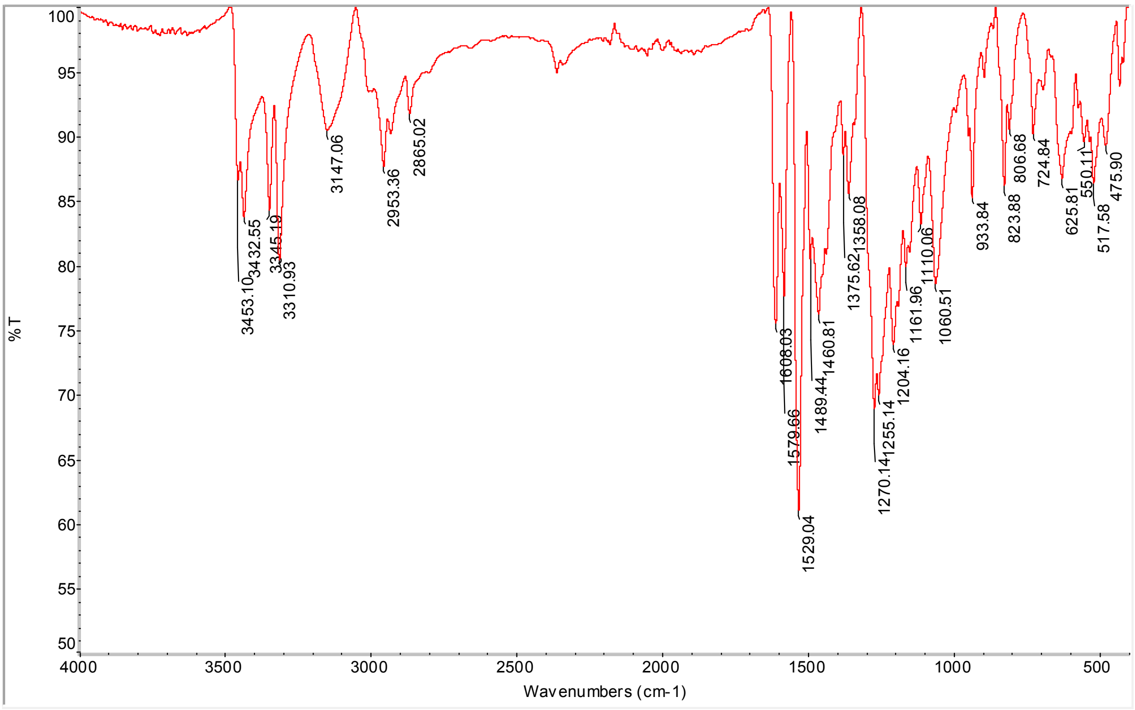

Figure S4 Infrared spectrum of compound **8**

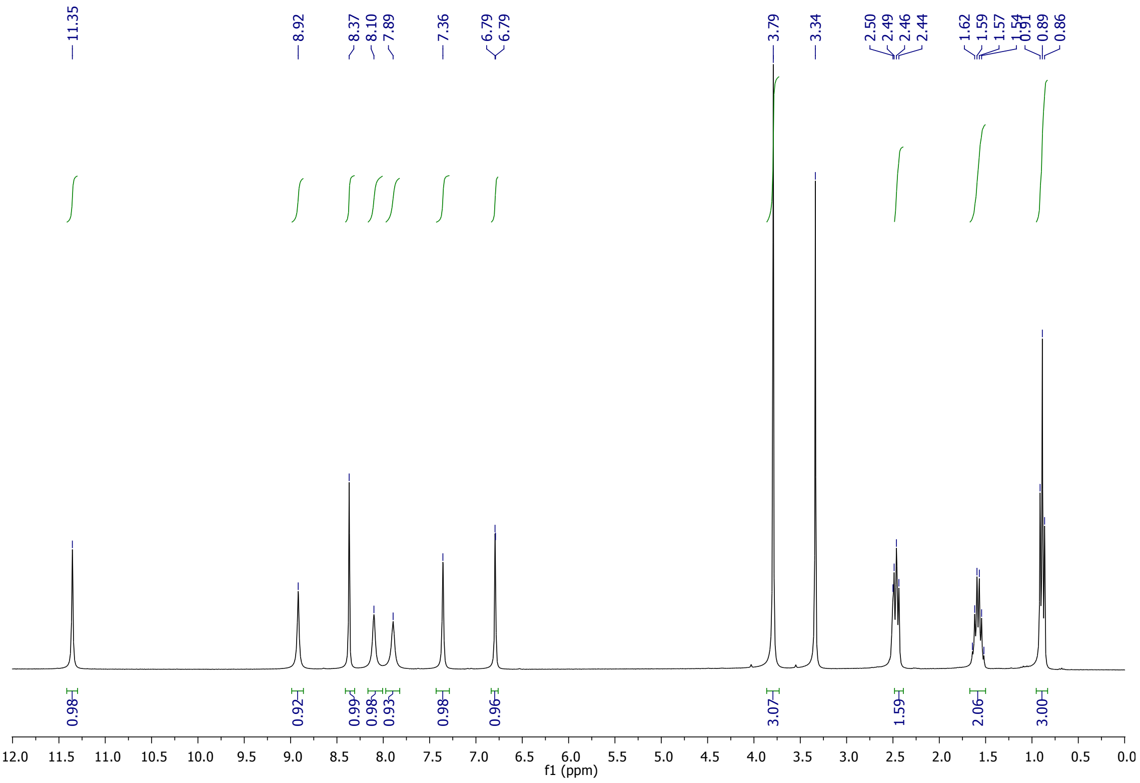

Figure S5 ^{1}H NMR spectrum of compound **8**

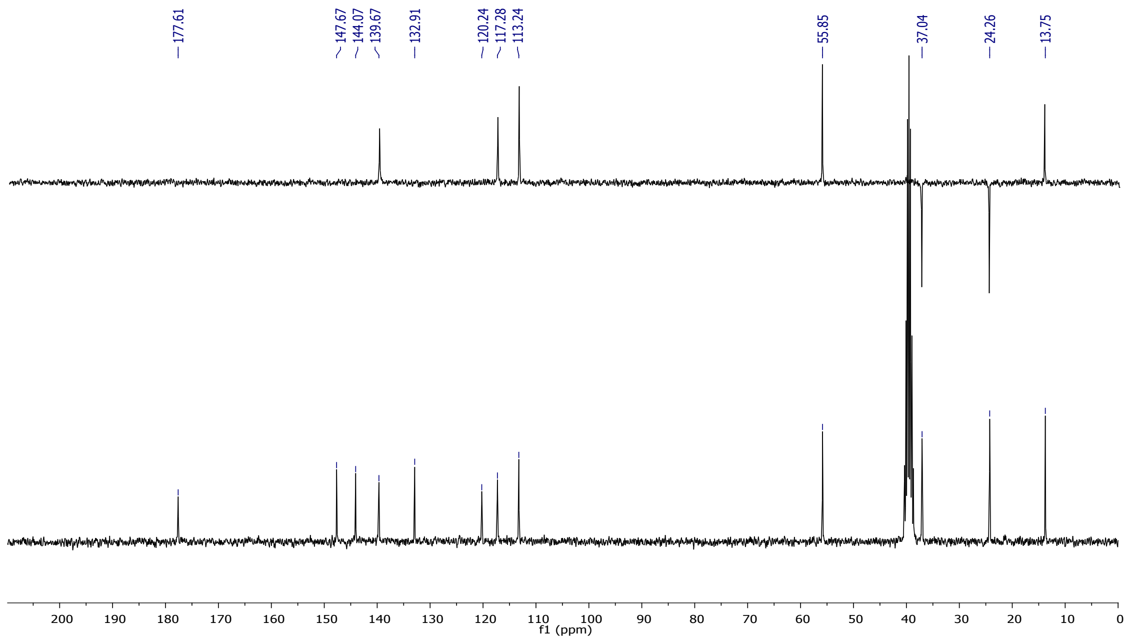

Figure S6 DEPT 135 and ^{13}C NMR spectrum of compound **8**

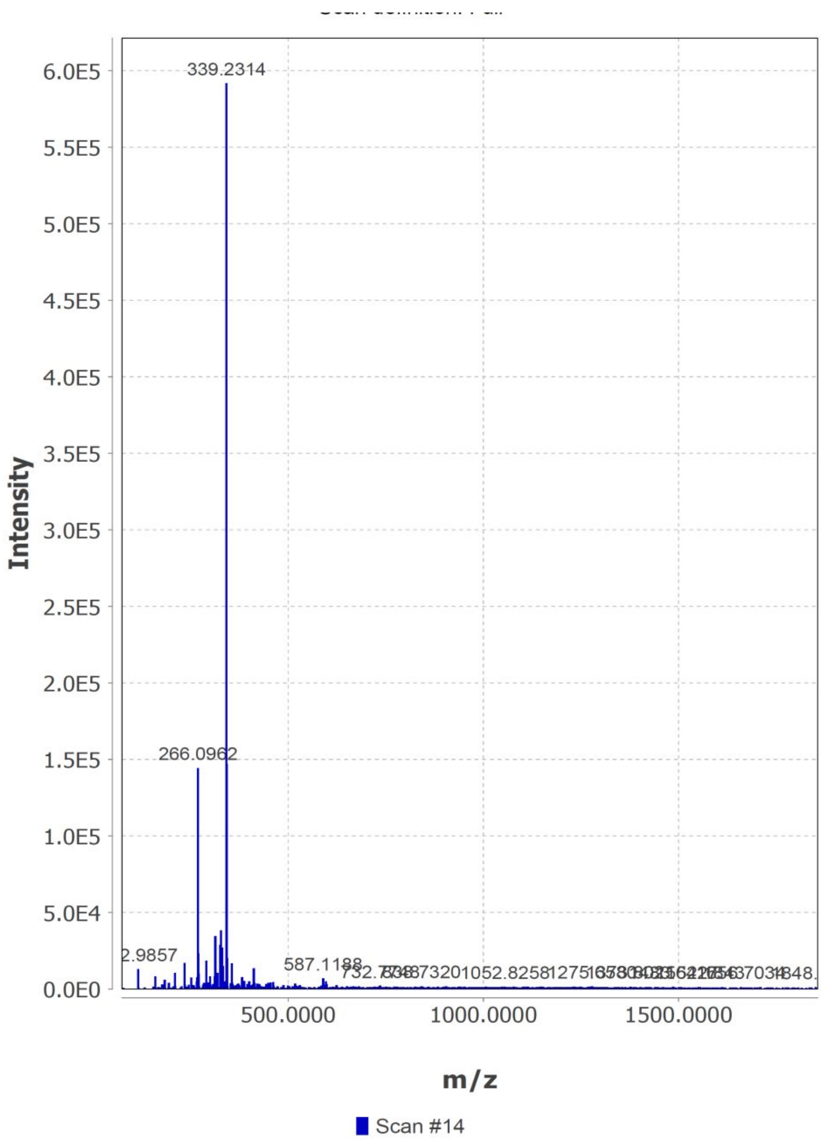

Figure S7 HRM spectrum of compound **8**

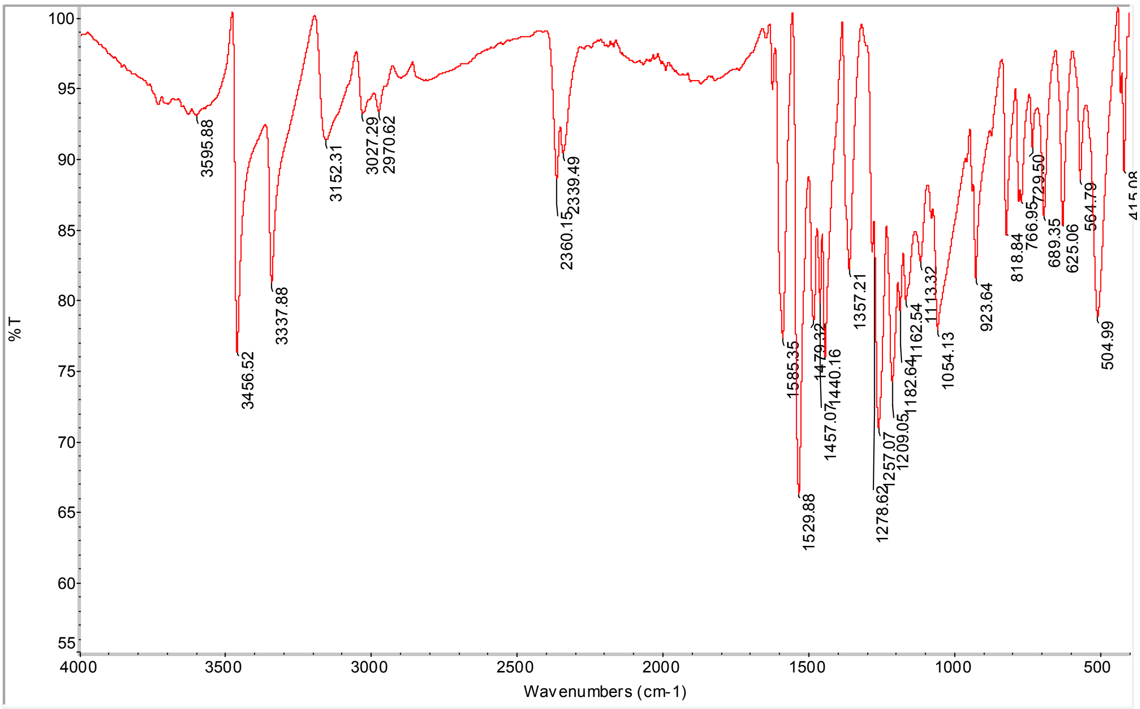

Figure S8 Infrared spectrum of compound **9**

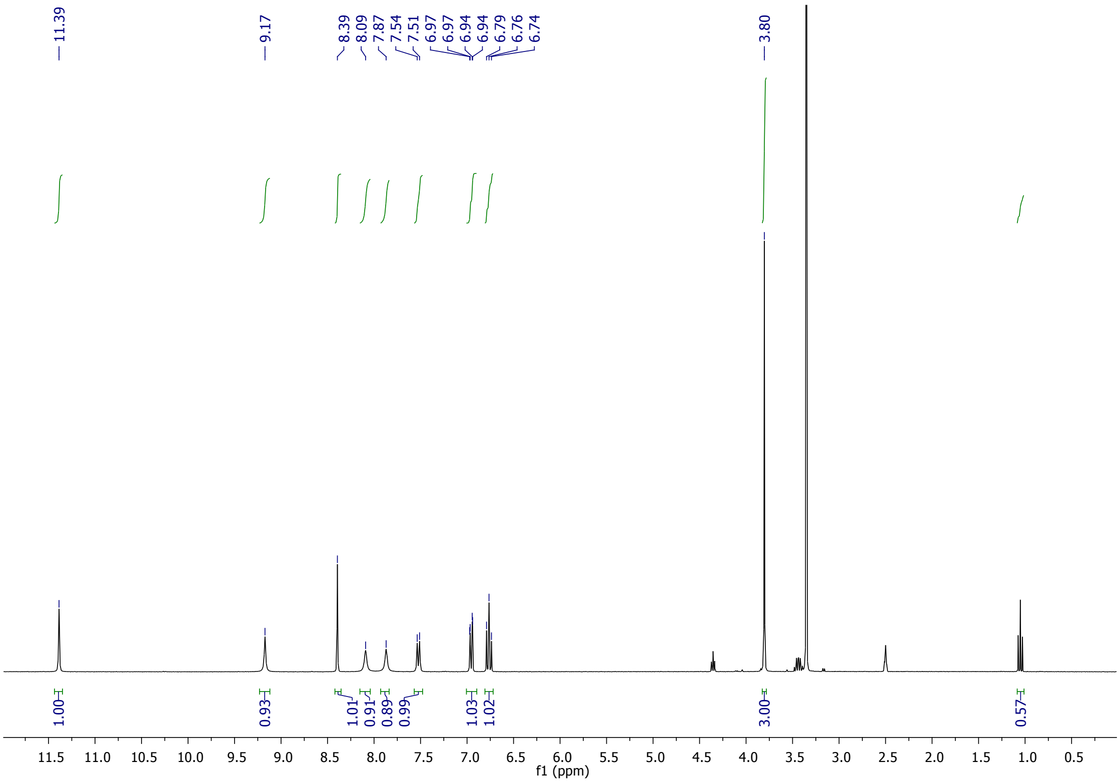

Figure S9 ^{1}H NMR spectrum of compound **9**

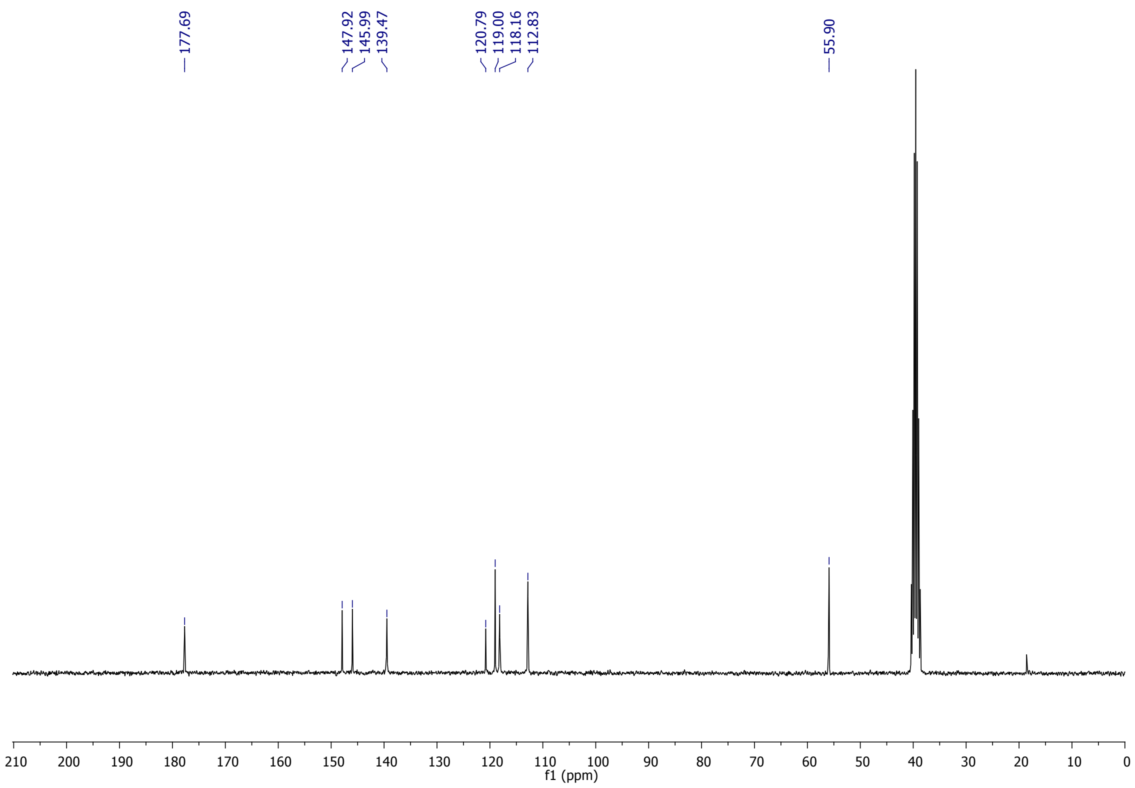

Figure S10 ^{13}C NMR spectrum of compound **9**

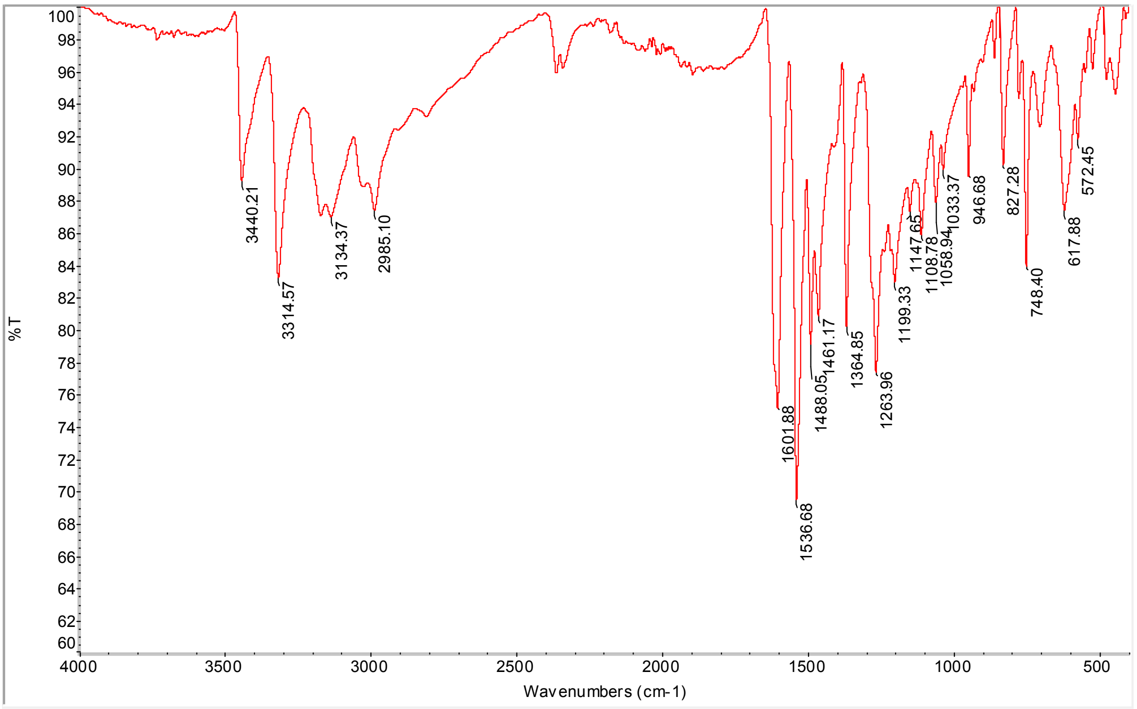

Figure S11 Infrared spectrum of compound **10**

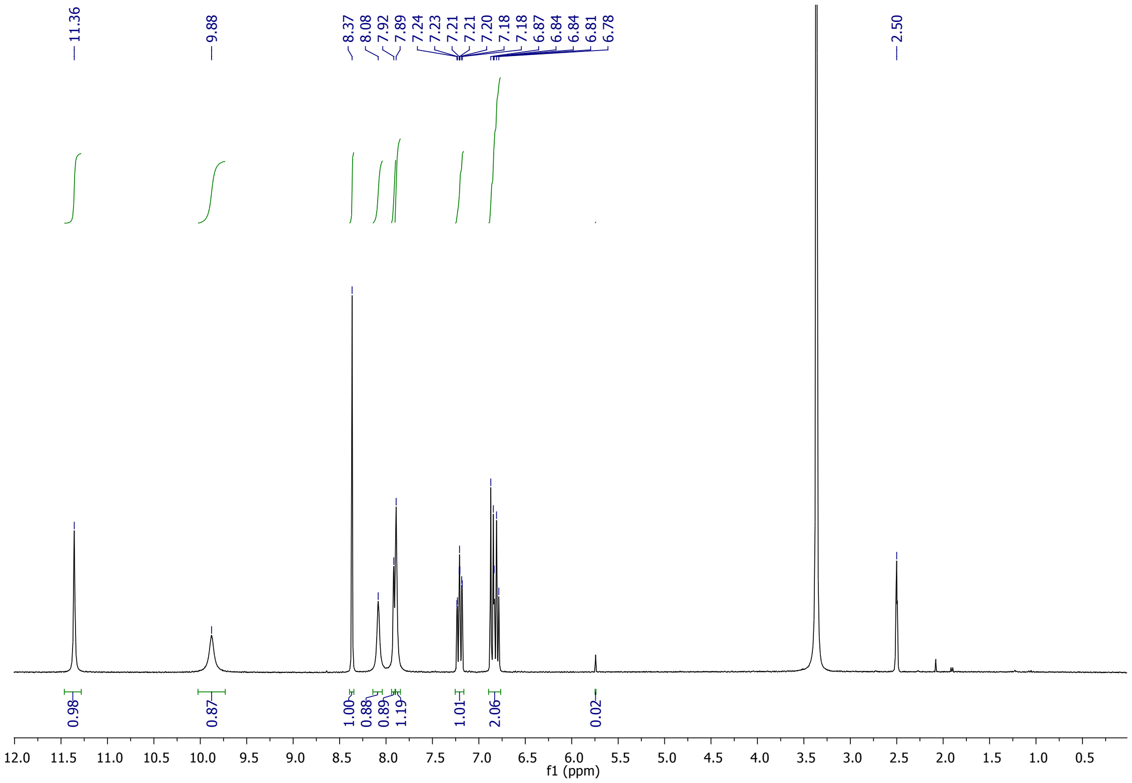

Figure S12 ^{1}H NMR spectrum of compound **10**

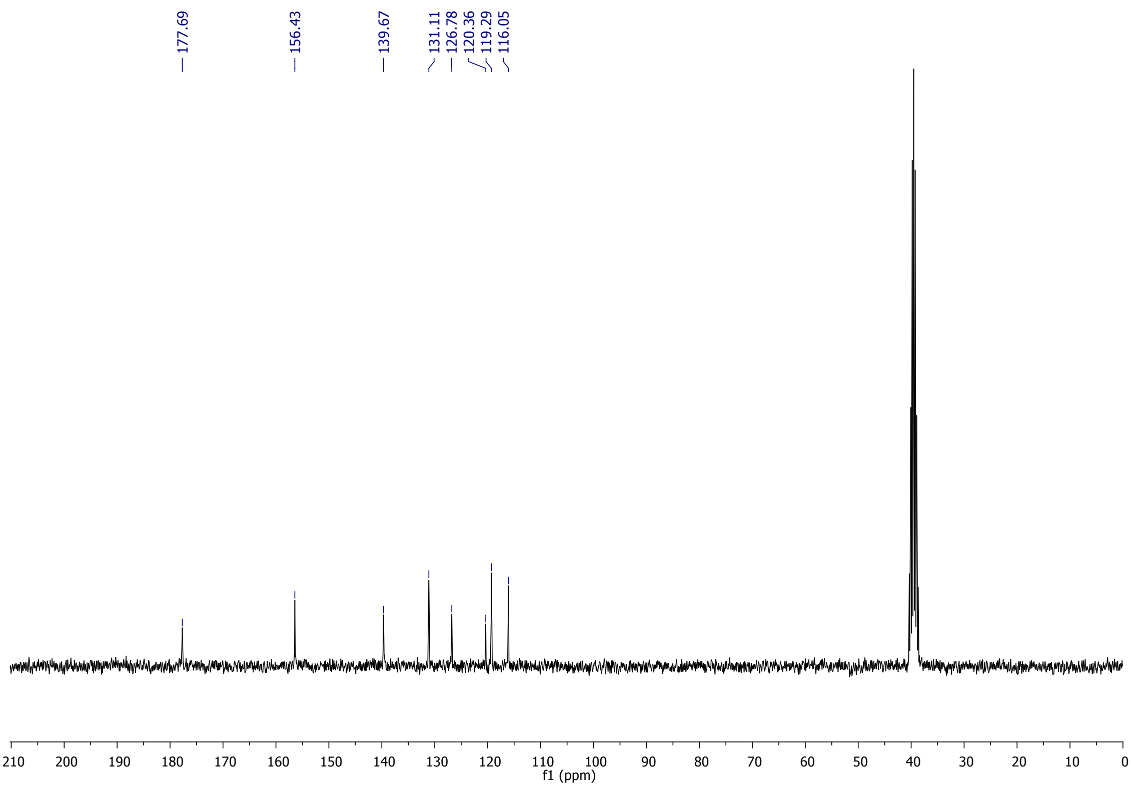

Figure S13 ^{13}C NMR spectrum of compound **10**

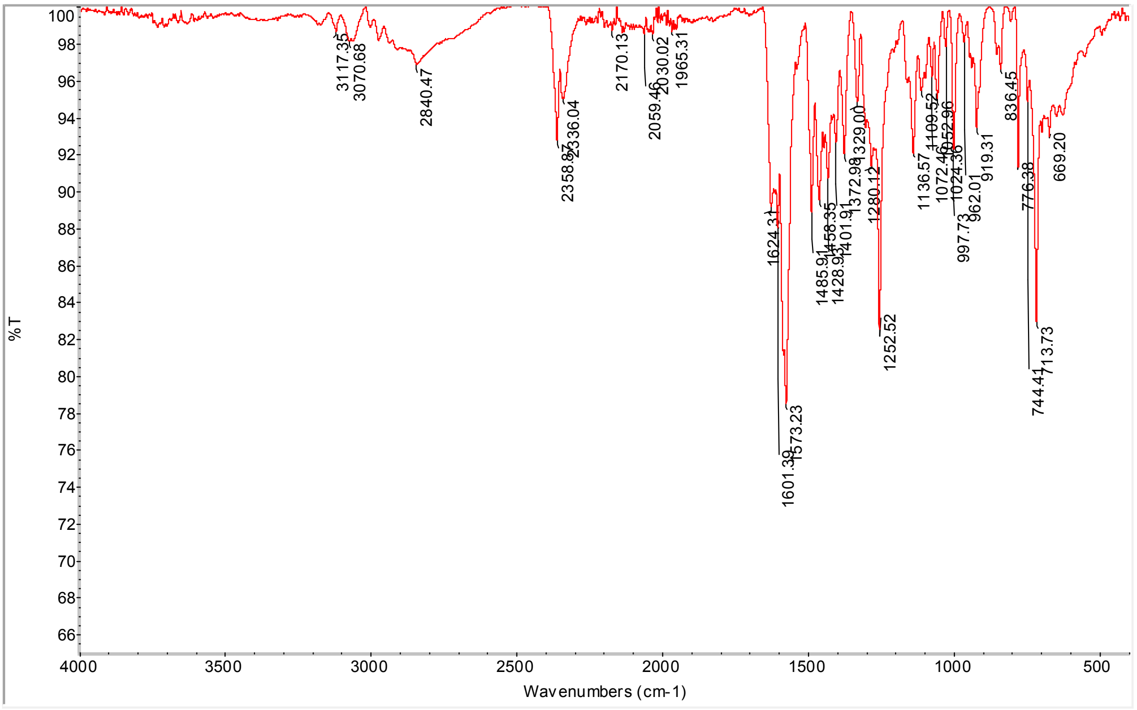

Figure S14 Infrared spectrum of compound **11**

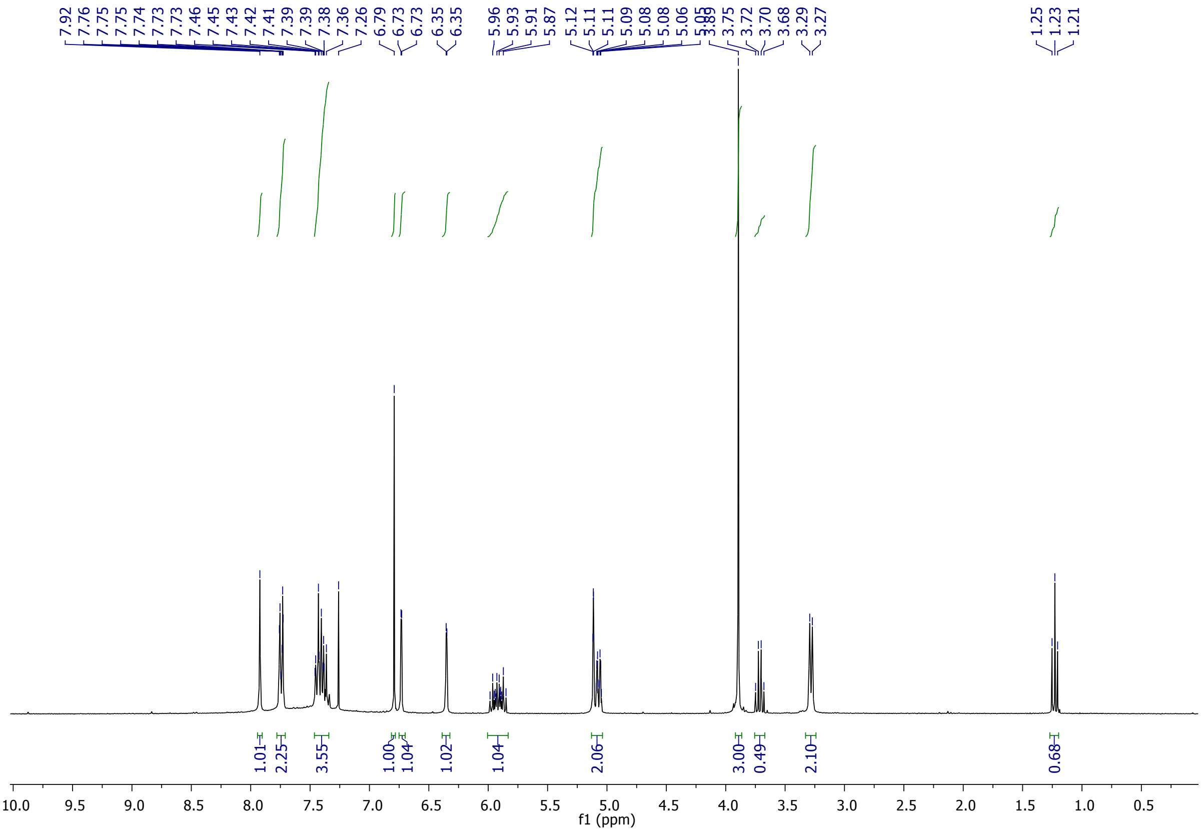

Figure S15 ^{1}H NMR spectrum of compound **11**

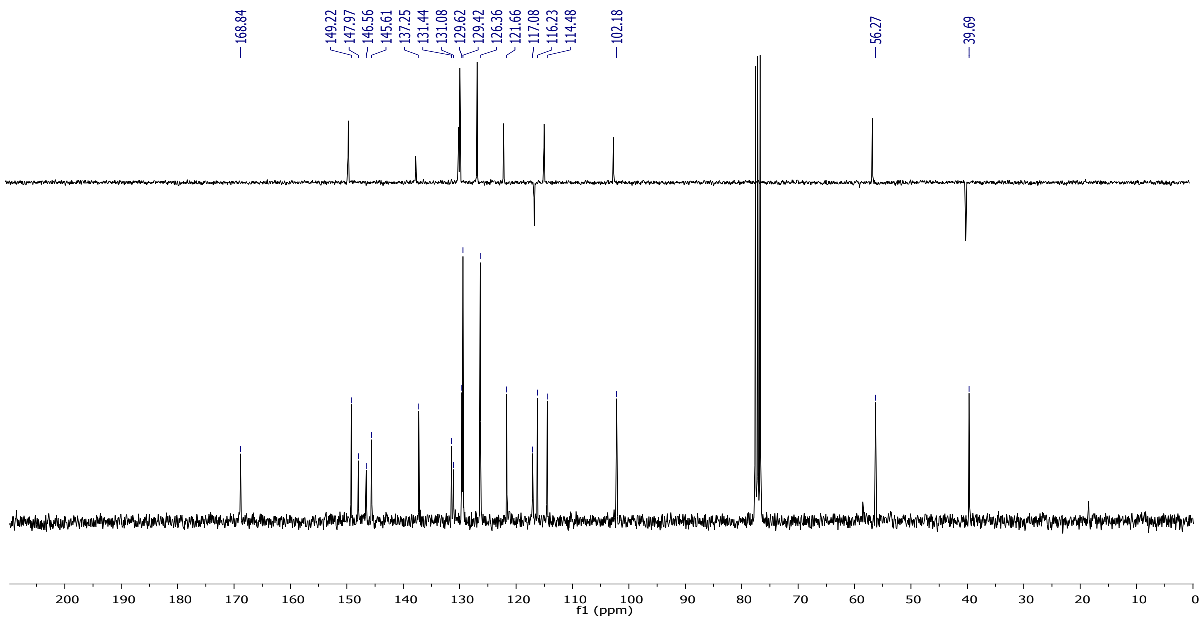

Figure S16 DEPT-135 and ^{13}C NMR spectrum of compound **11**

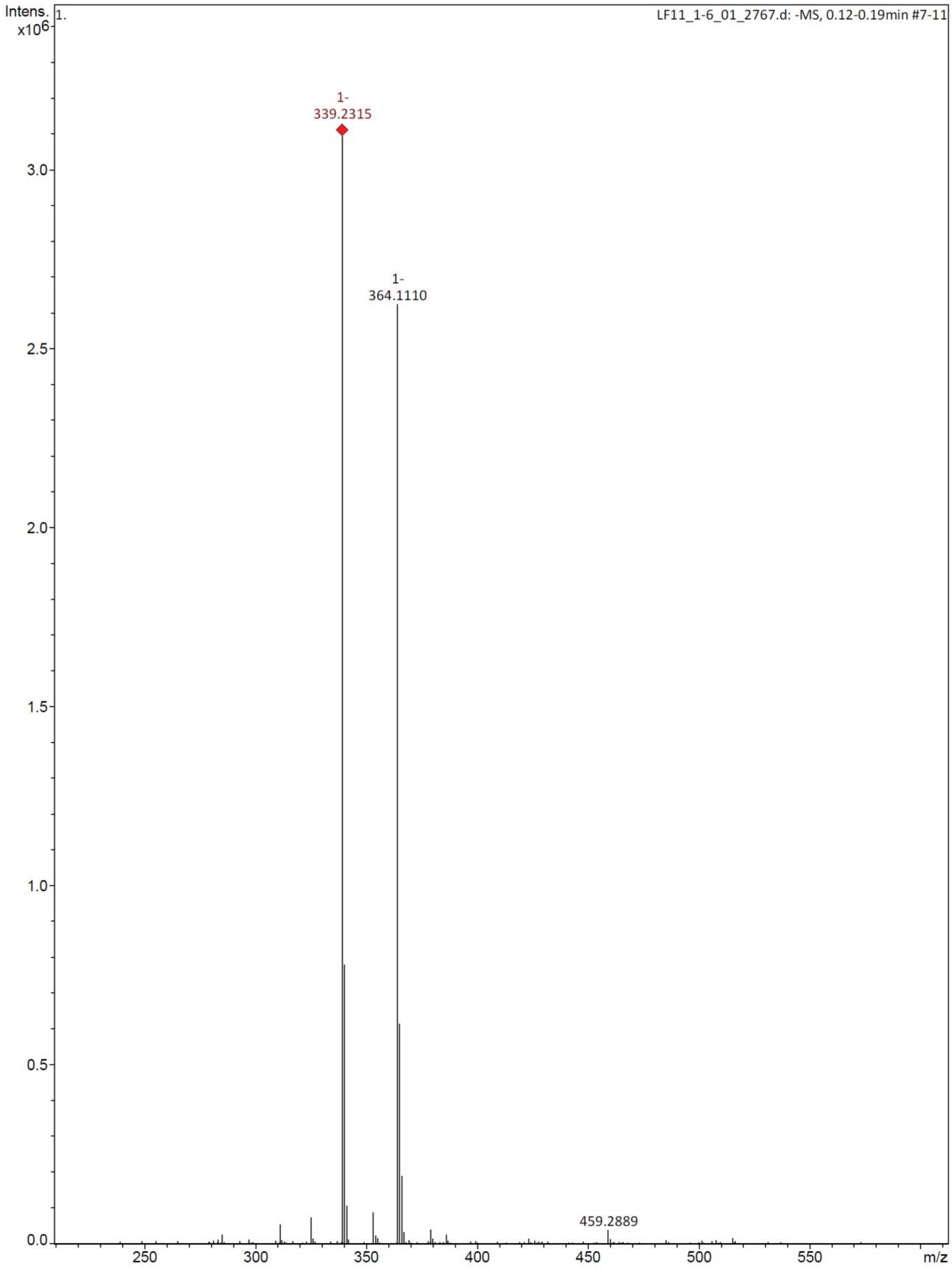

Figure S17 HRM spectrum of compound **11**

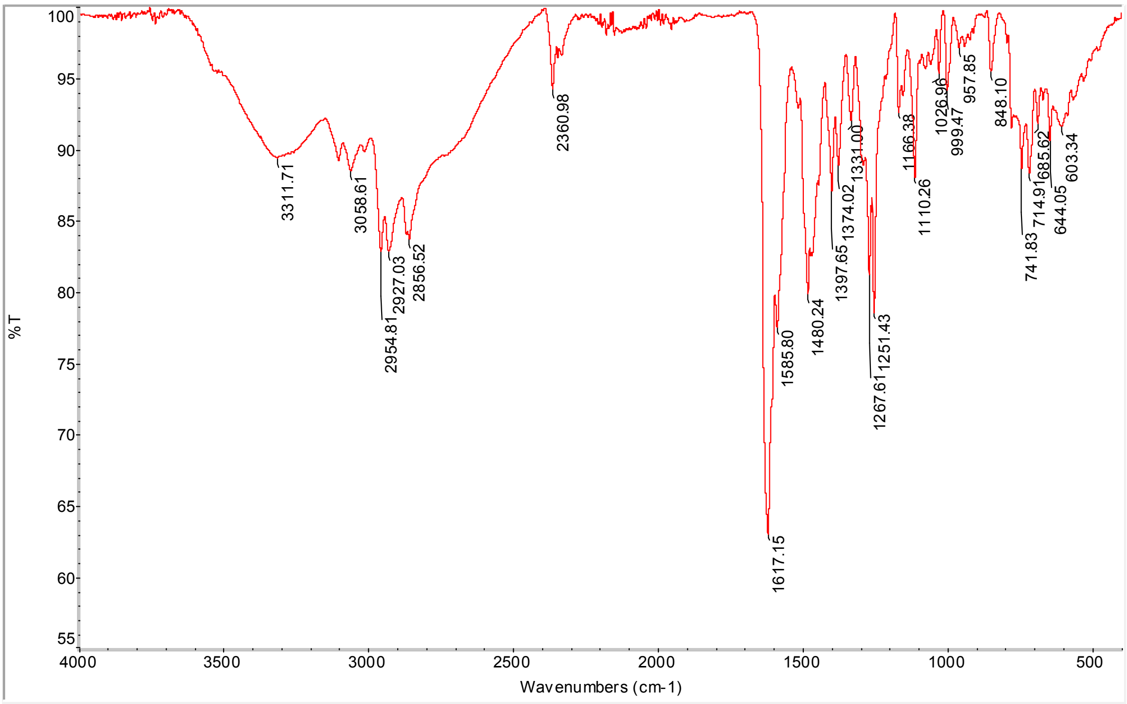

Figure S18 Infrared spectrum of compound **12**

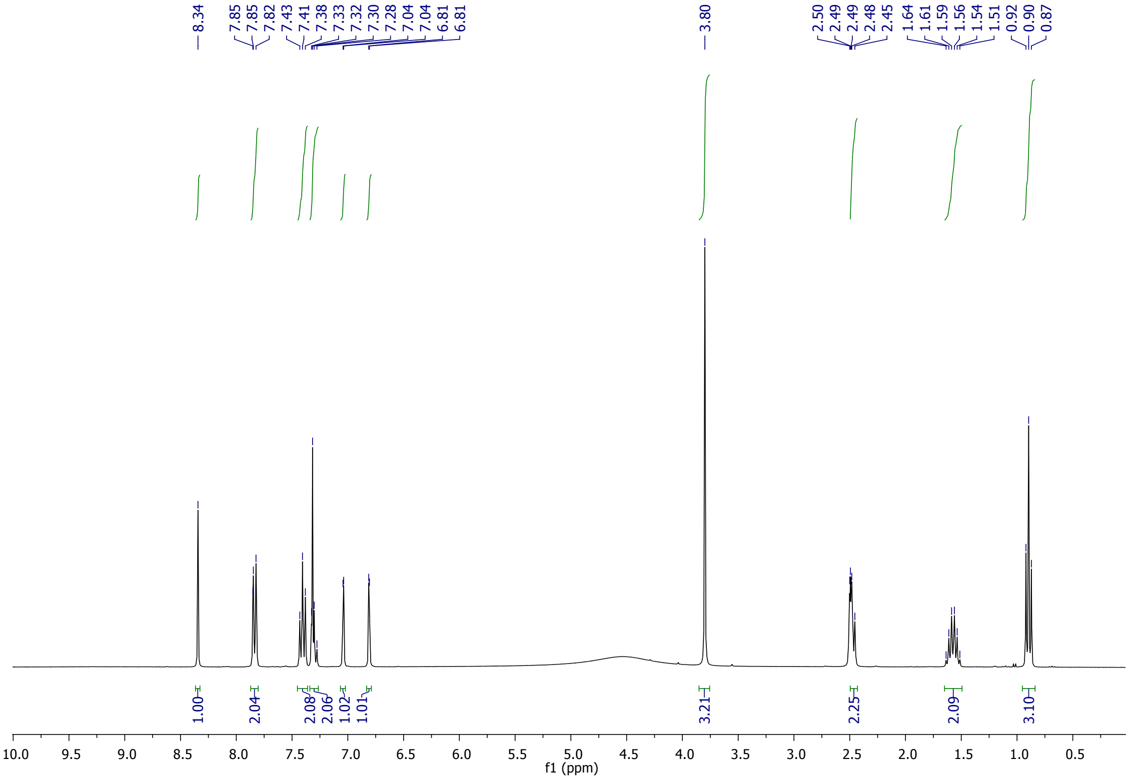

Figure S19 ^{1}H NMR spectrum of compound **12**

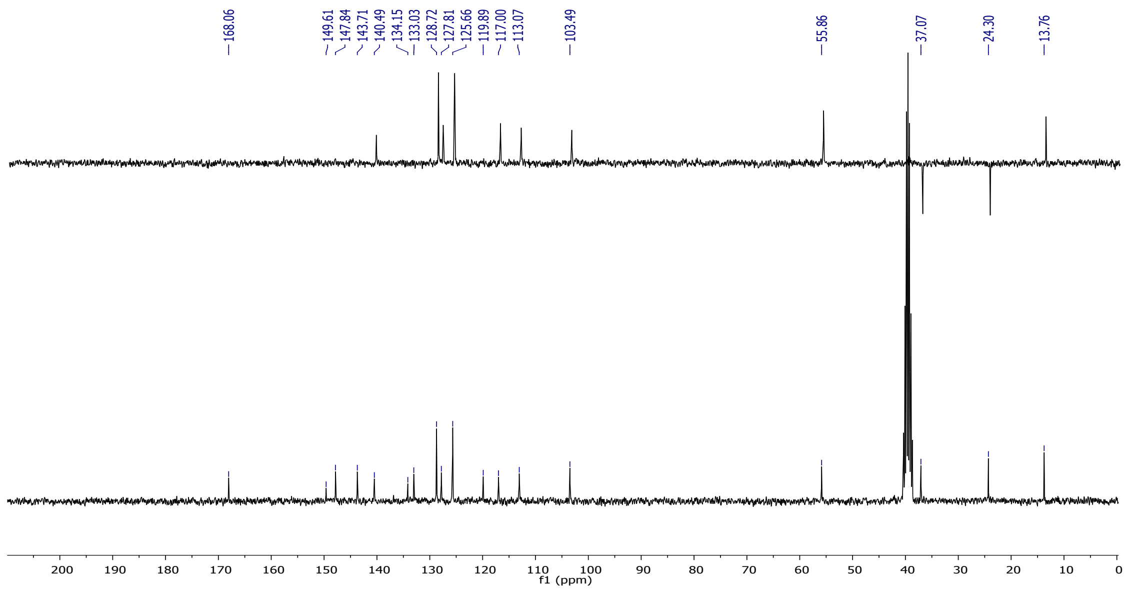

Figure S20 DEPT-135 and ^{13}C NMR spectrum of compound **12**

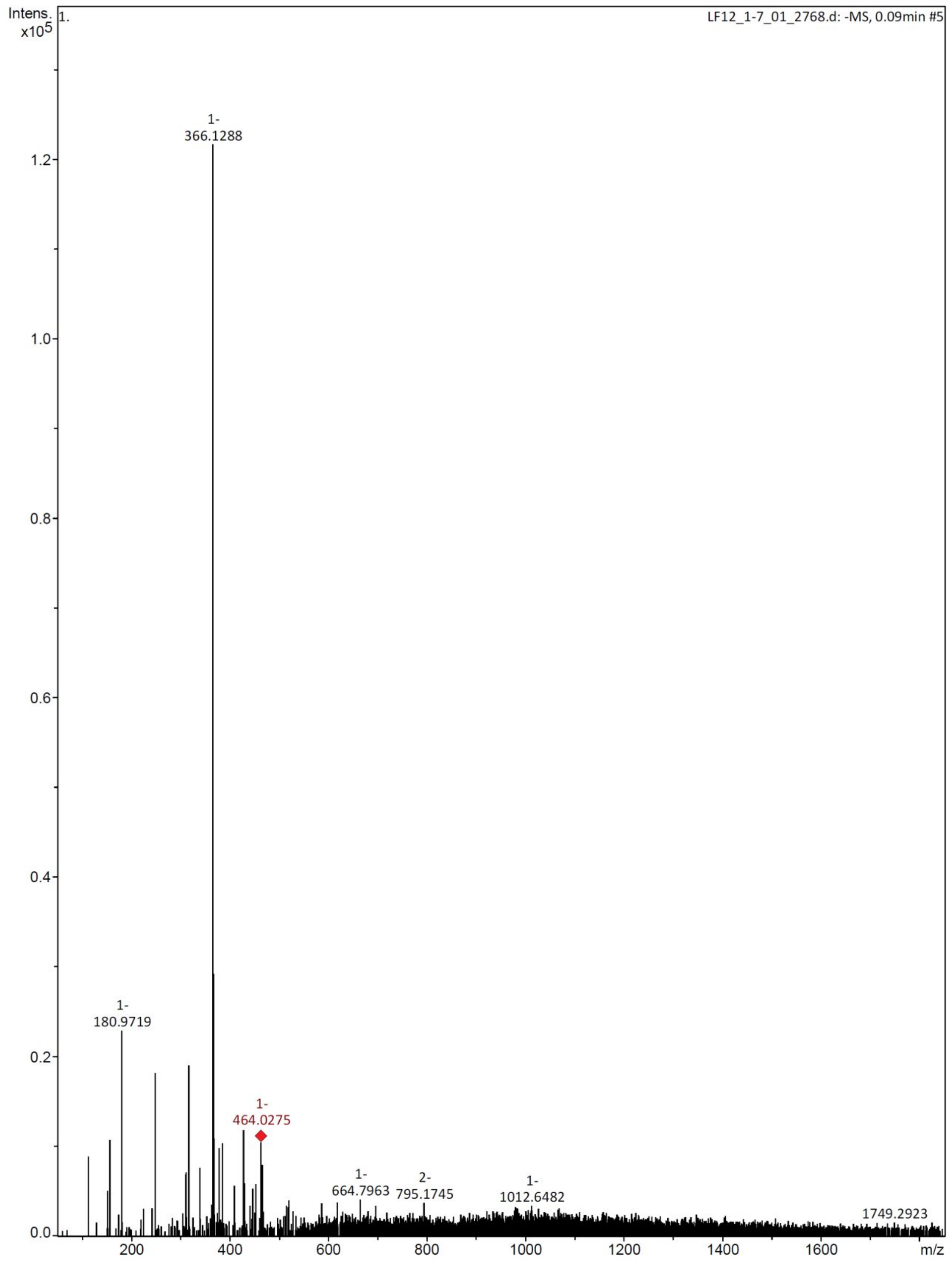

Figure S21 HRM spectrum of compound **12**

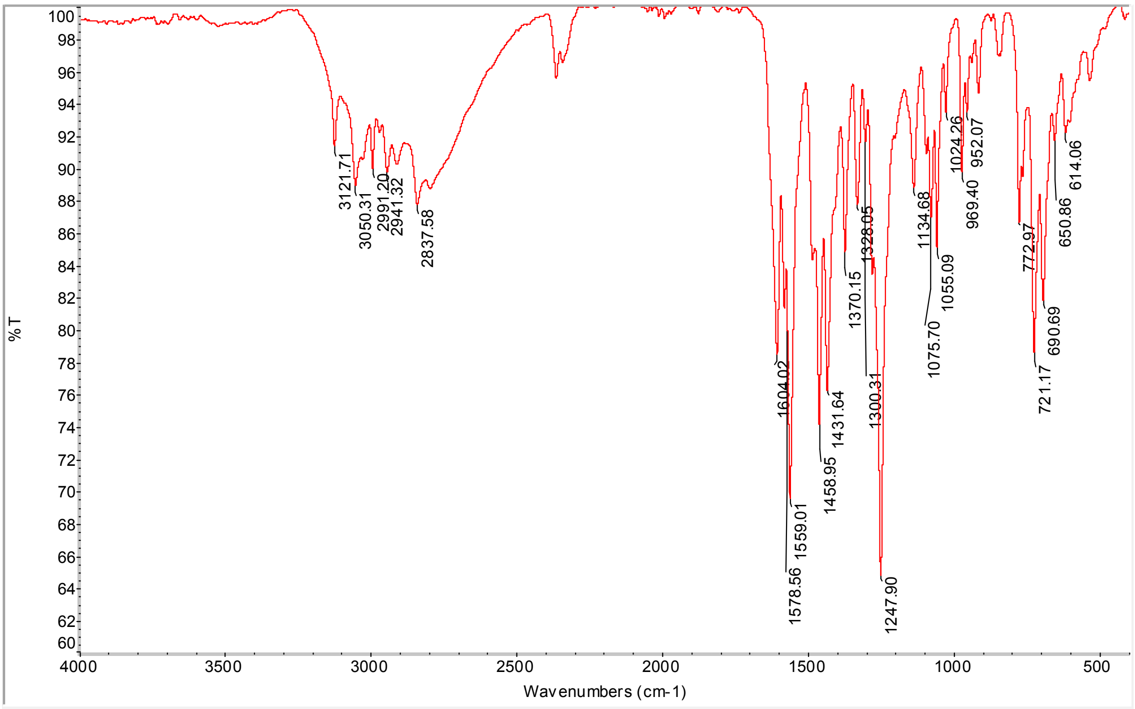

Figure S22 Infrared spectrum of compound **13**

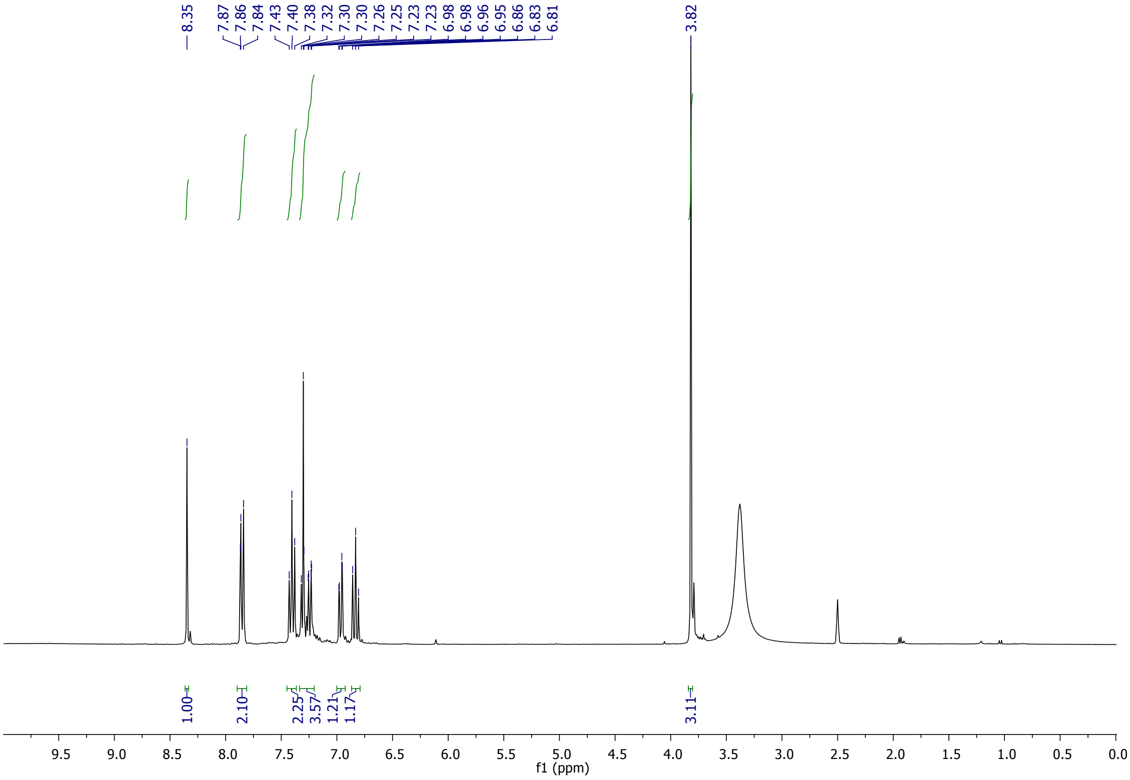

Figure S23 ^{1}H NMR spectrum of compound **13**

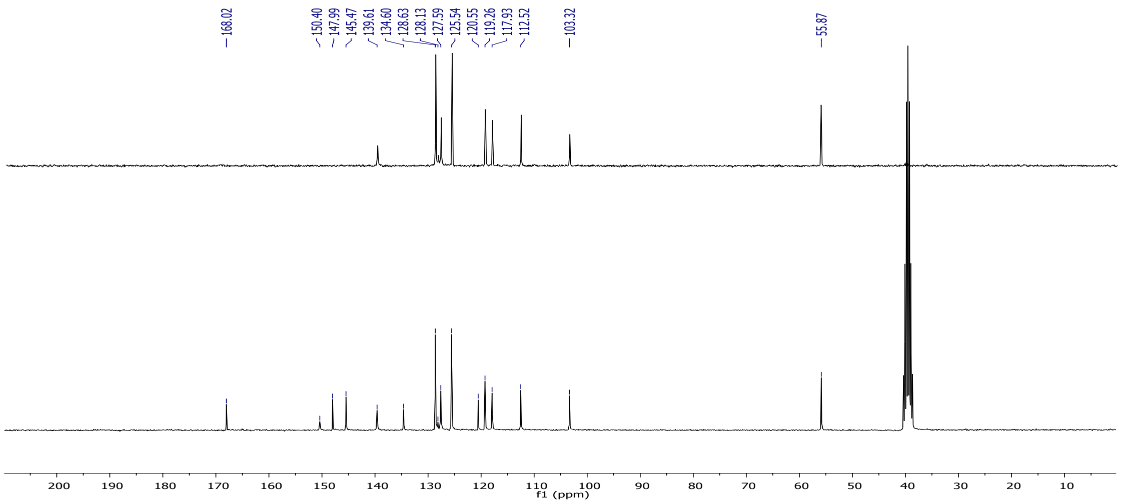

Figure S24 DEPT-135 and ^{13}C NMR spectrum of **13**

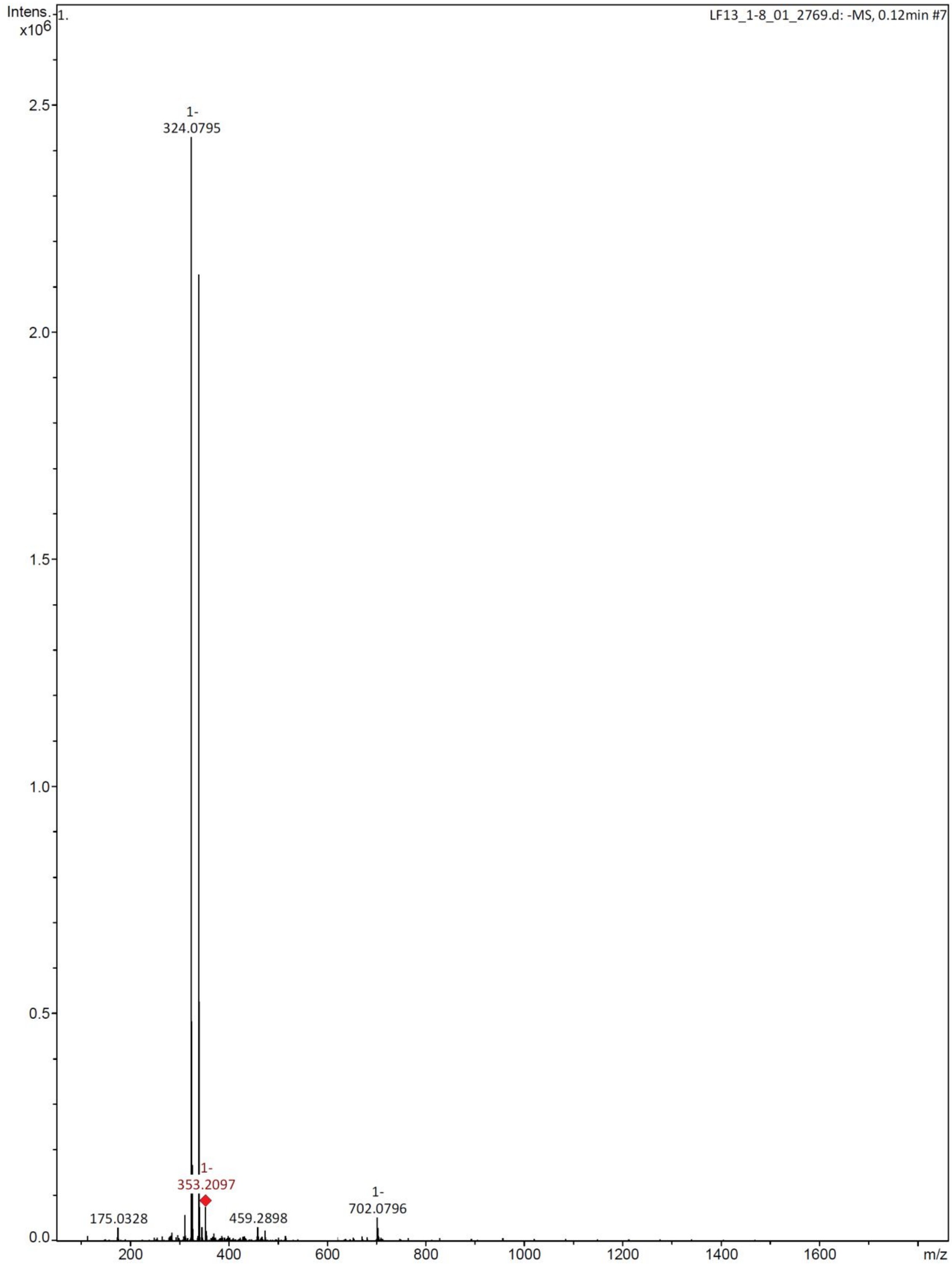

Figure S25 HRM spectrum of compound **13**

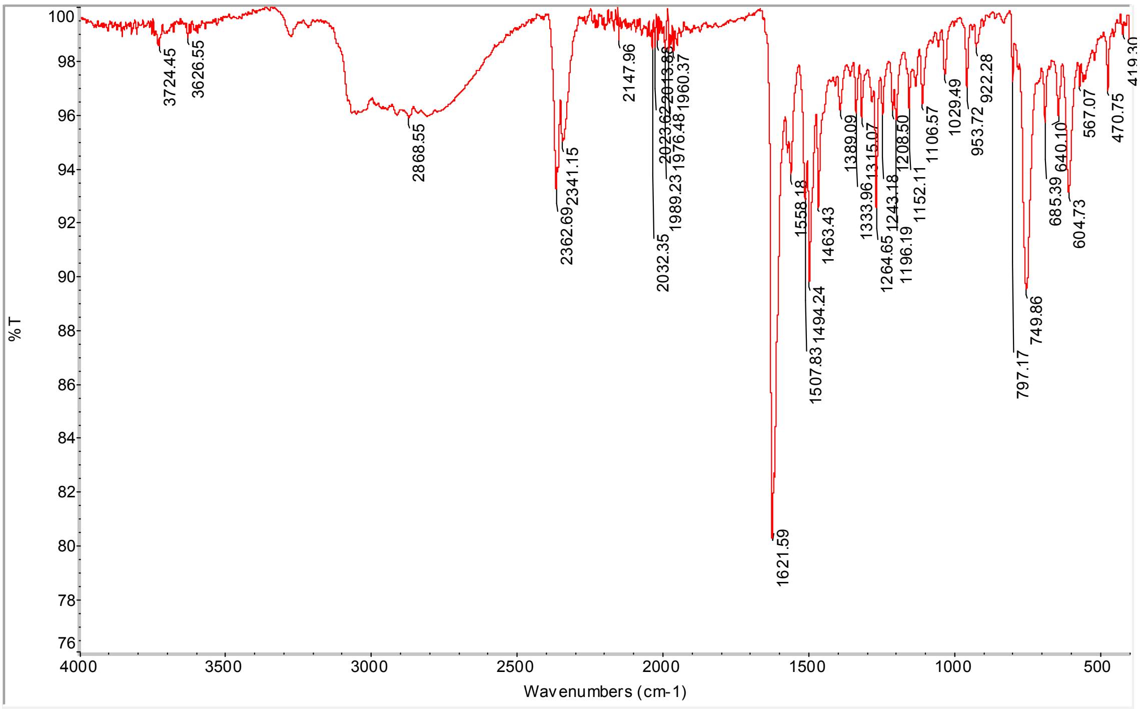

Figure S26 Infrared spectrum of compound **14**

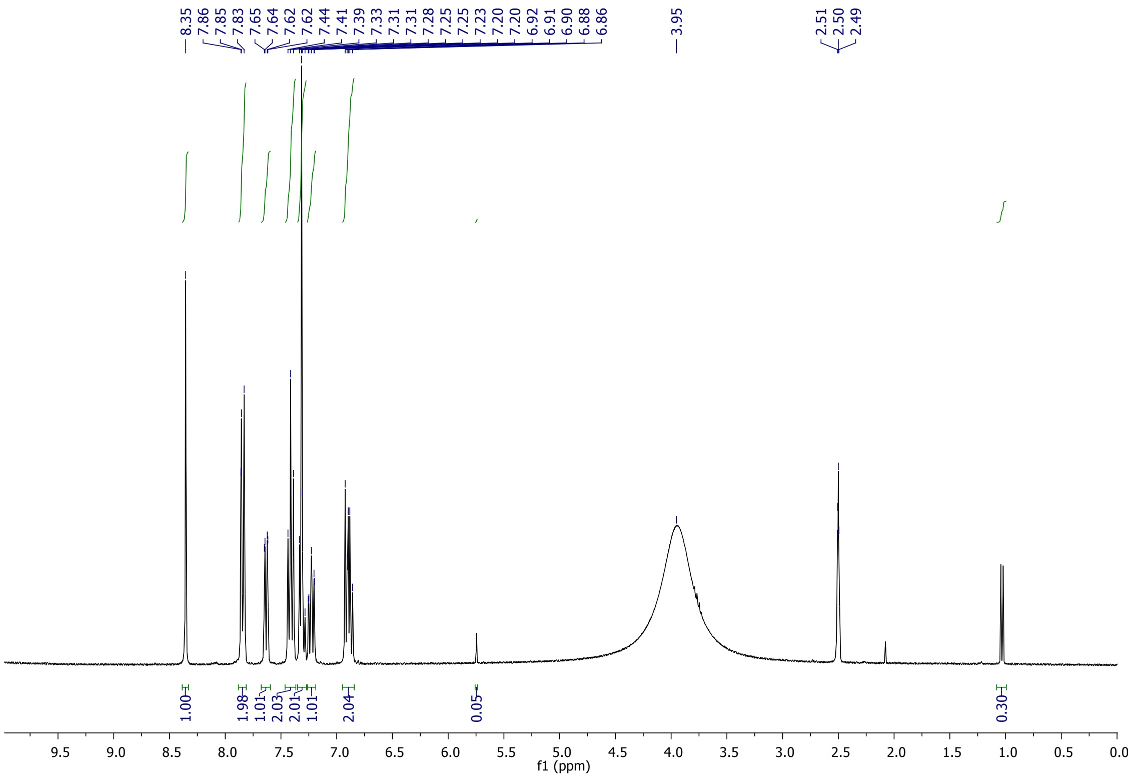

Figure S27 ^{1}H NMR spectrum of compound **14**

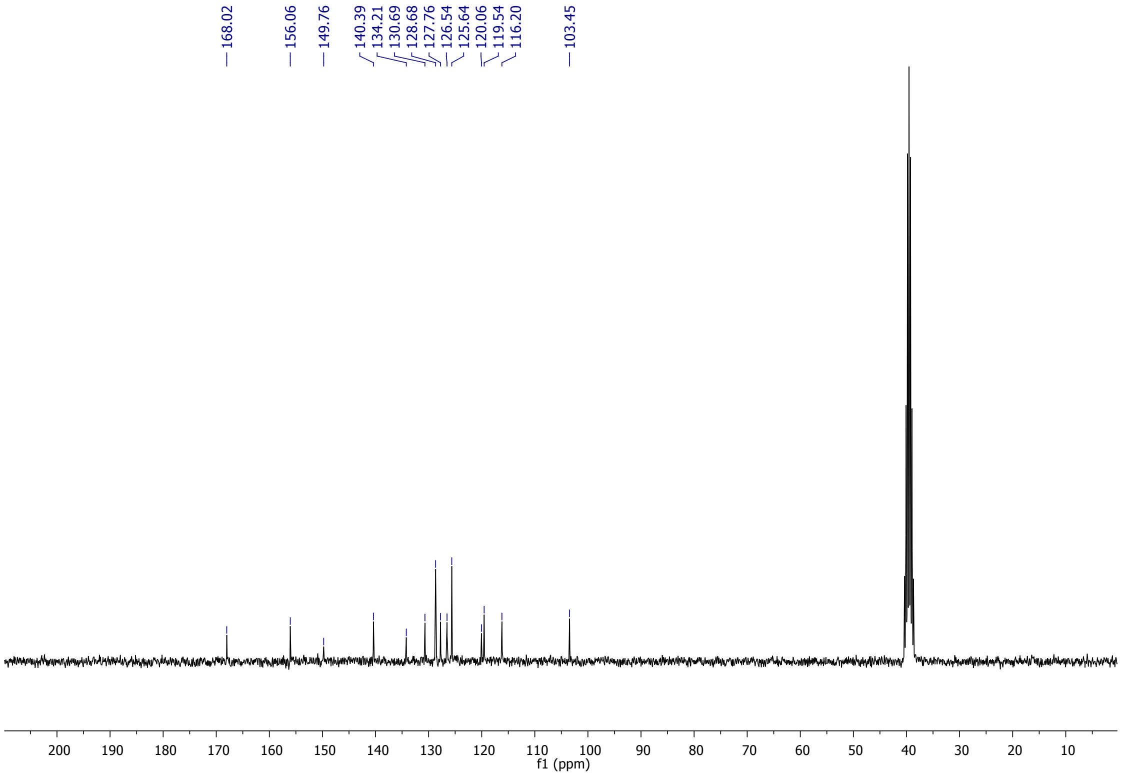

Figure S28 ^{13}C NMR spectrum of compound **14**

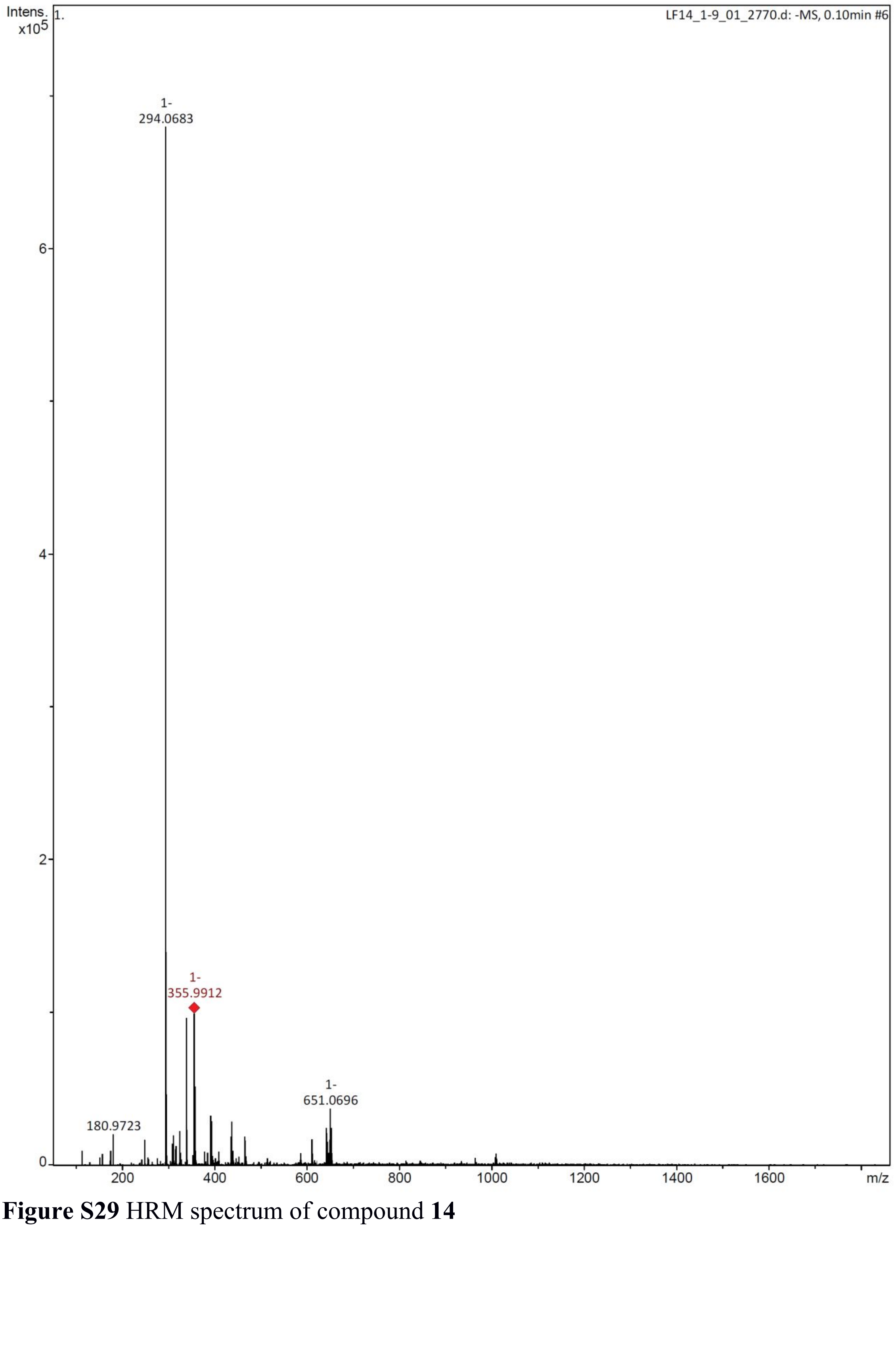

Figure S29 HRM spectrum of compound **14**

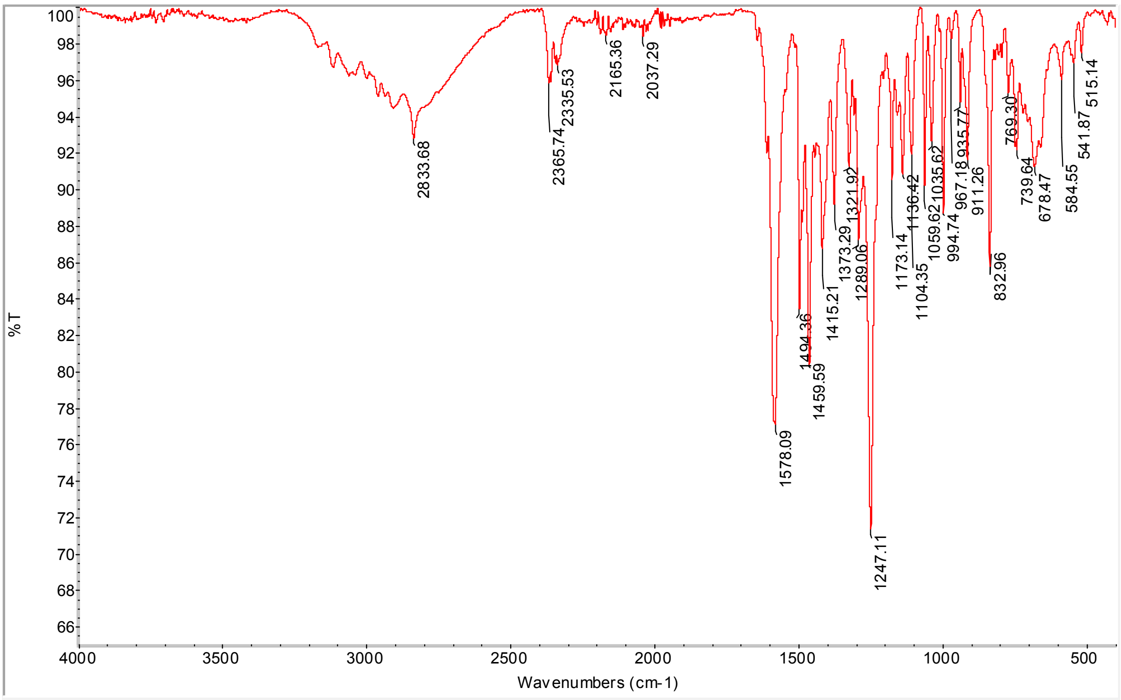

Figure S30 Infrared spectrum of compound **15**

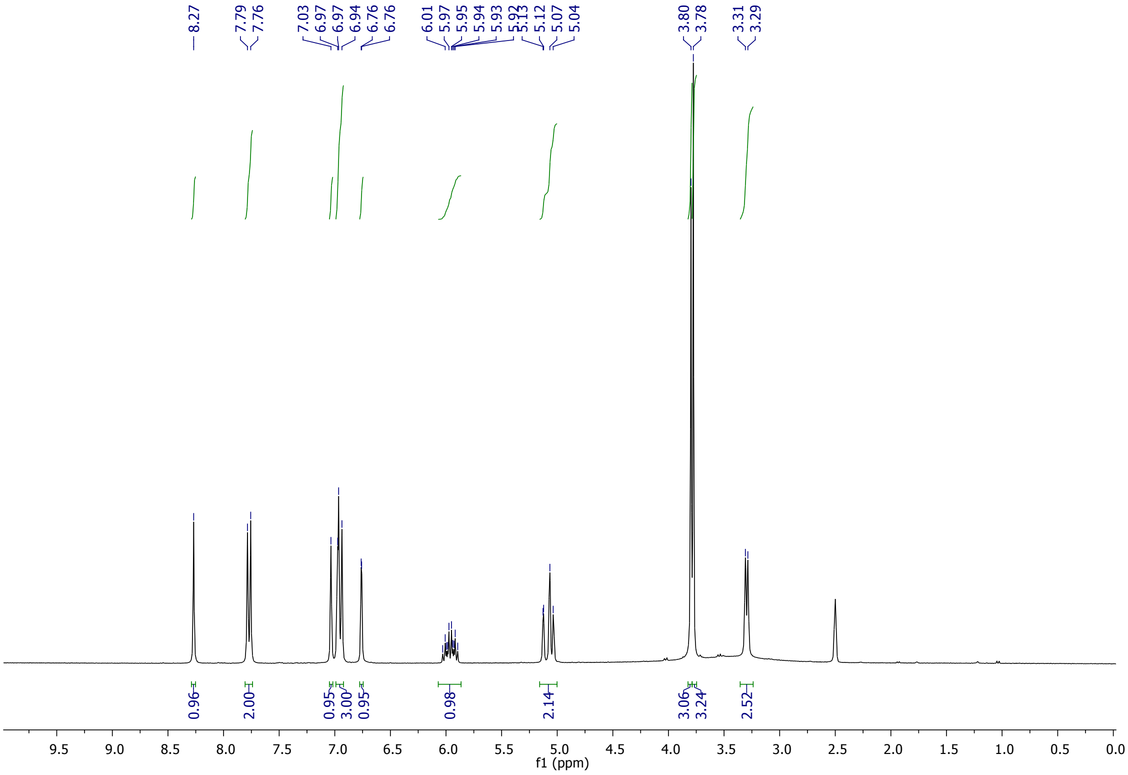

Figure S31 ^{1}H NMR spectrum of compound **15**

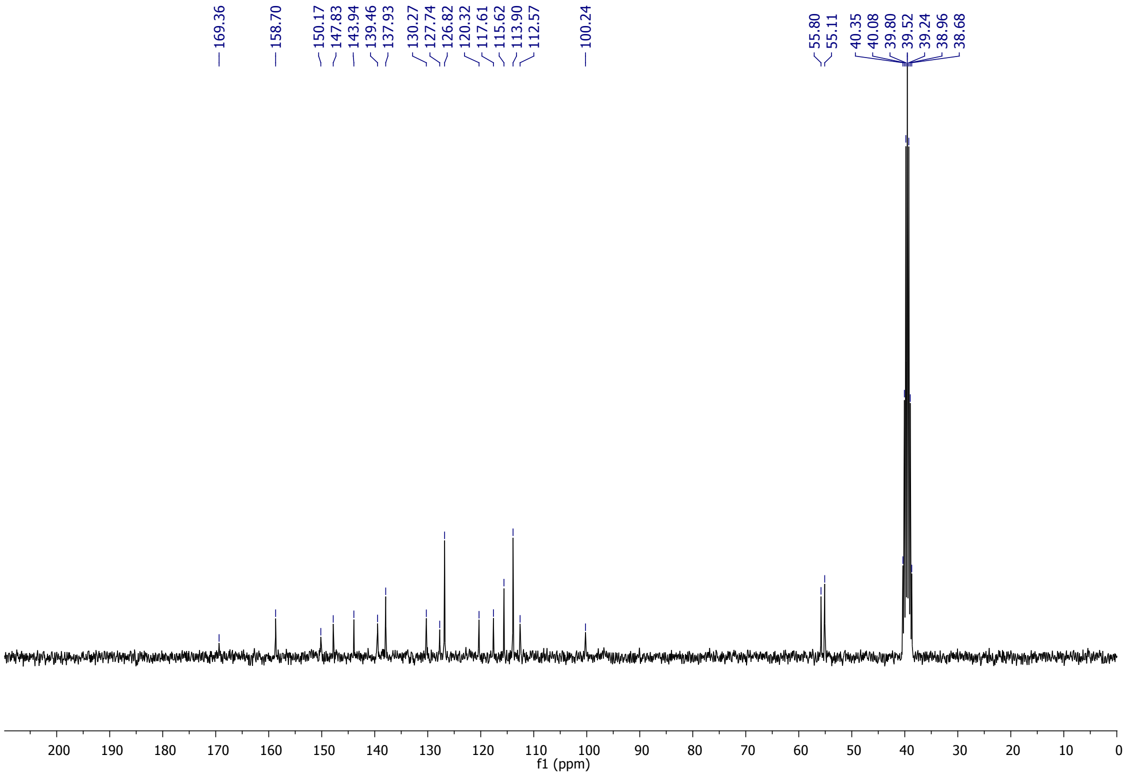

Figure S32 ^{13}C NMR spectrum of compound **15**

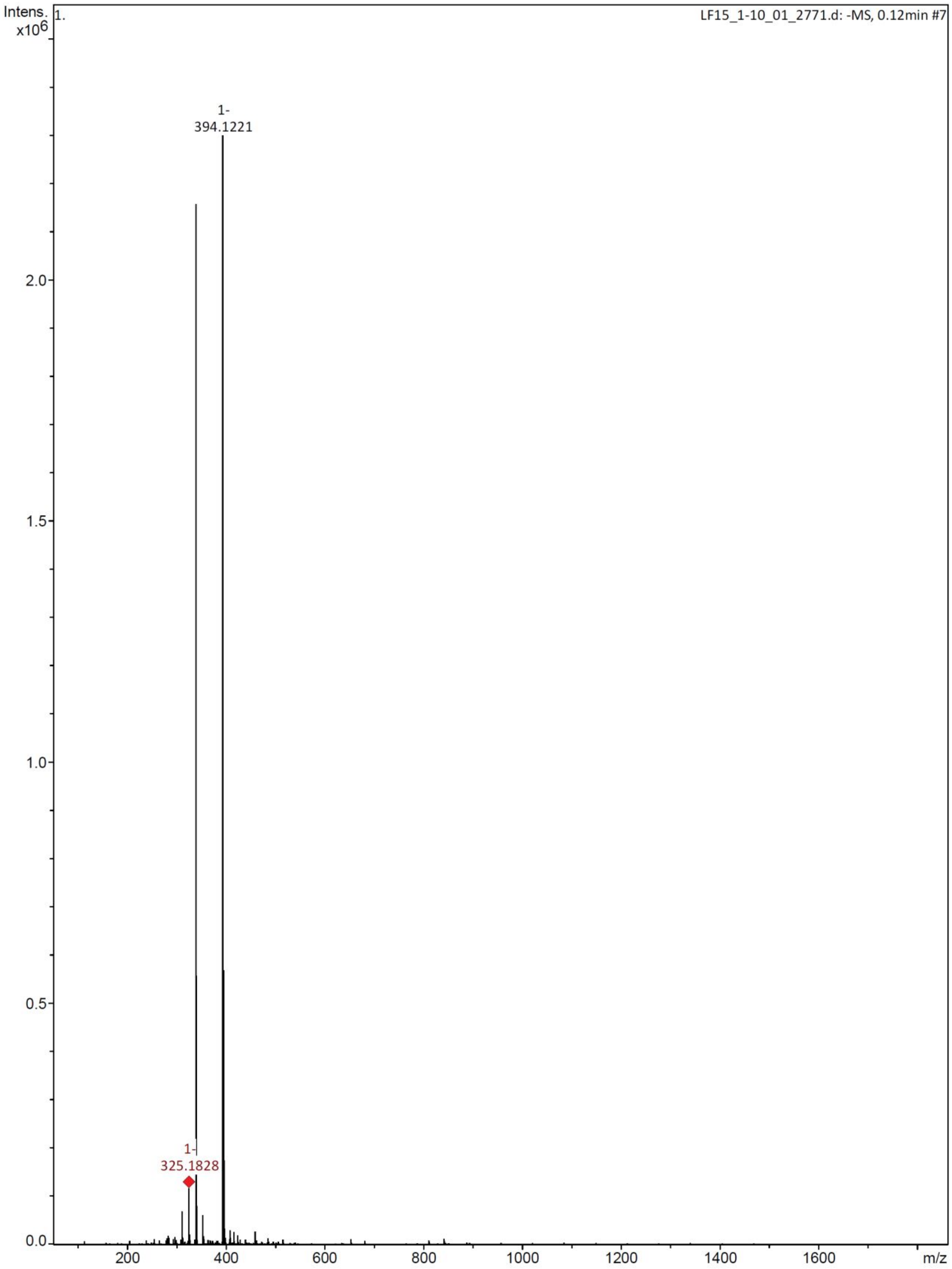

Figure S33 HRM spectrum of compound **15**

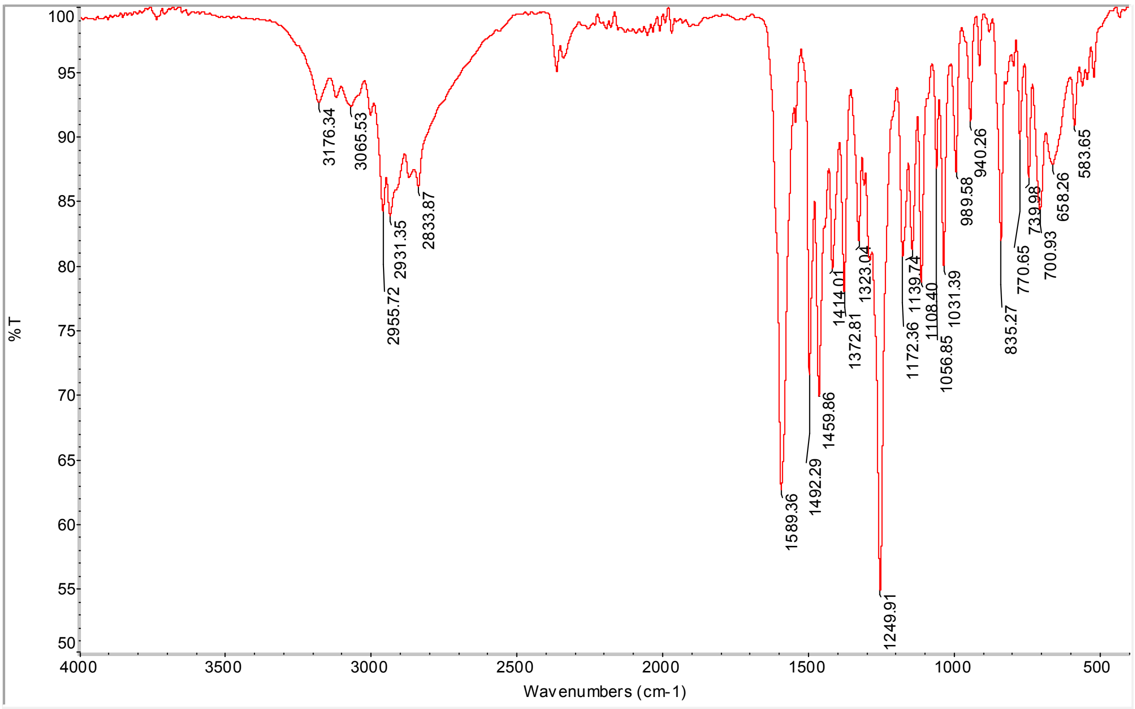

Figure S34 Infrared spectrum of compound **16**

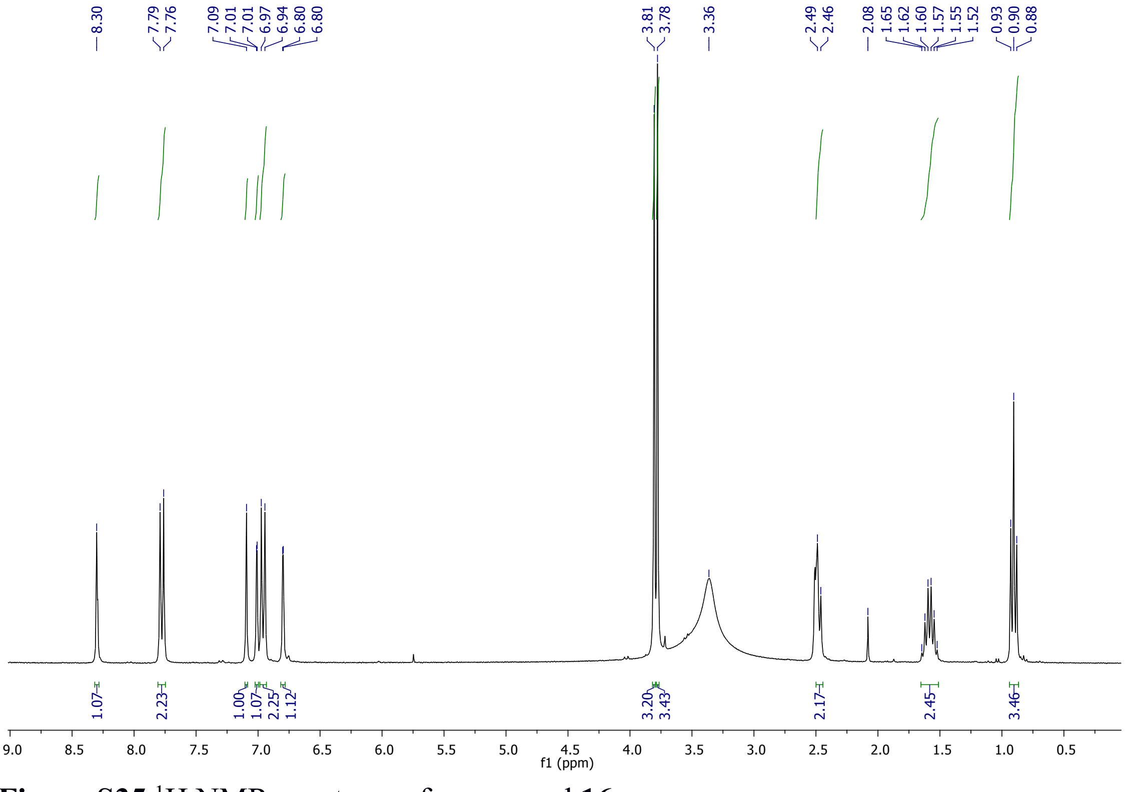

Figure S35 ^{1}H NMR spectrum of compound **16**

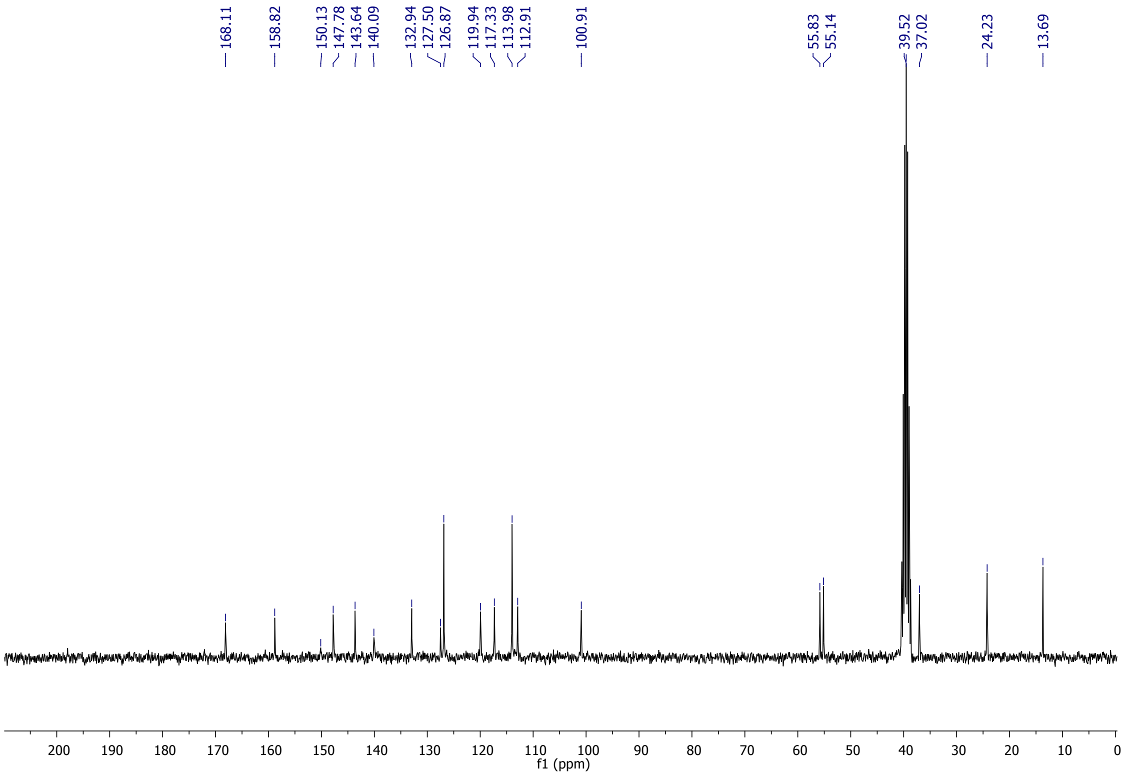

Figure S36 ^{13}C NMR spectrum of compound **16**

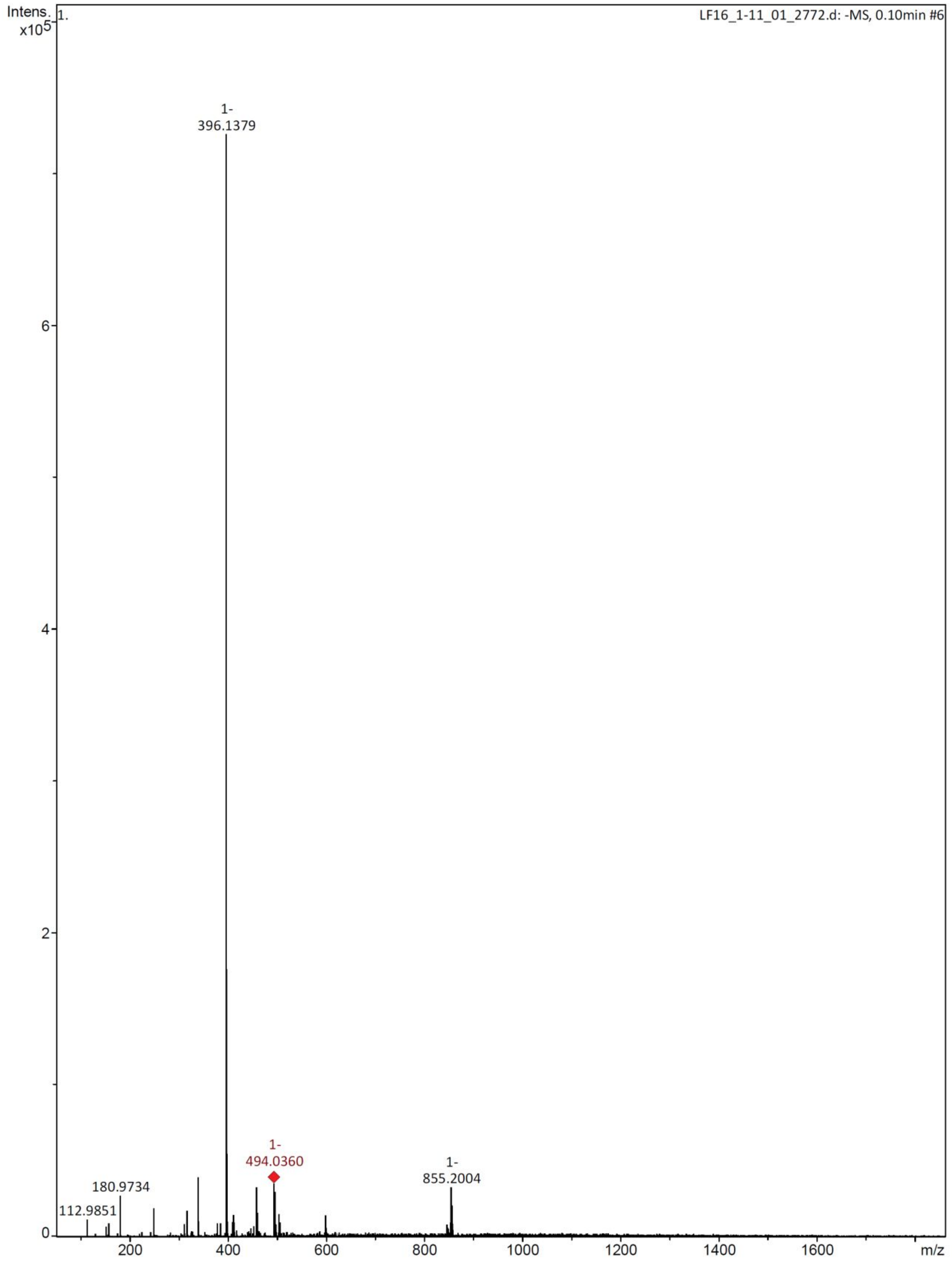

Figure S37 HRM spectrum of compound **16**

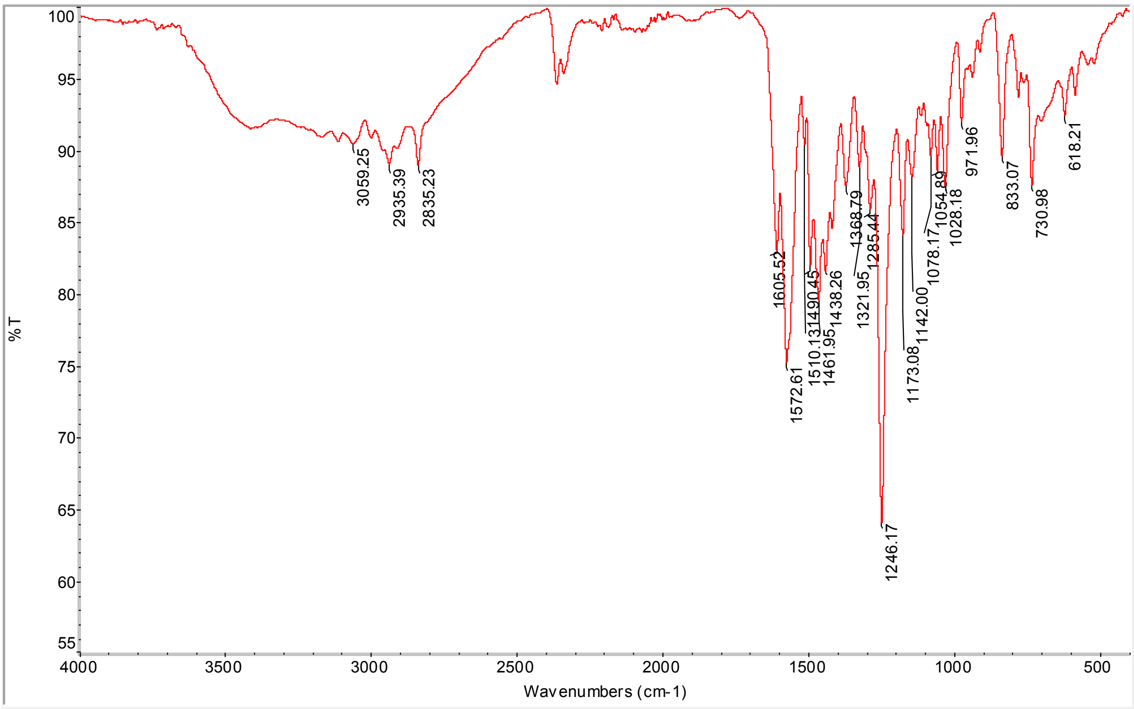

Figure S38 Infrared spectrum of compound **17**

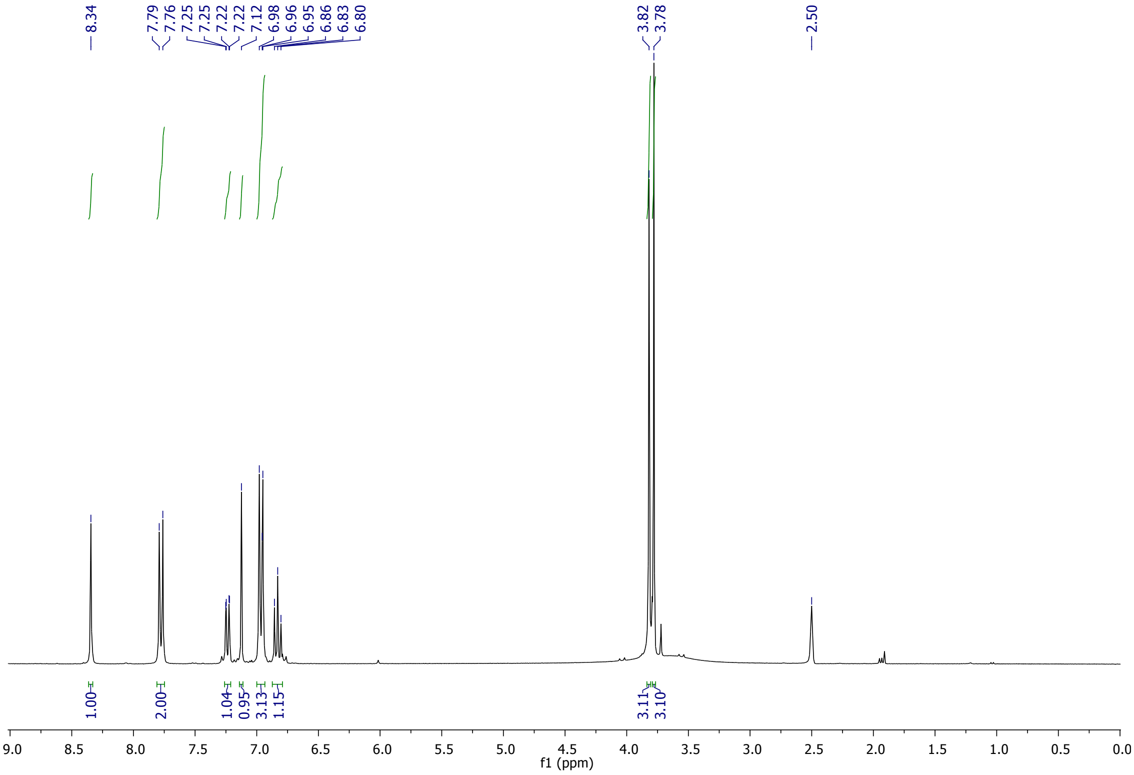

Figure S39 ^{1}H NMR spectrum of compound **17**

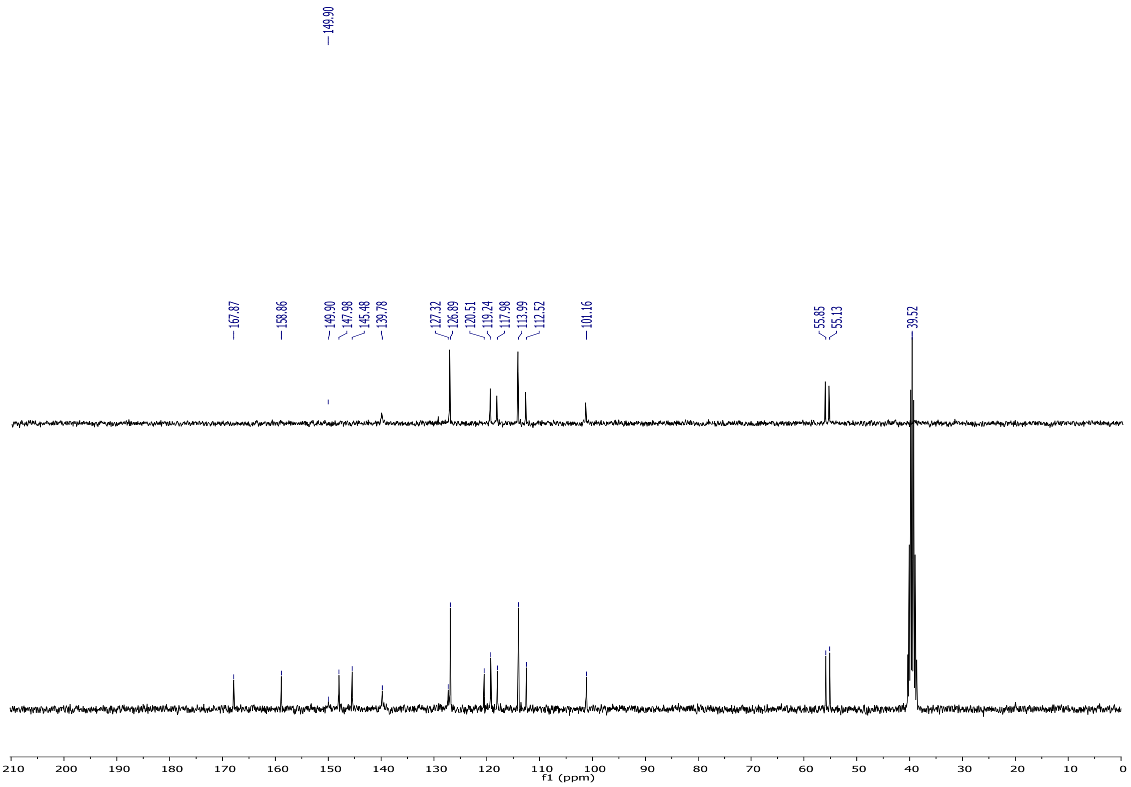

Figure S40 ^{13}C NMR spectrum of compound **17**

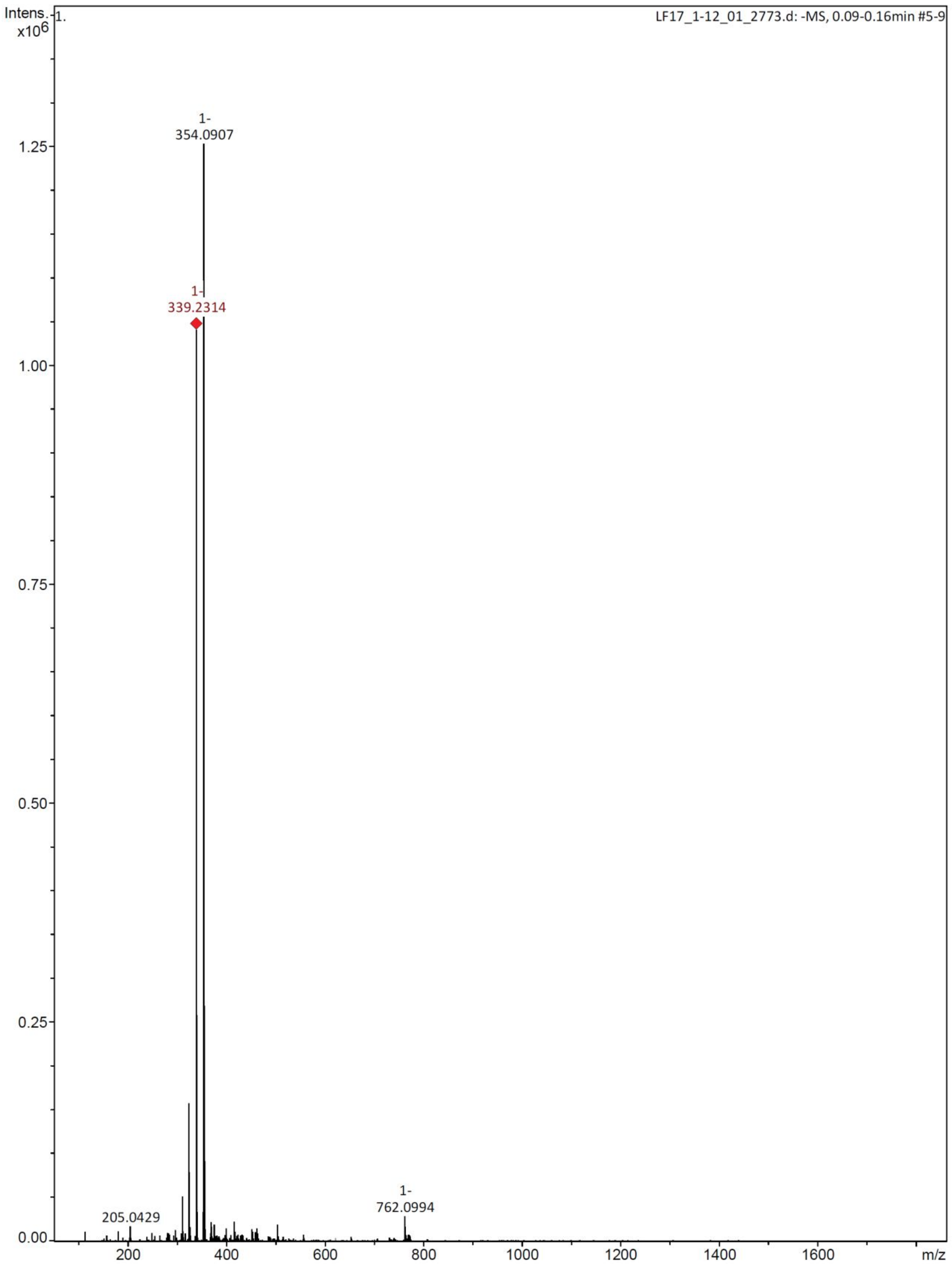

Figure S41 HRM spectrum of compound **17**

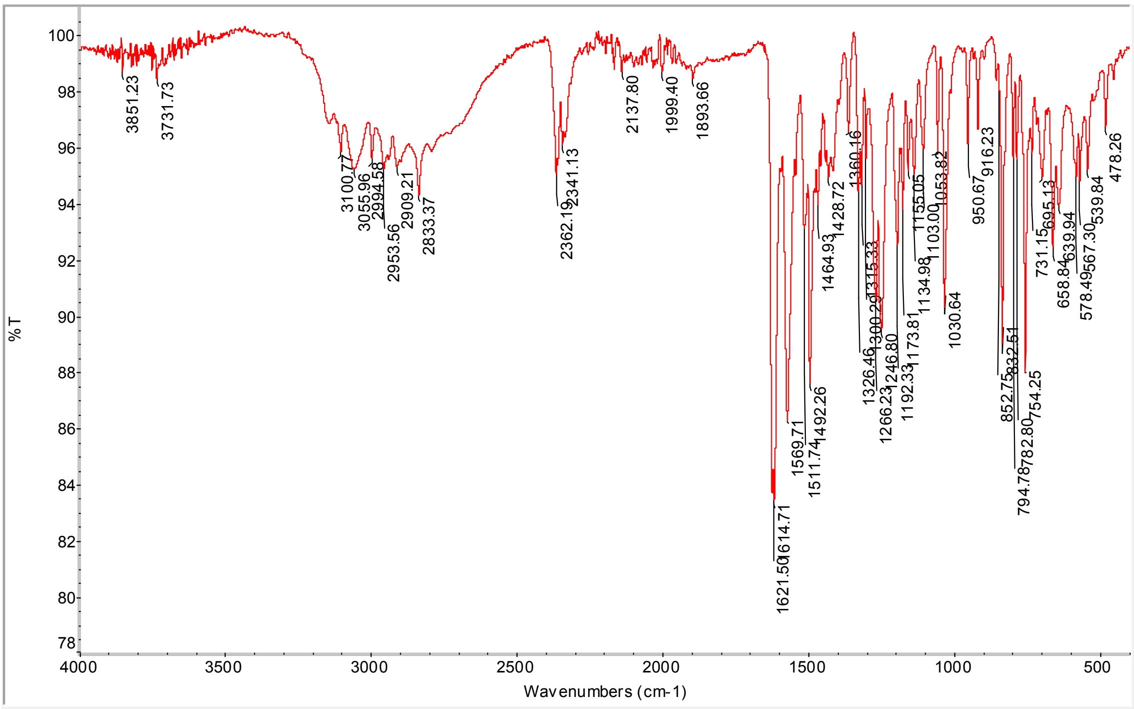

Figure S42 Infrared spectrum of compound **18**

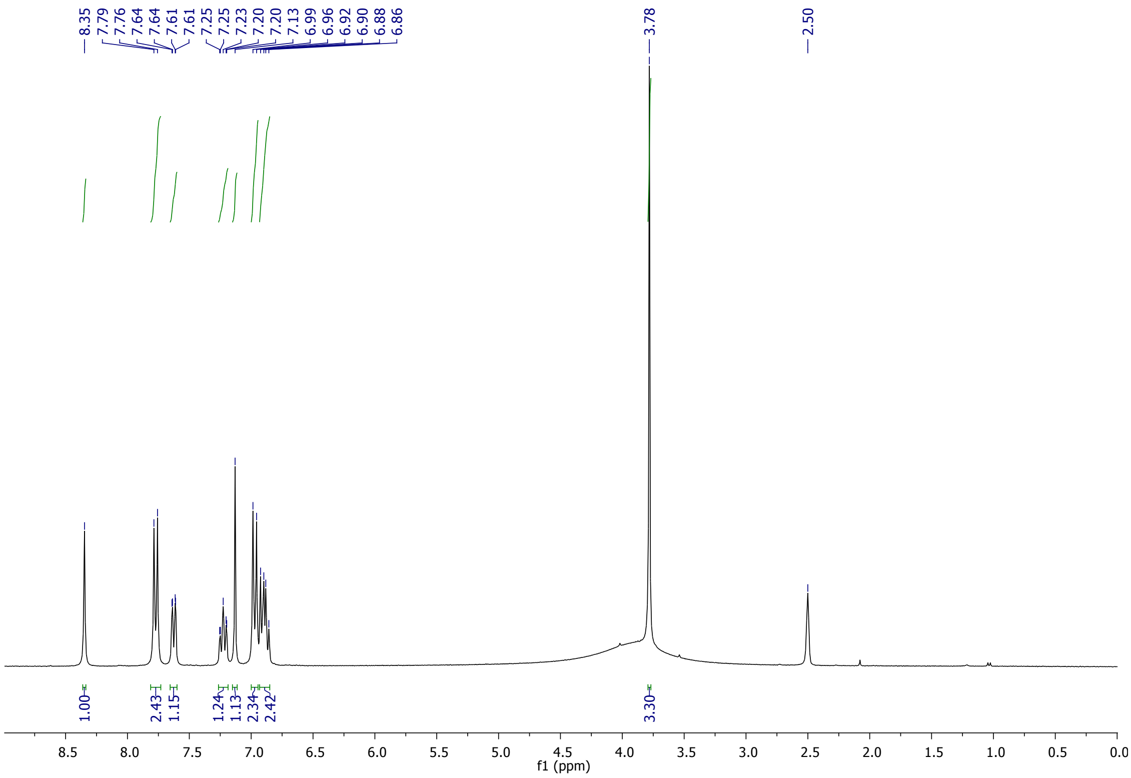

Figure S43 ^{1}H NMR spectrum of compound **18**

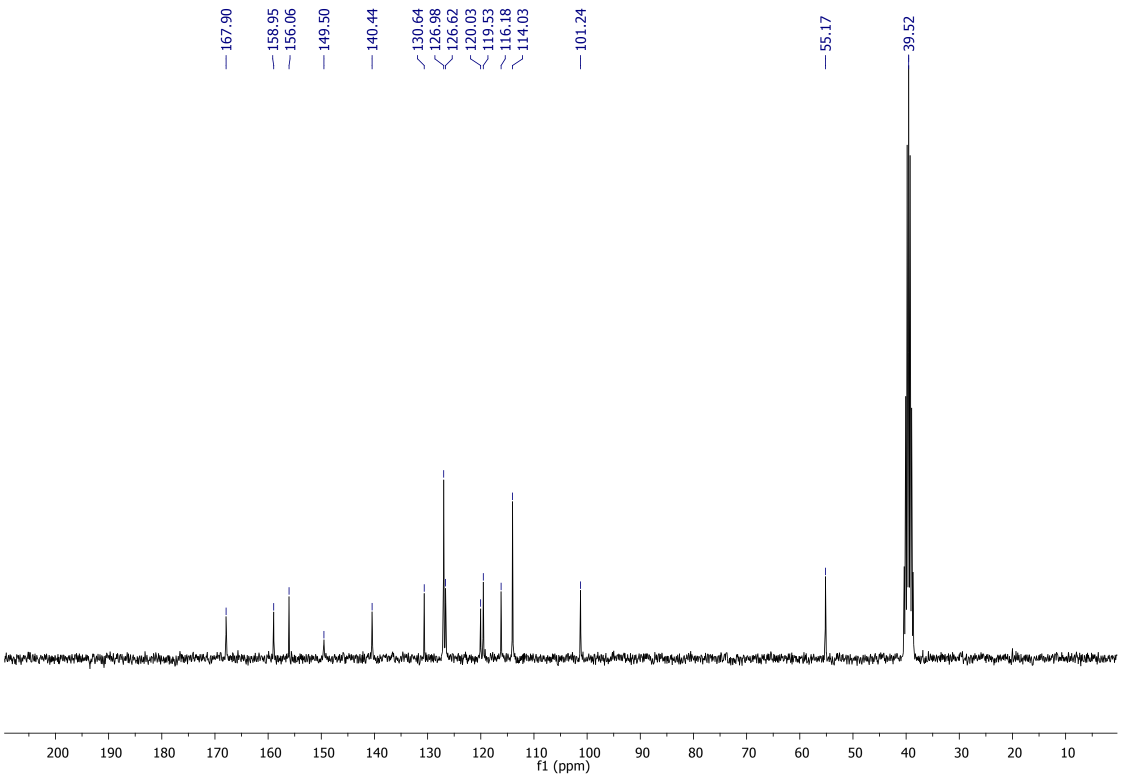

Figure S44 ^{13}C NMR spectrum of compound **18**

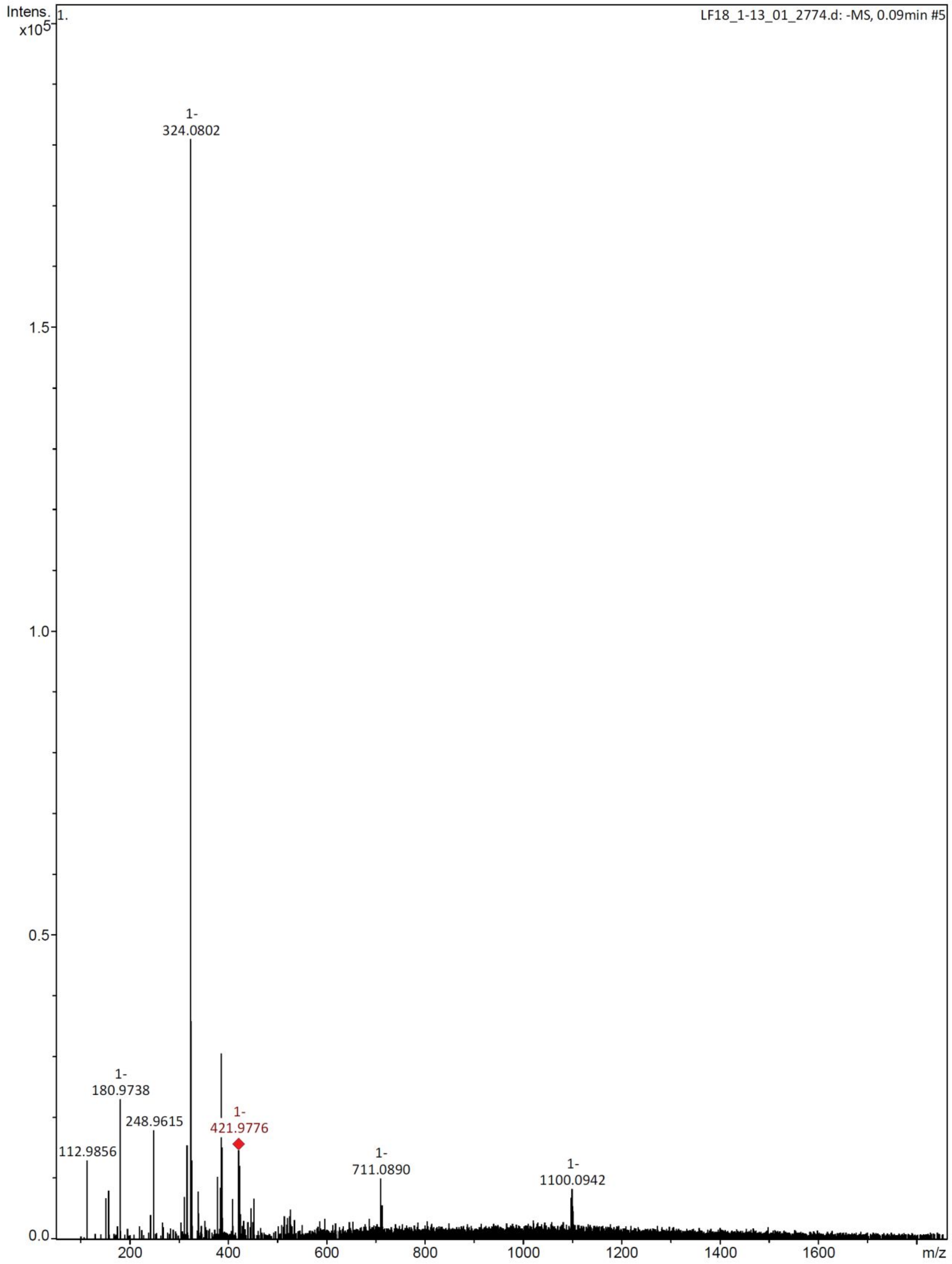

Figure S45 HRM spectrum of compound **18**

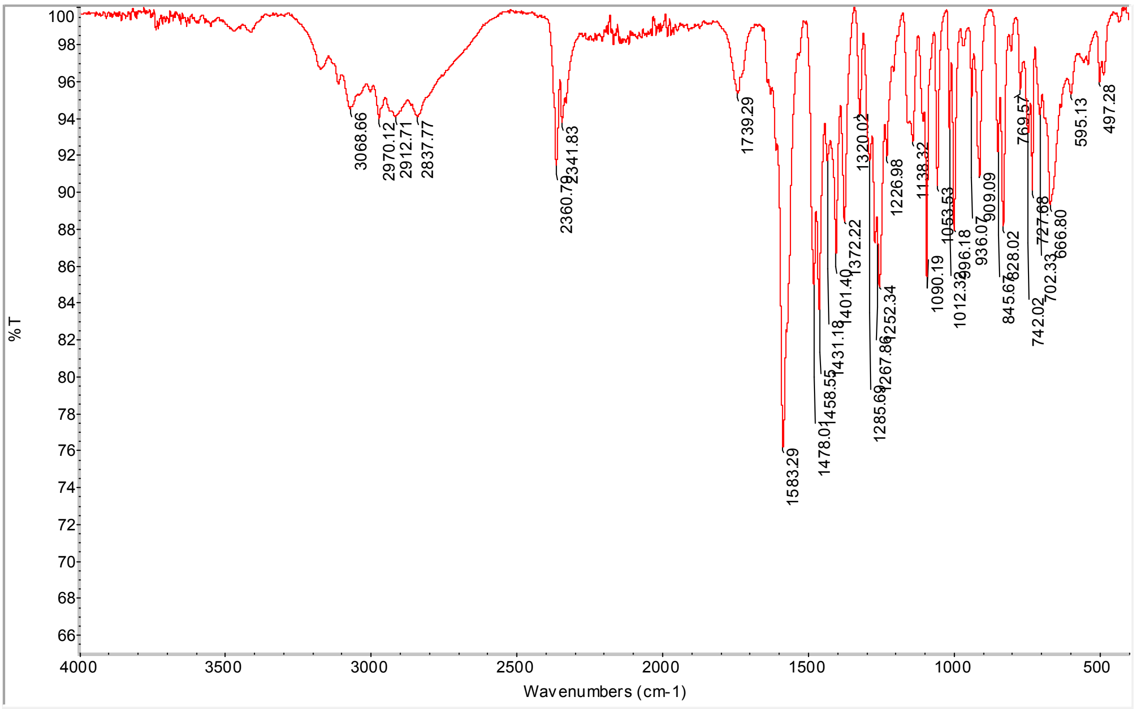

Figure S46 Infrared spectrum of compound **19**

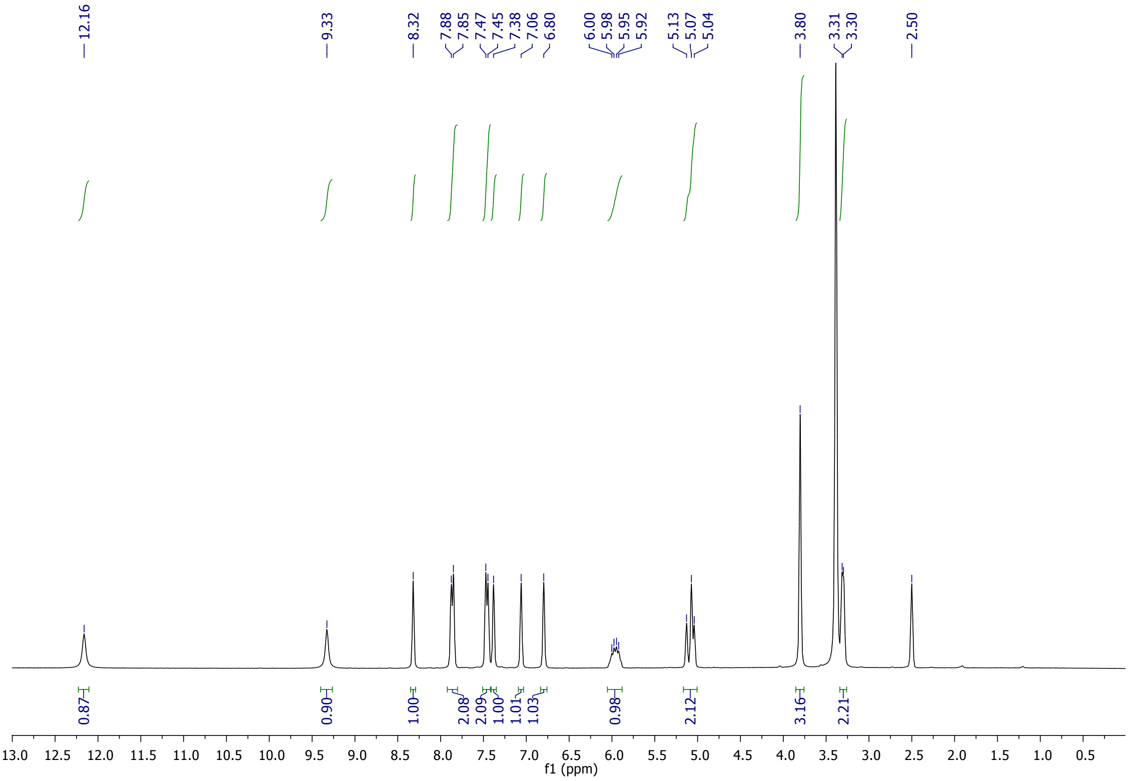

Figure S47 ^{1}H NMR spectrum of compound **19**

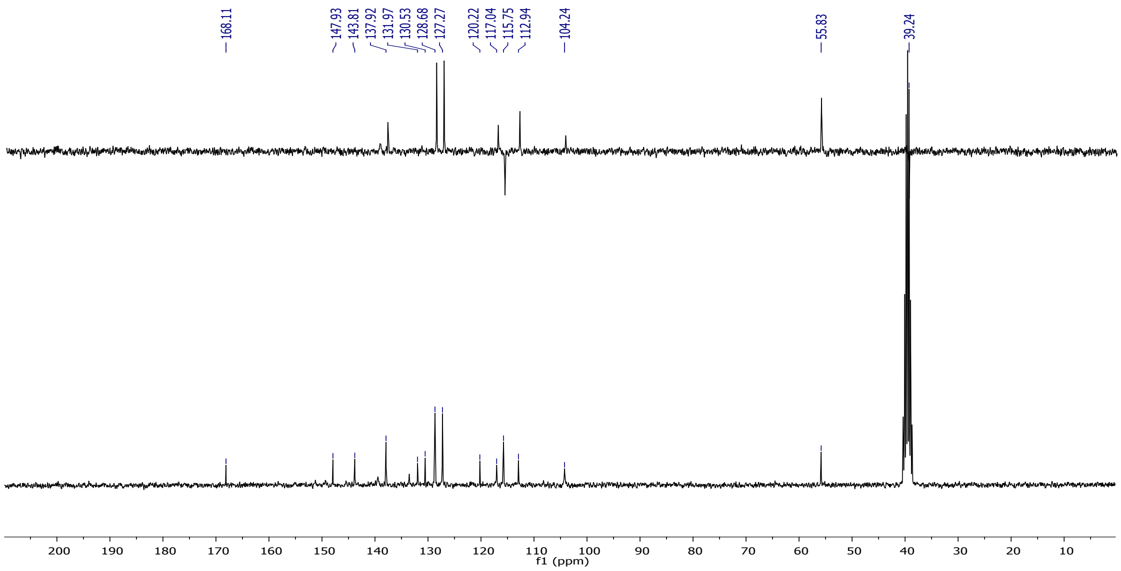

Figure S48 DEPT-135 and ^{13}C NMR spectrum of compound **19**

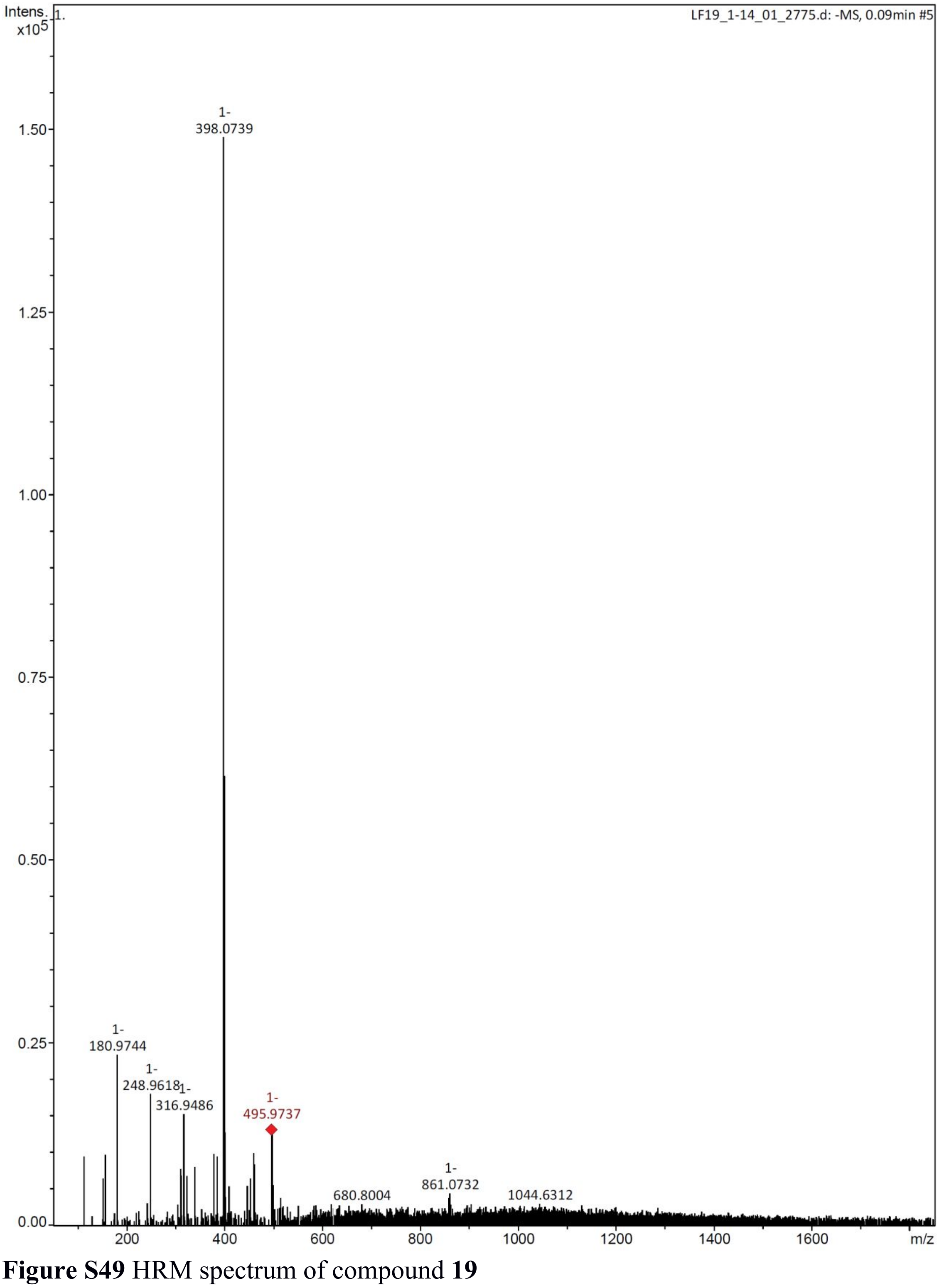

Figure S49 HRM spectrum of compound **19**

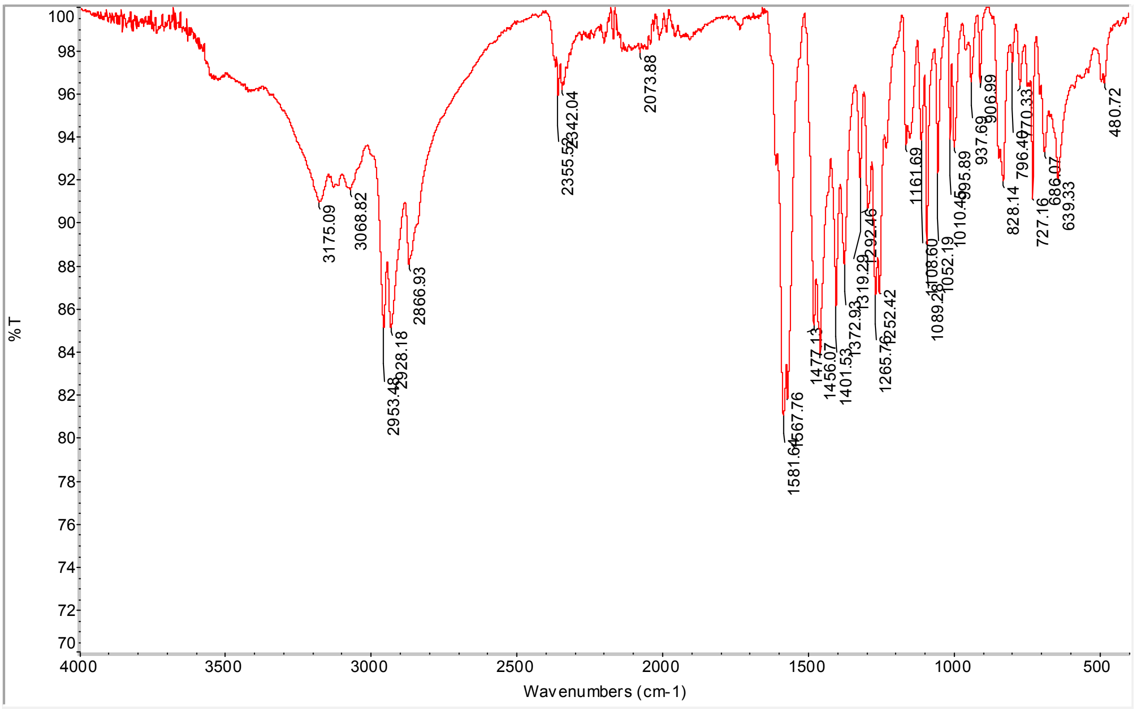

Figure S50 Infrared spectrum of compound **20**

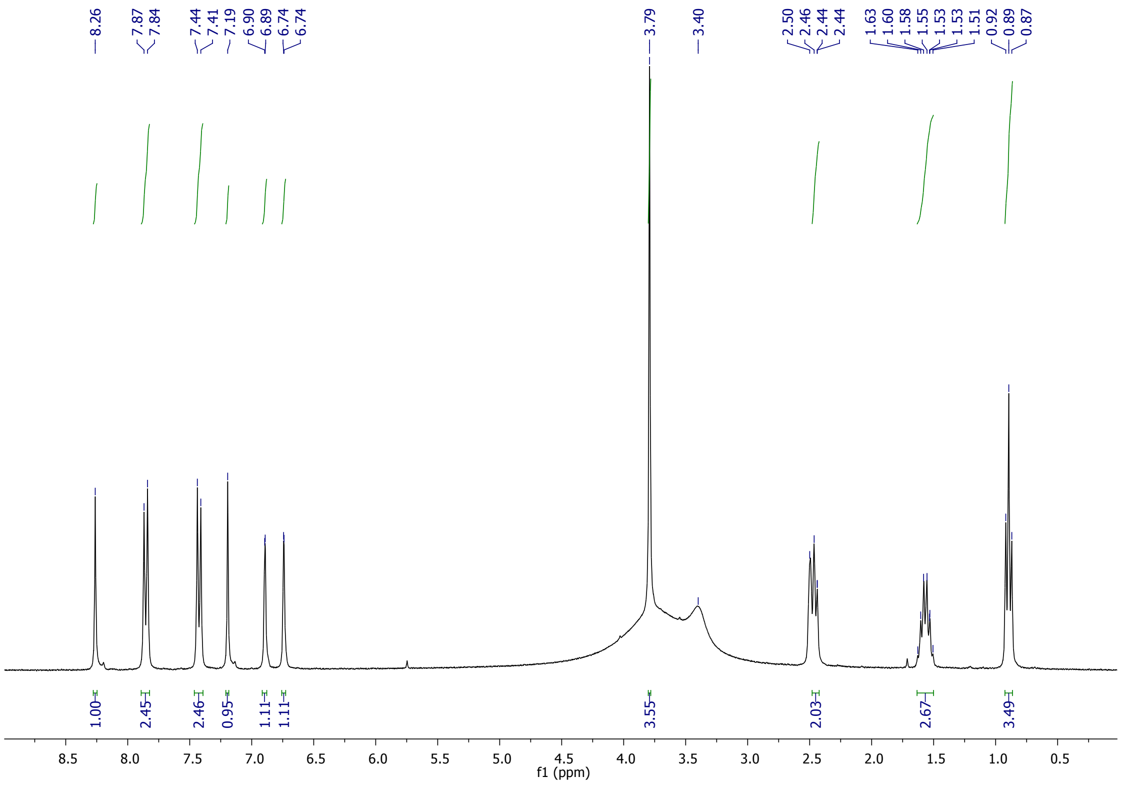

Figure S51 ^{1}H NMR spectrum of compound **20**

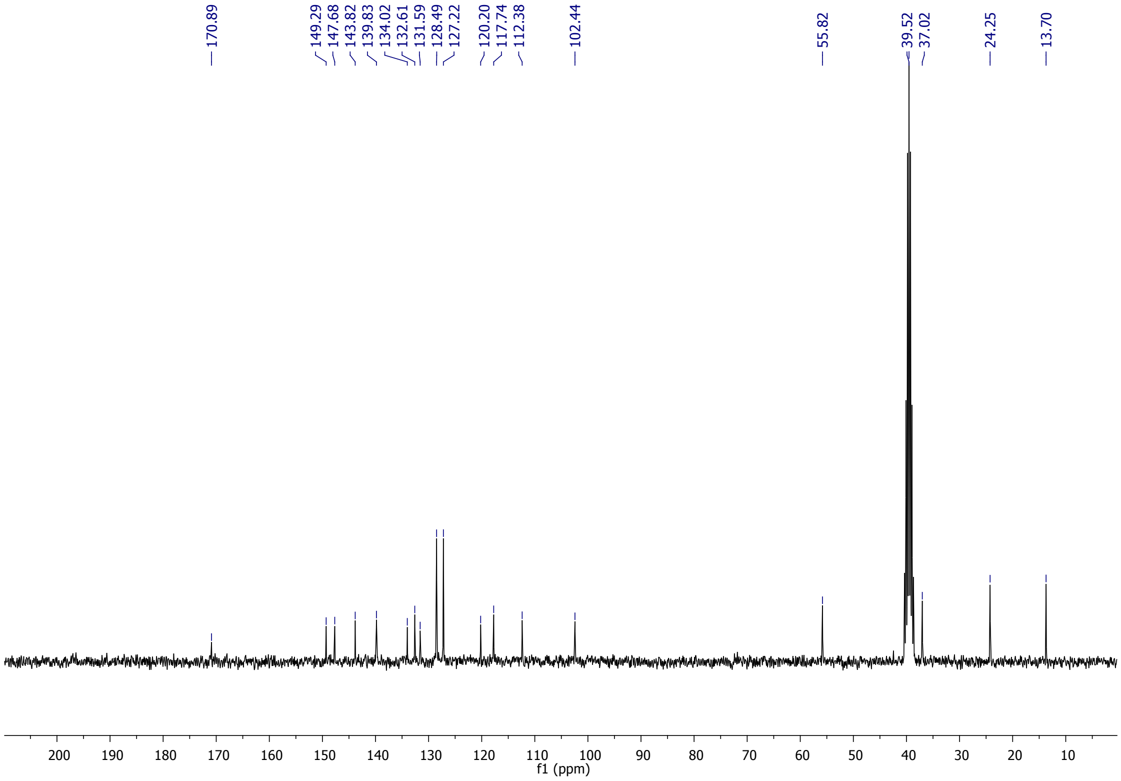

Figure S52 ^{13}C NMR spectrum of compound **20**

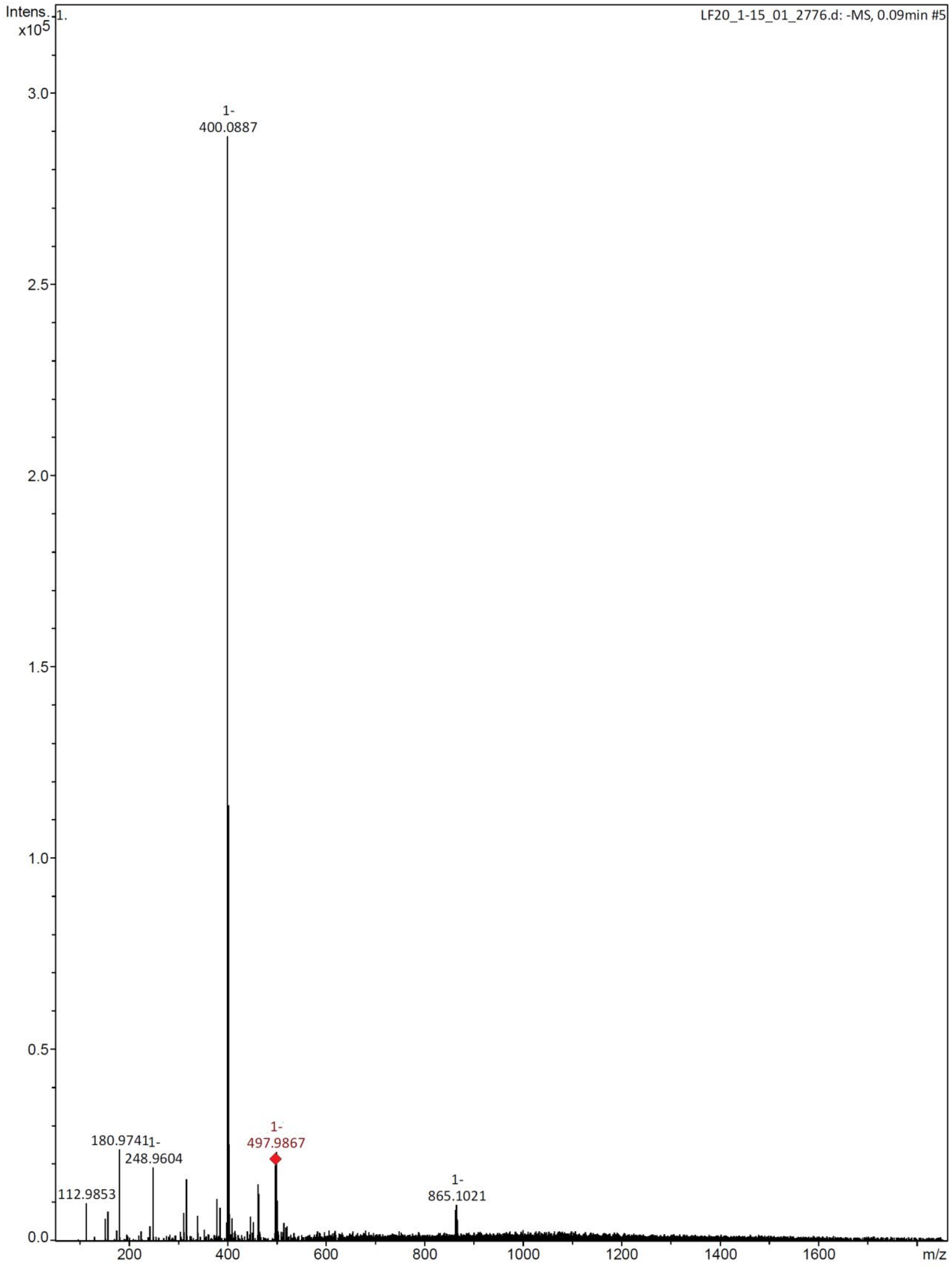

Figure S53 HRM spectrum of compound **20**

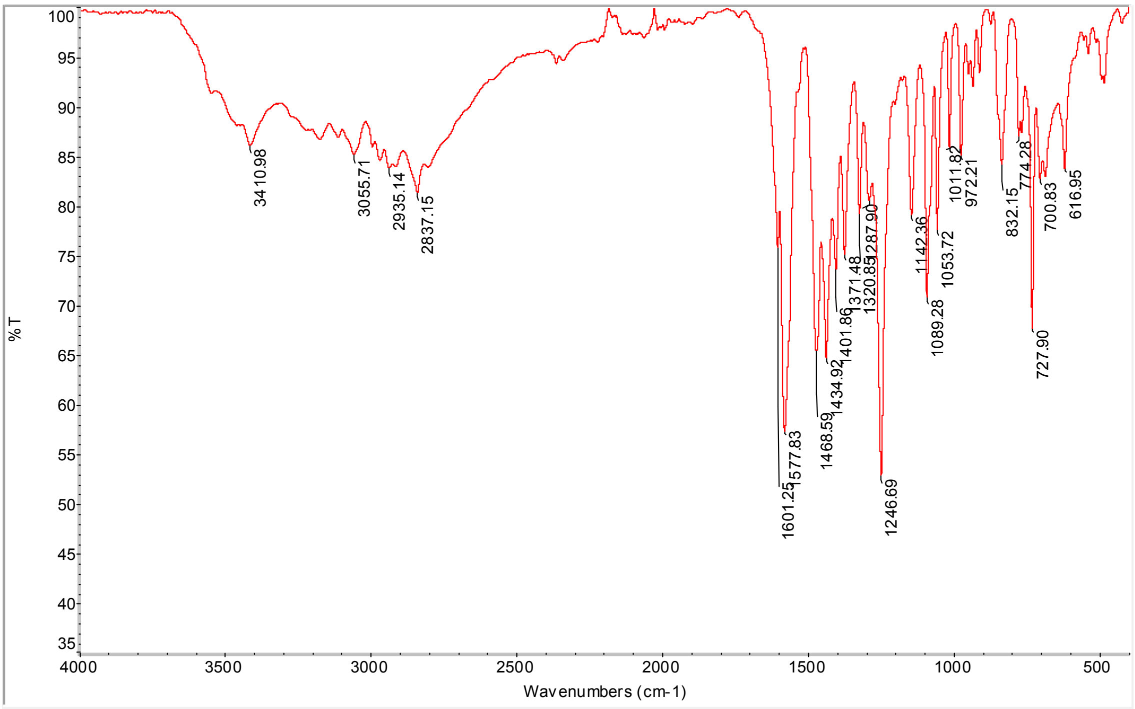

Figure S54 Infrared spectrum of compound **21**

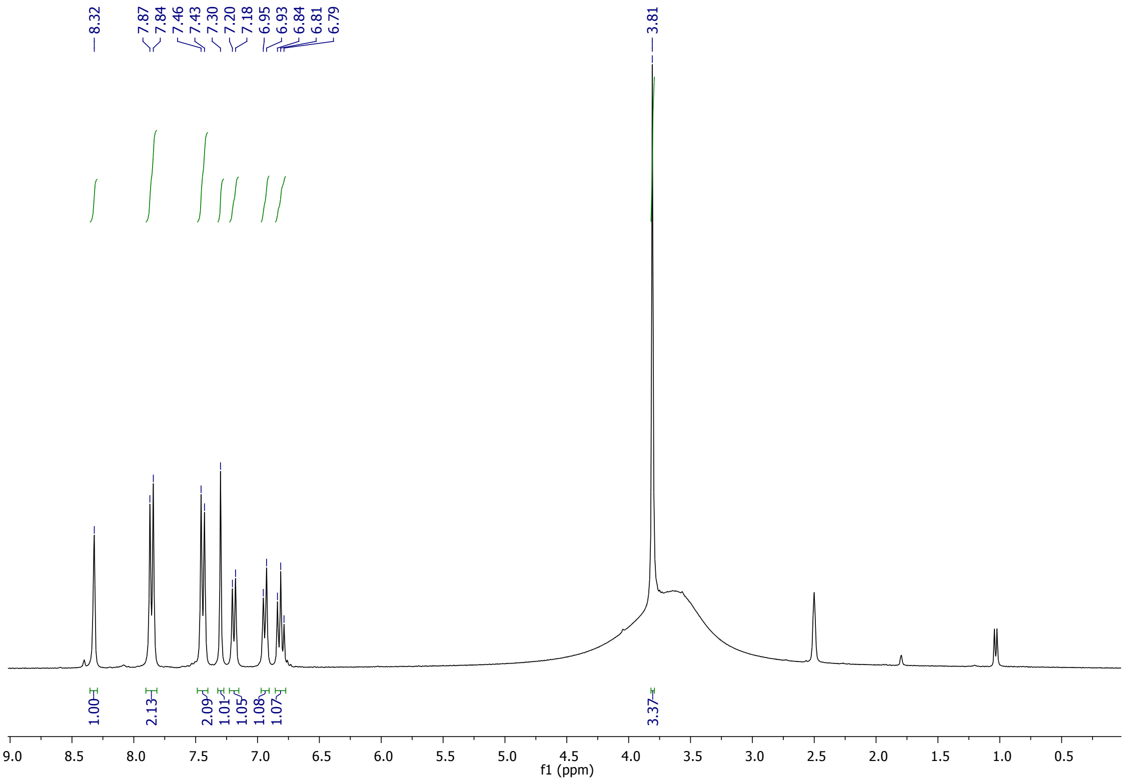

Figure S55 ^{1}H NMR spectrum of compound **21**

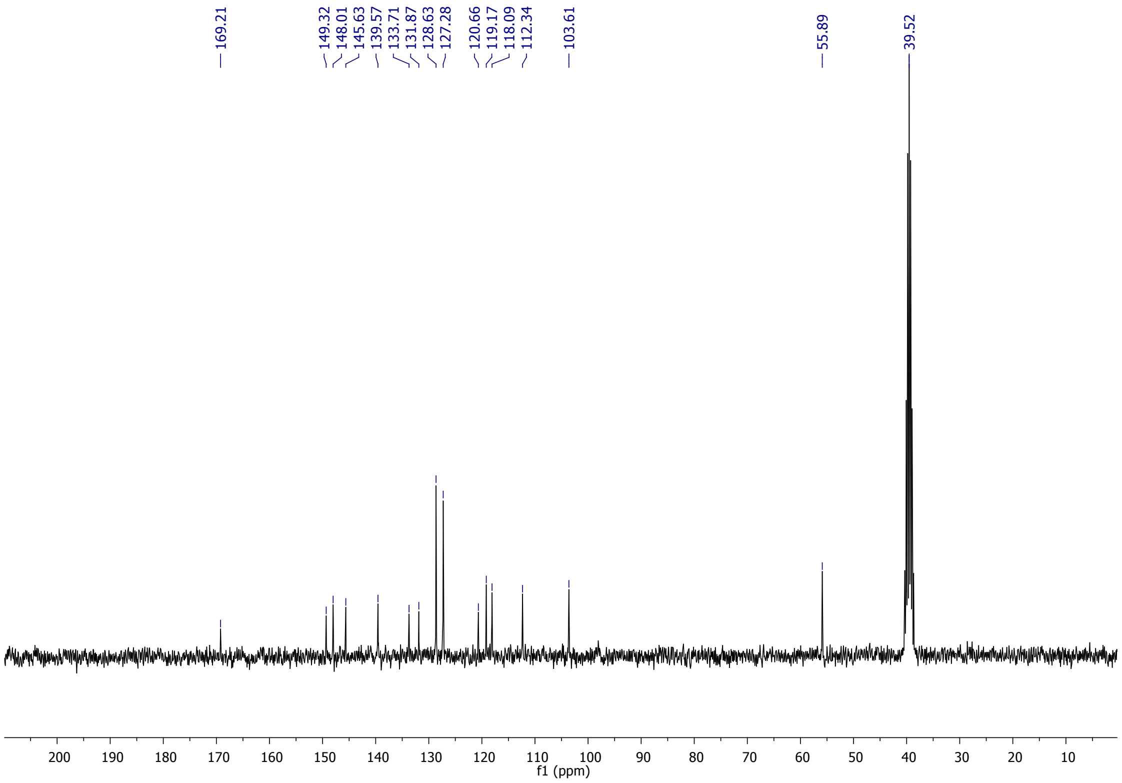

Figure S56 ^{13}C NMR spectrum of compound **21**

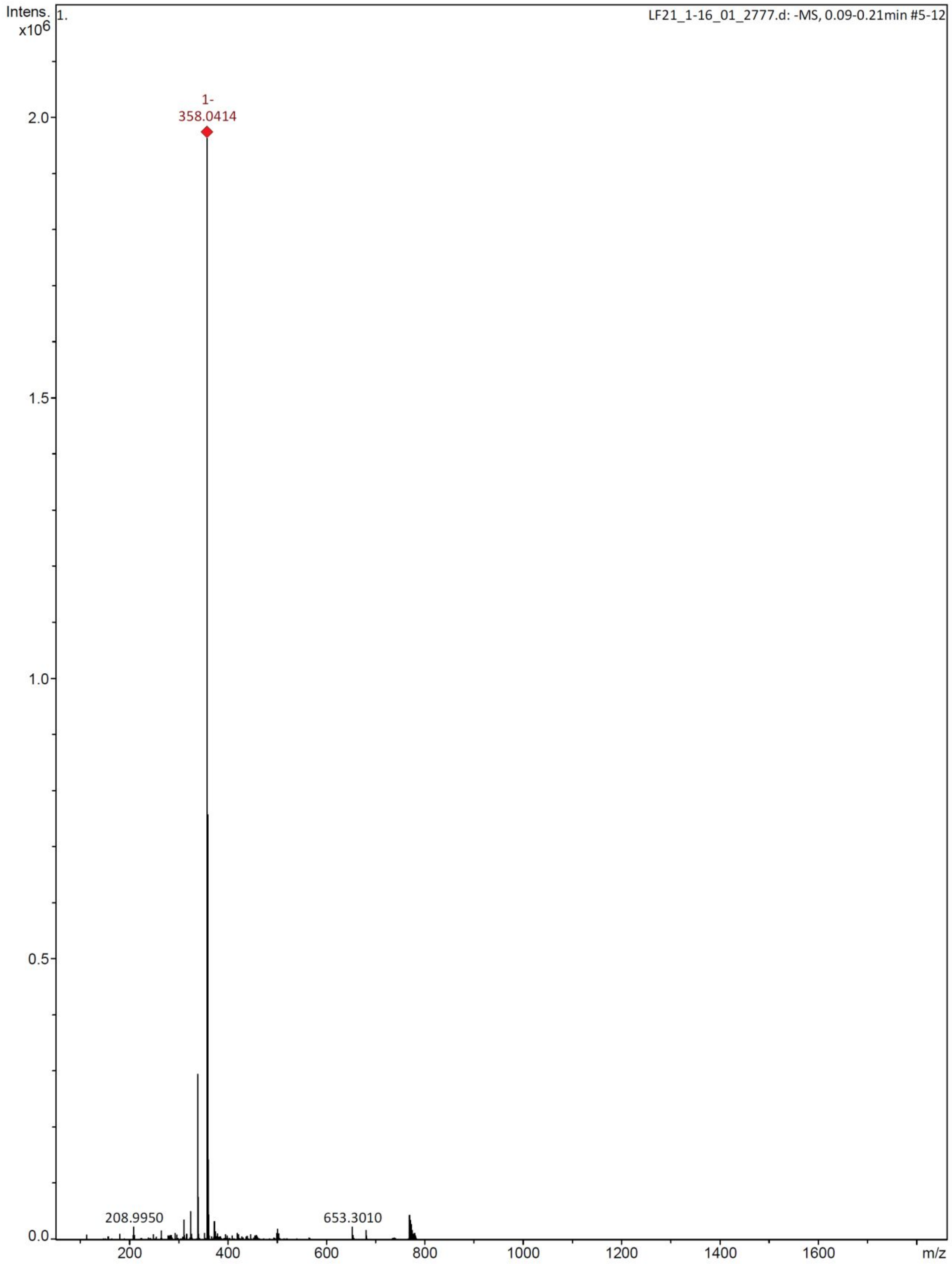

Figure S57 HRM spectrum of compound **21**

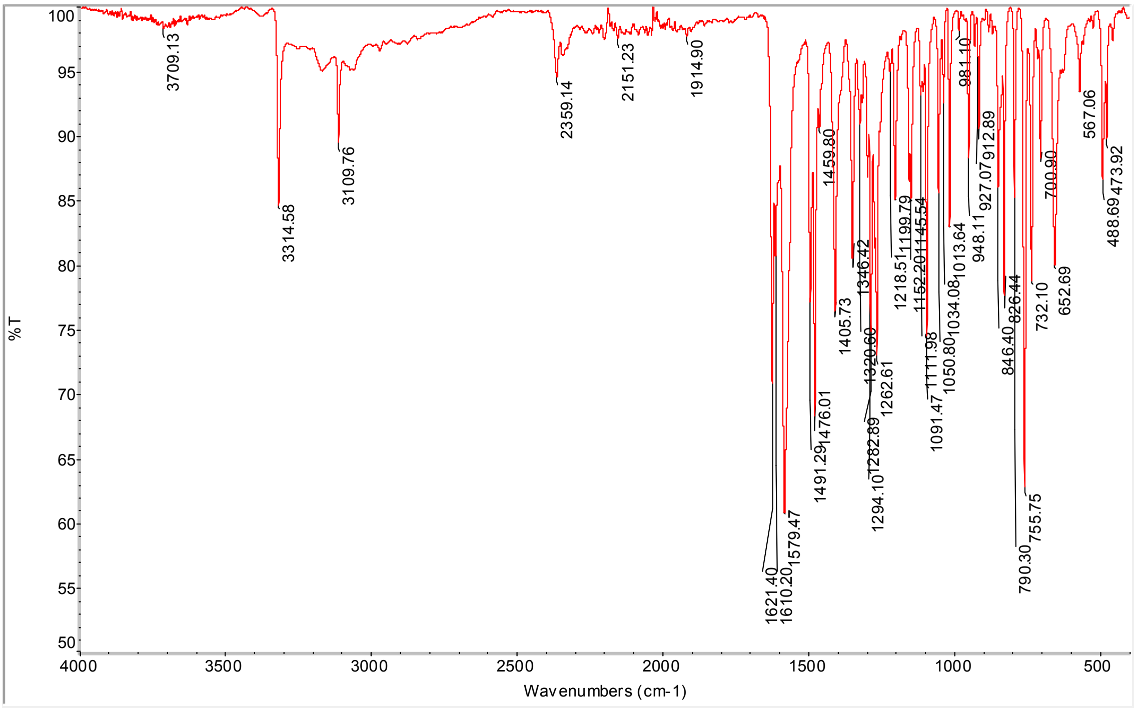

Figure S58 Infrared spectrum of compound **22**

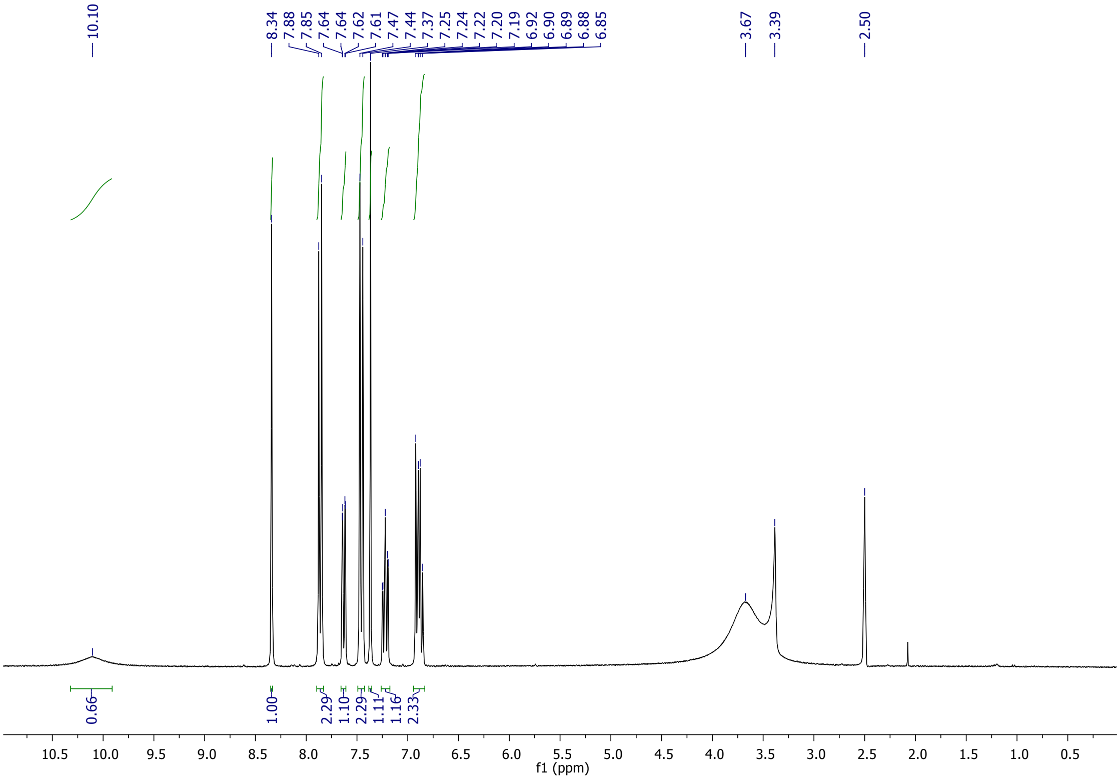

Figure S59 ^{1}H NMR spectrum of compound **22**

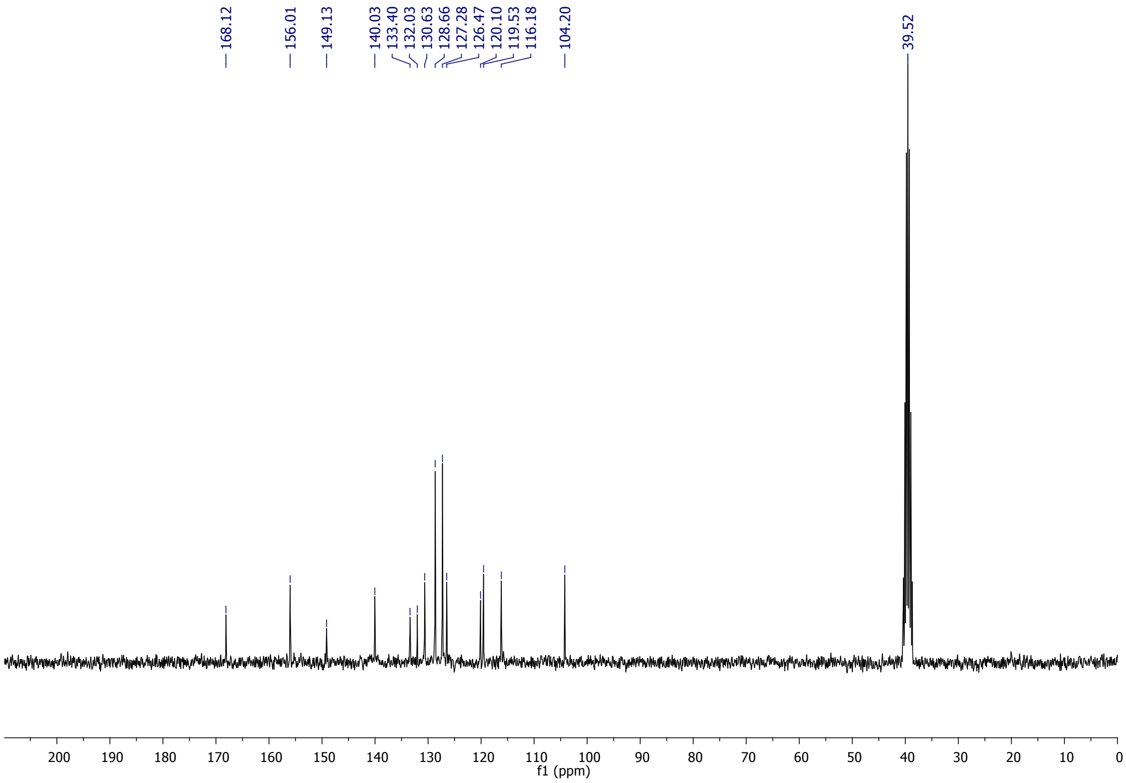

Figure S60 ^{13}C NMR spectrum of compound **22**

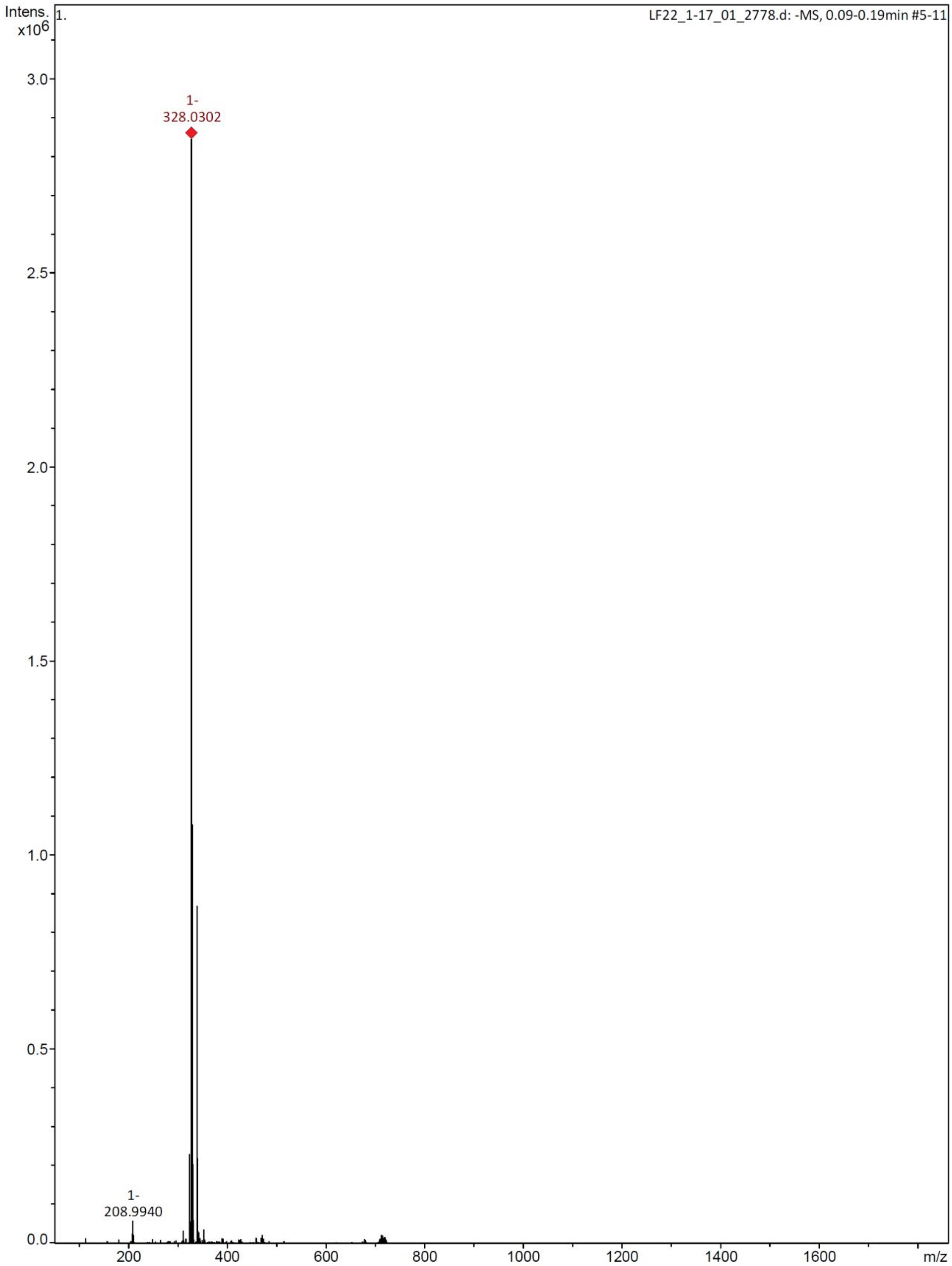

Figure S61 HRM spectrum of compound **22**

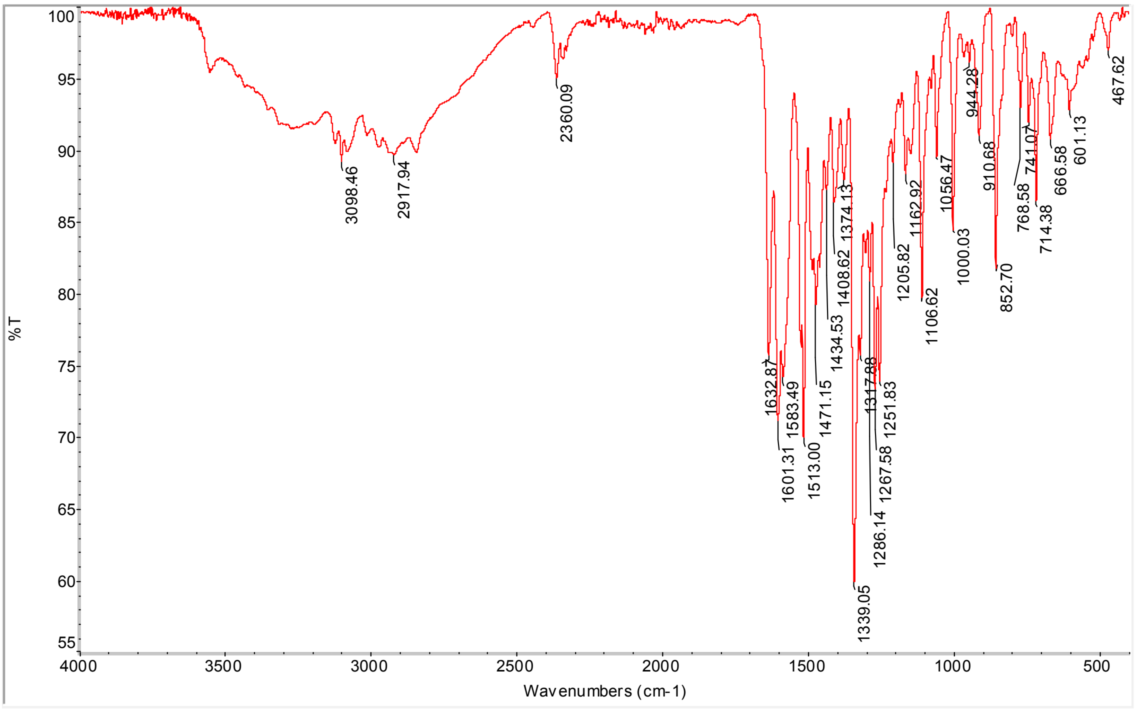

Figure S62 Infrared spectrum of compound **23**

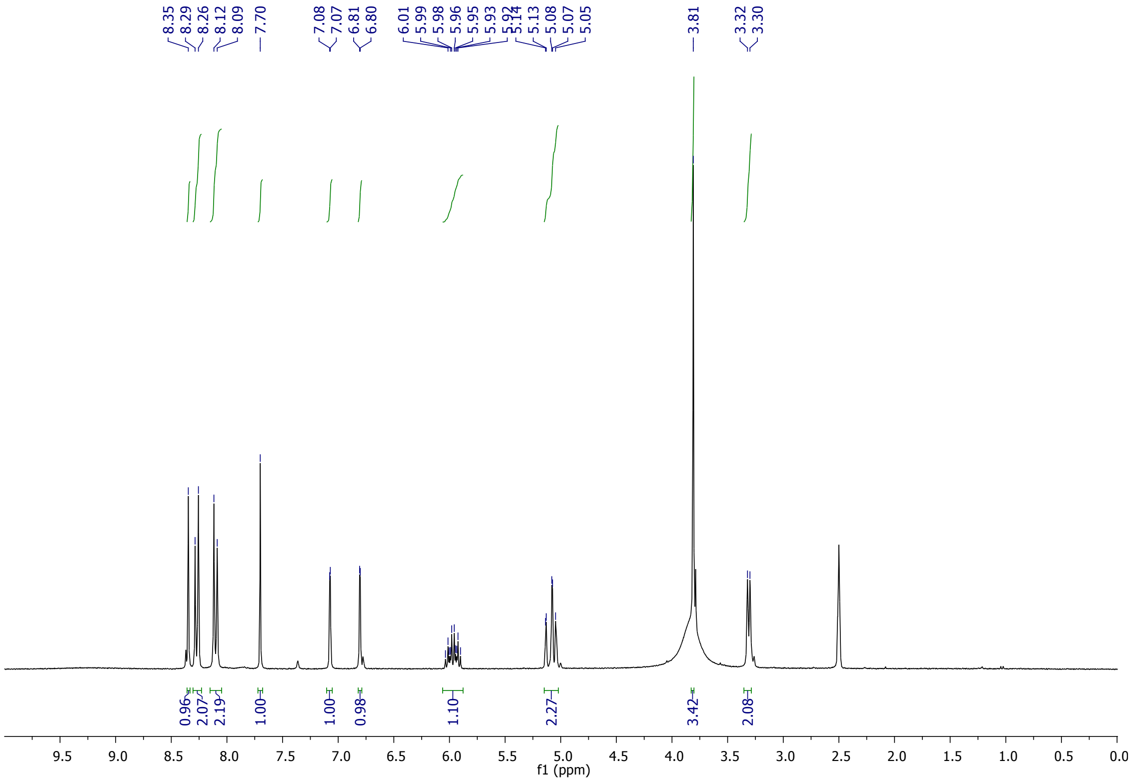

Figure S63 ^{1}H NMR spectrum of compound **23**

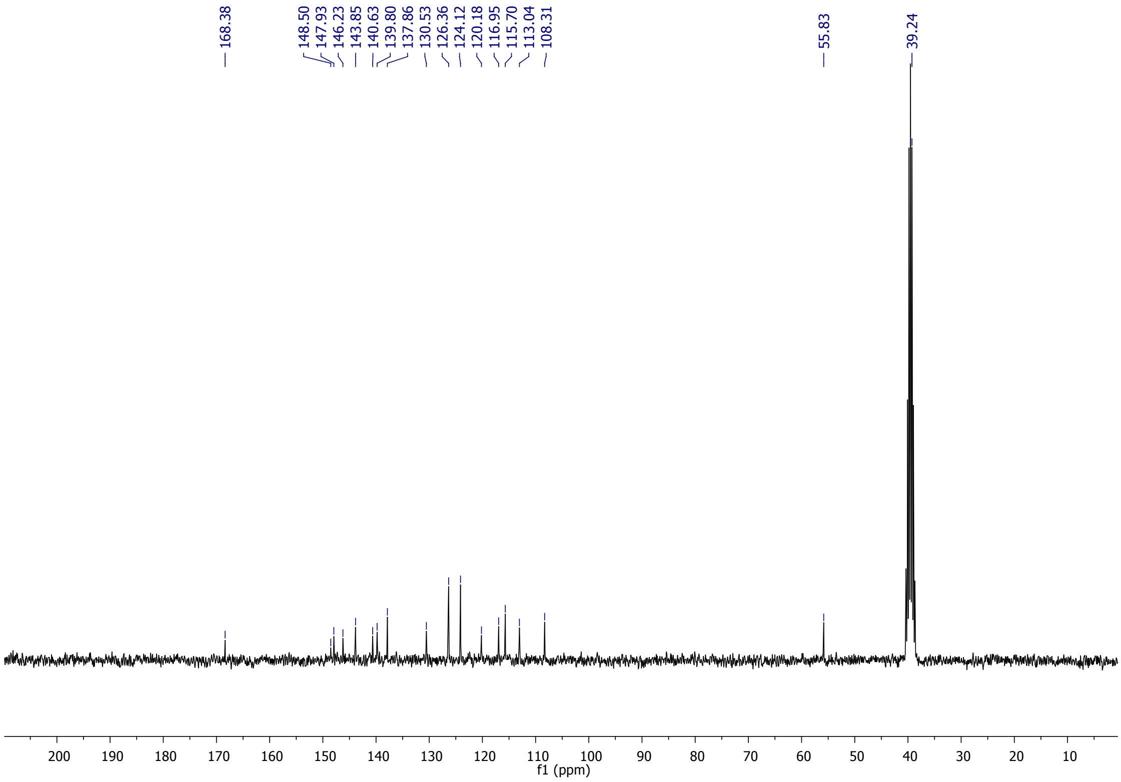

Figure S64 ^{13}C NMR spectrum of compound **23**

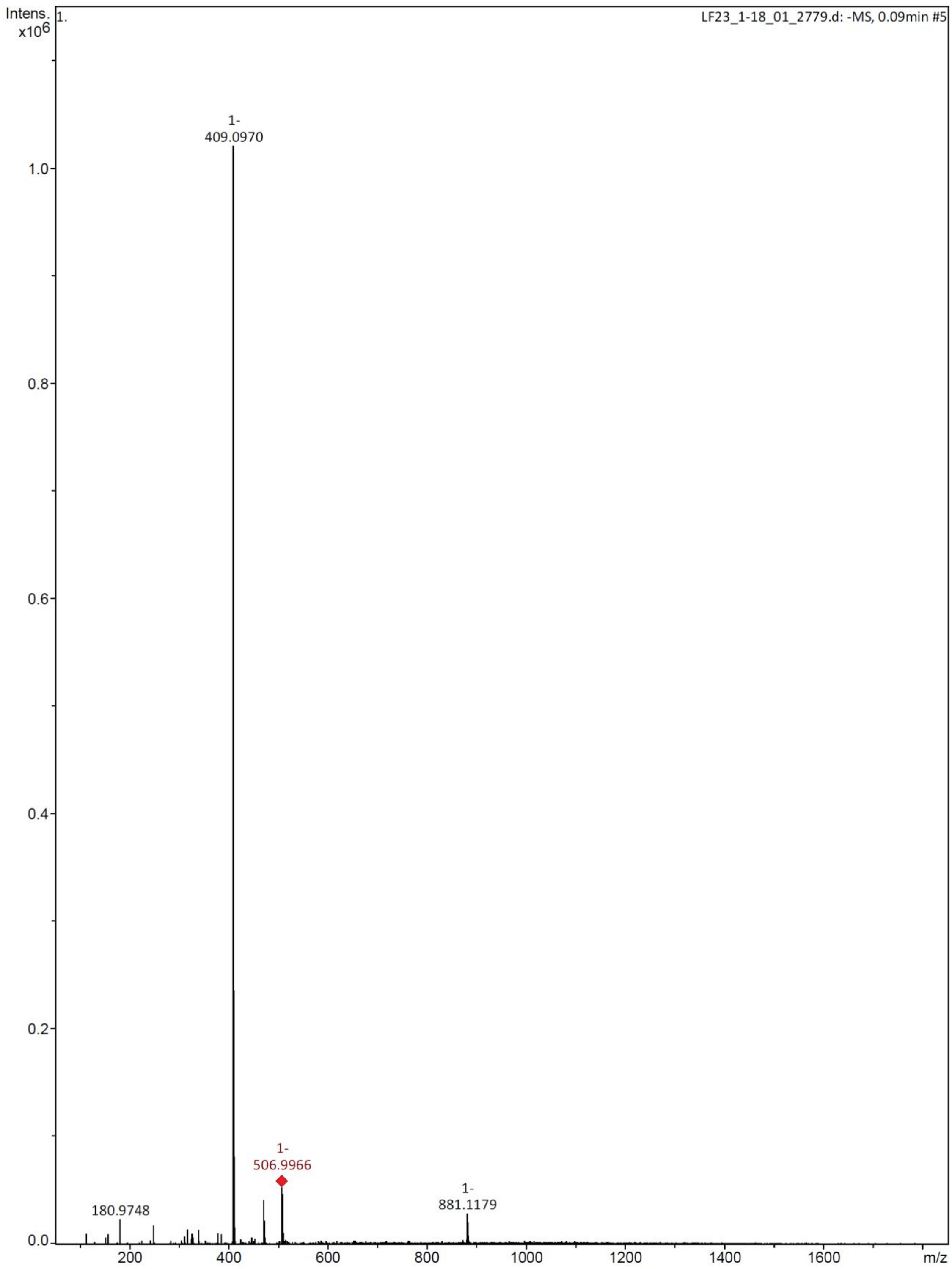

Figure S65 HRM spectrum of compound **23**

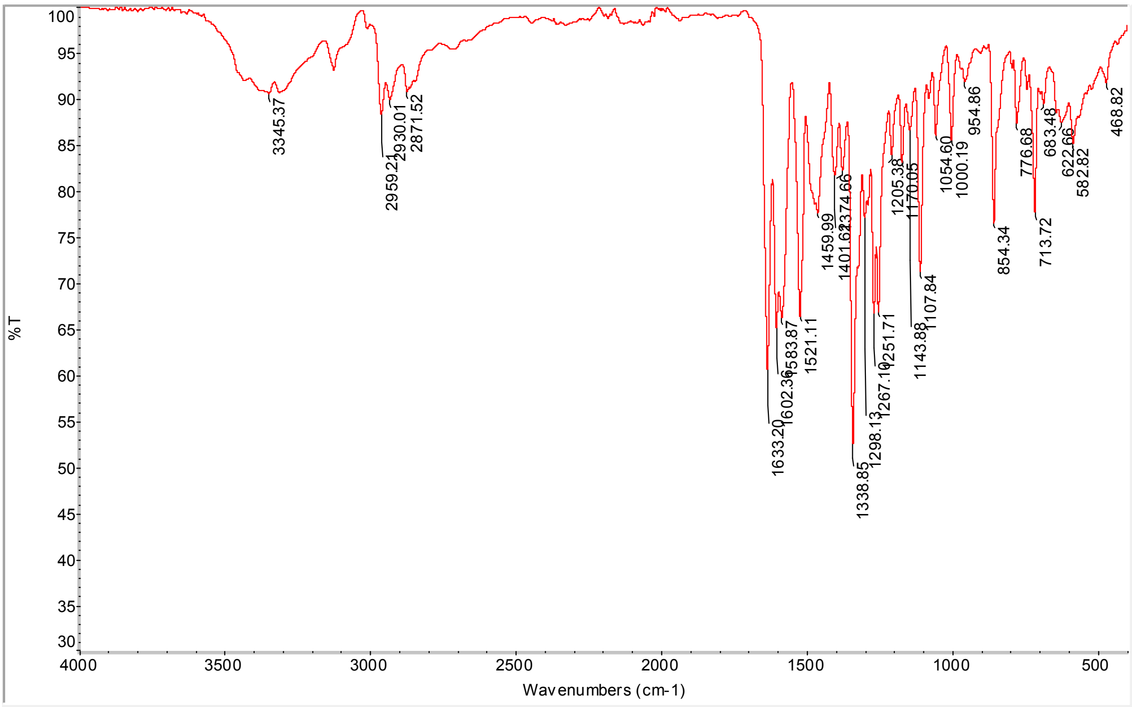

Figure S66 Infrared spectrum of compound **24**

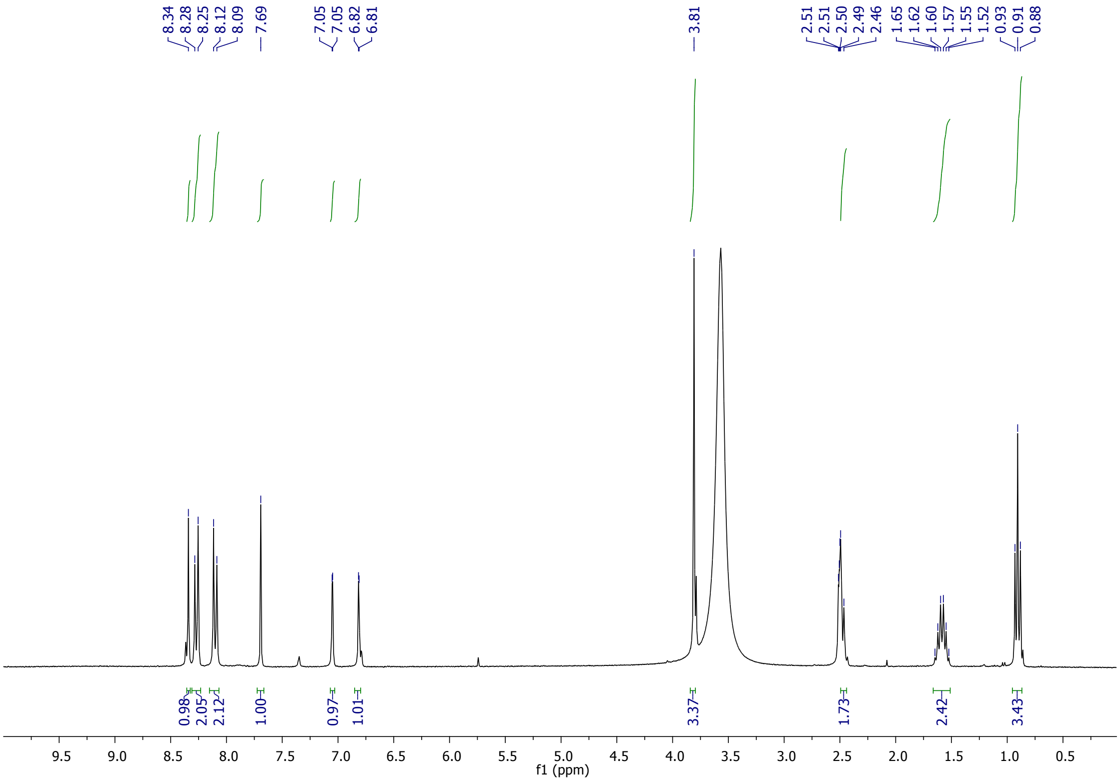

Figure S67 ^{1}H NMR spectrum of compound **24**

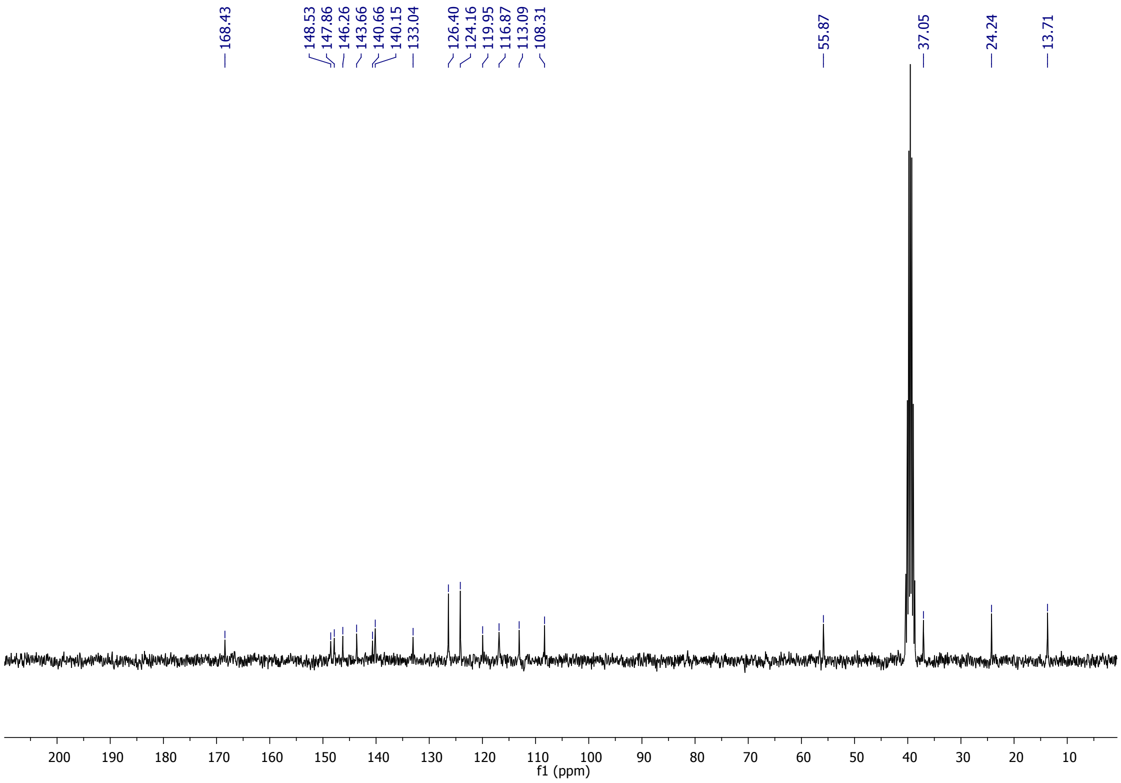

Figure S68 ^{13}C NMR spectrum of compound **24**

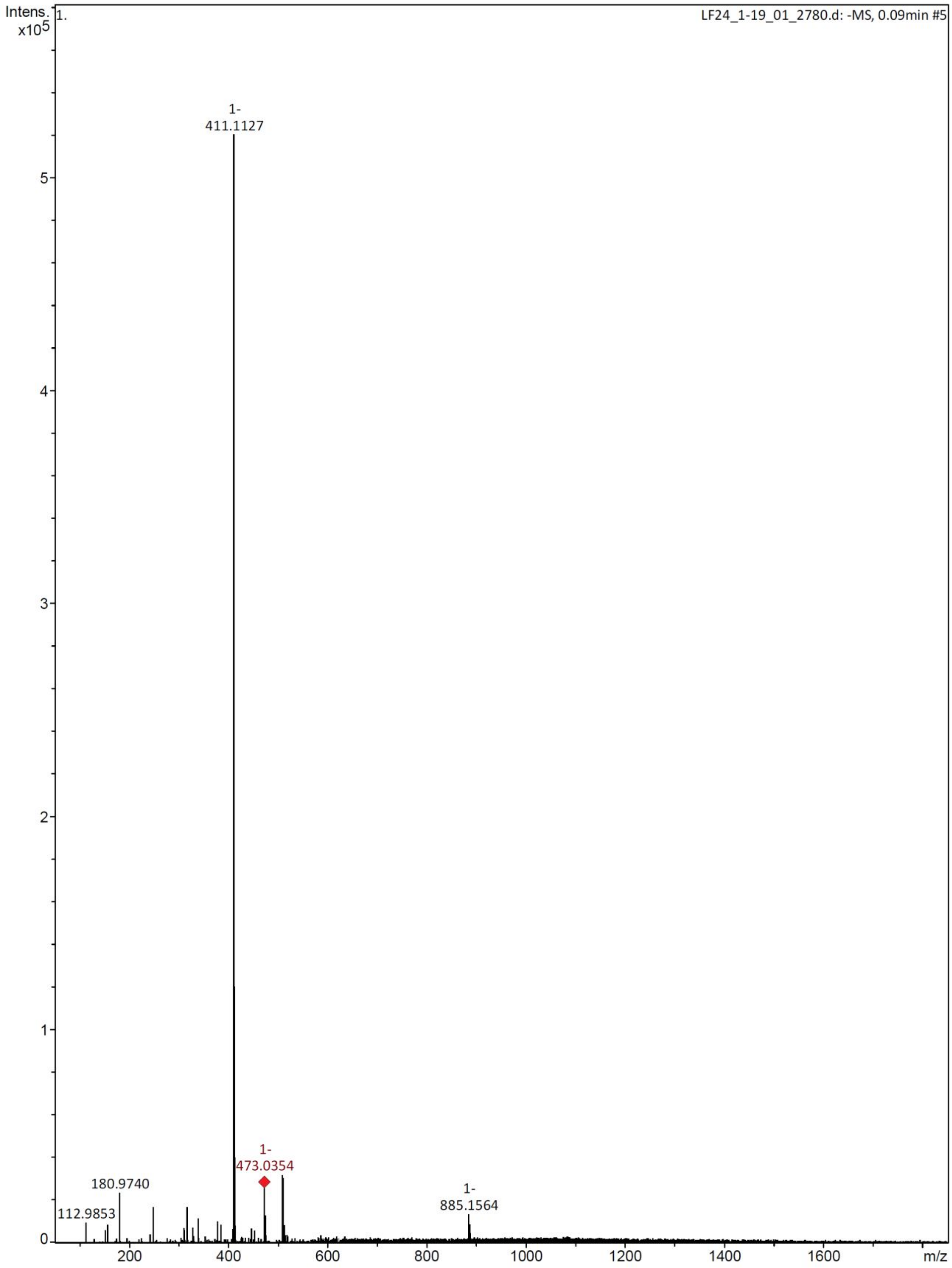

Figure S69 HRM spectrum of compound **24**

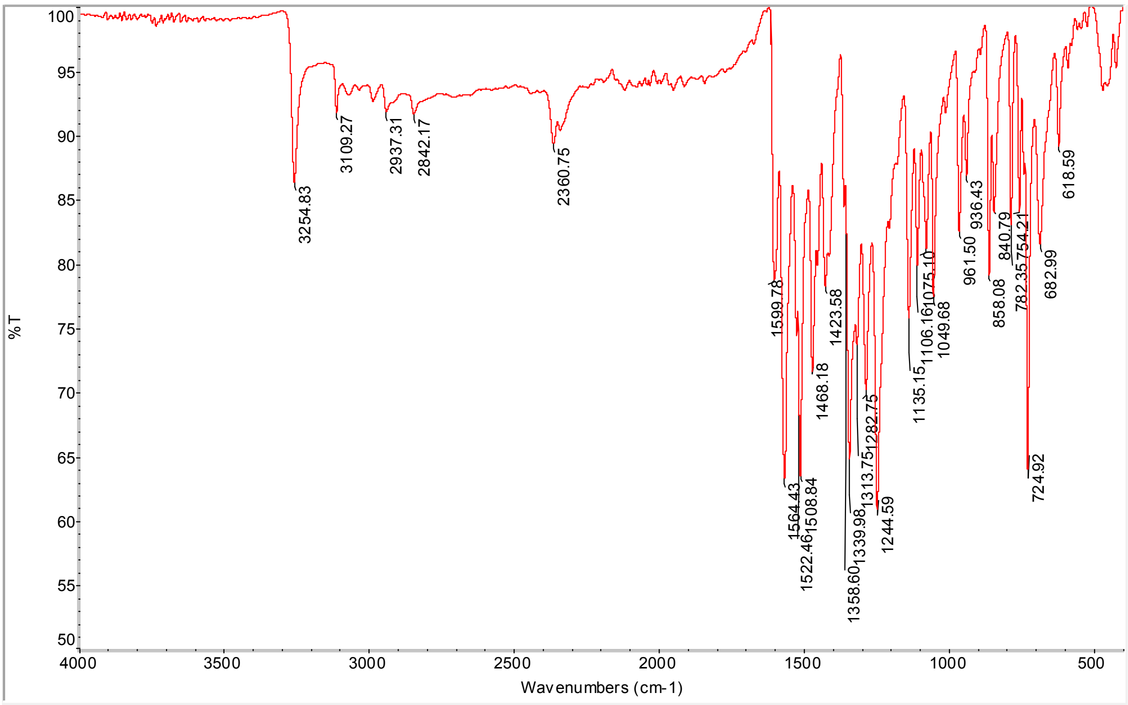

Figure S70 Infrared spectrum of compound **25**

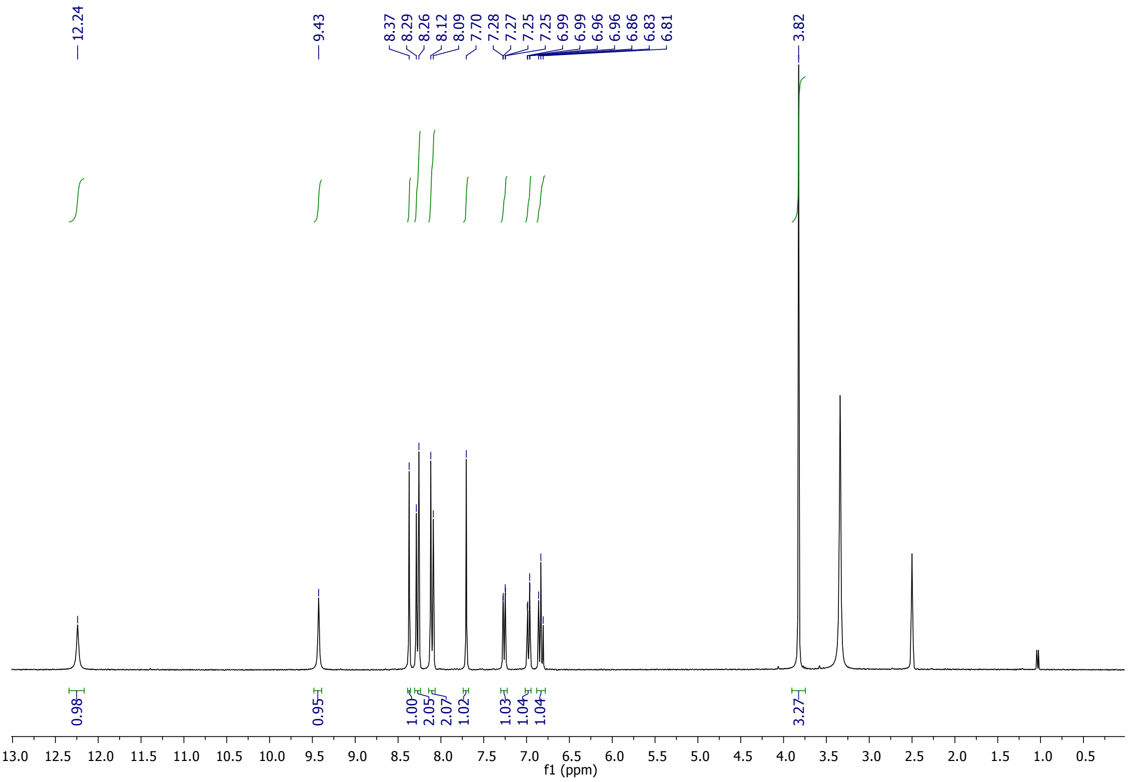

Figure S71 ^{1}H NMR spectrum of compound **25**

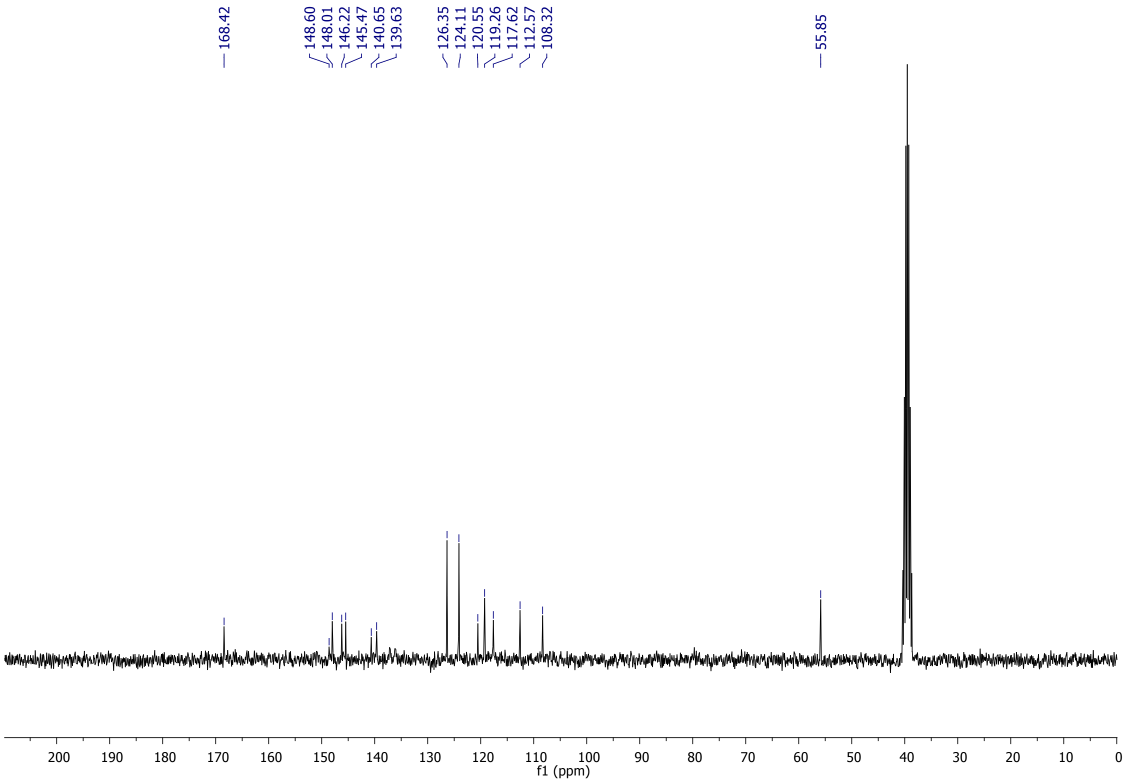

Figure S72 ^{13}C NMR spectrum of compound **25**

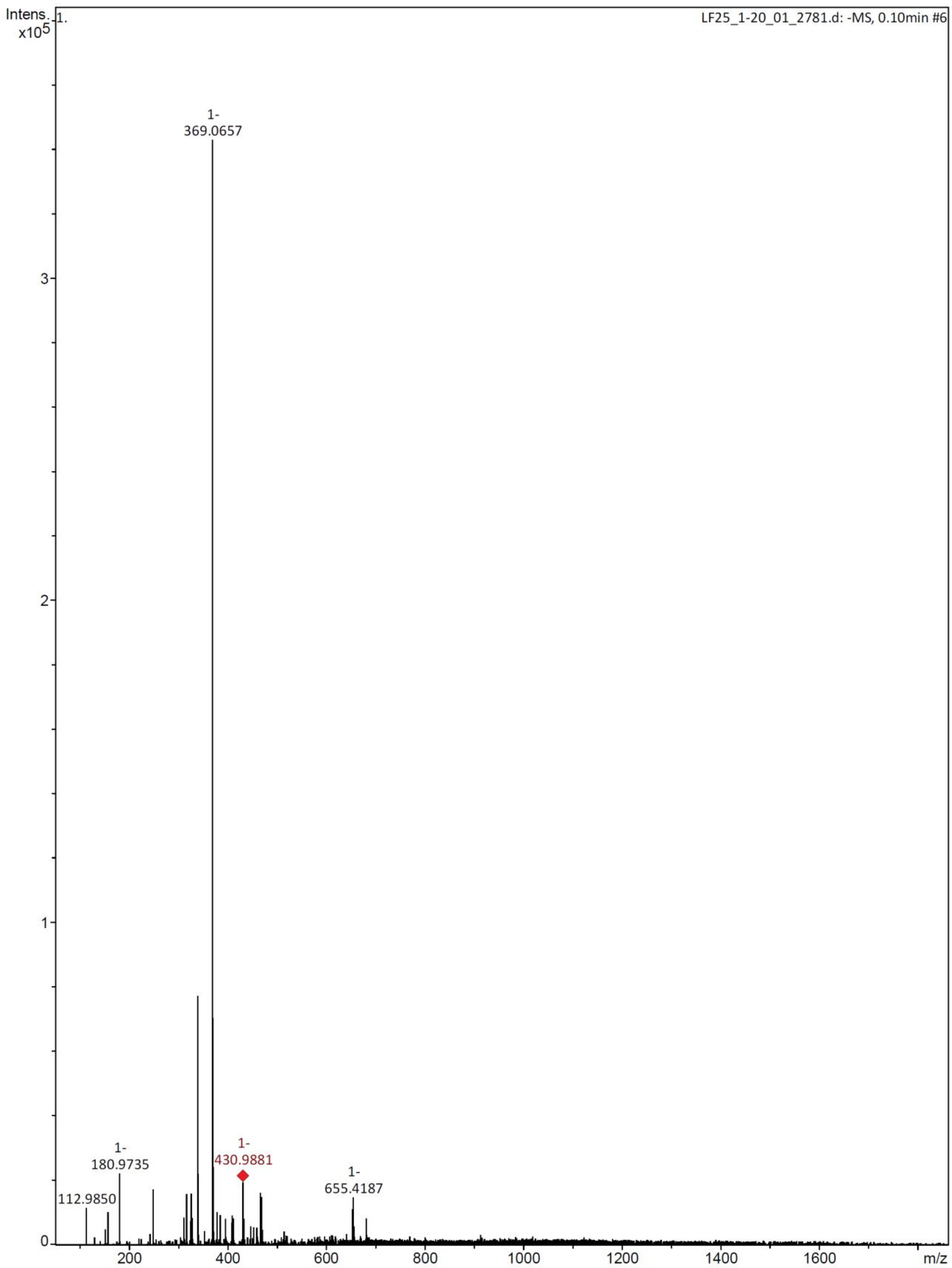

Figure S73 HRM spectrum of compound **25**

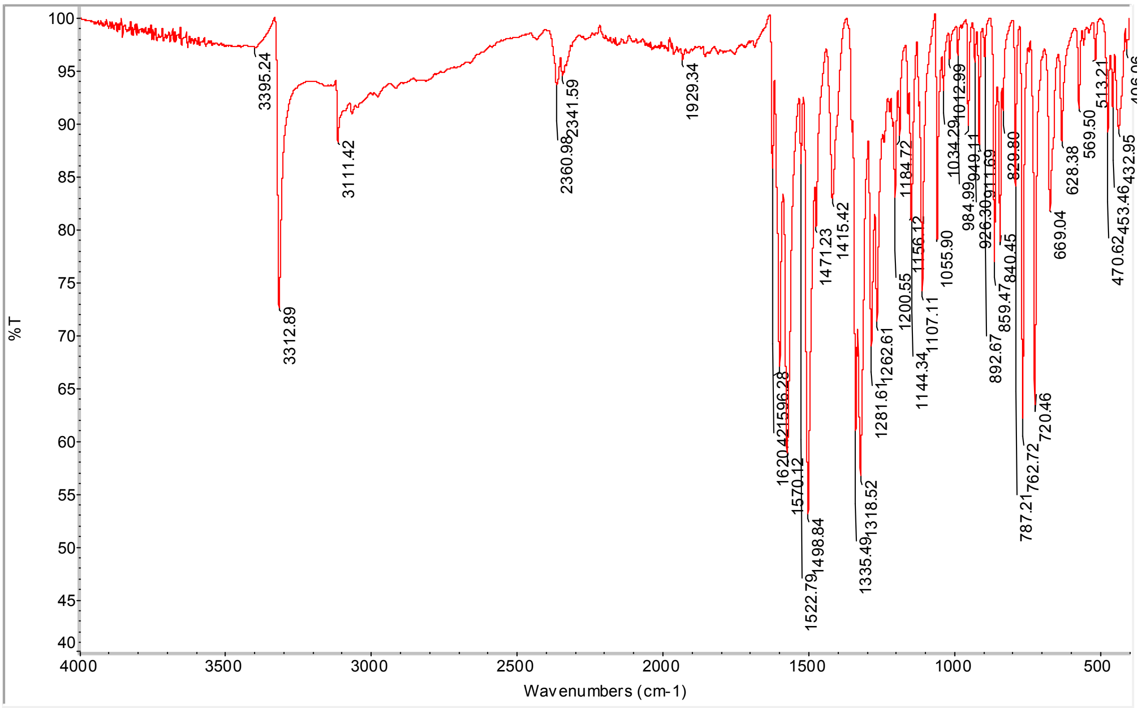

Figure S74 Infrared spectrum of compound **26**

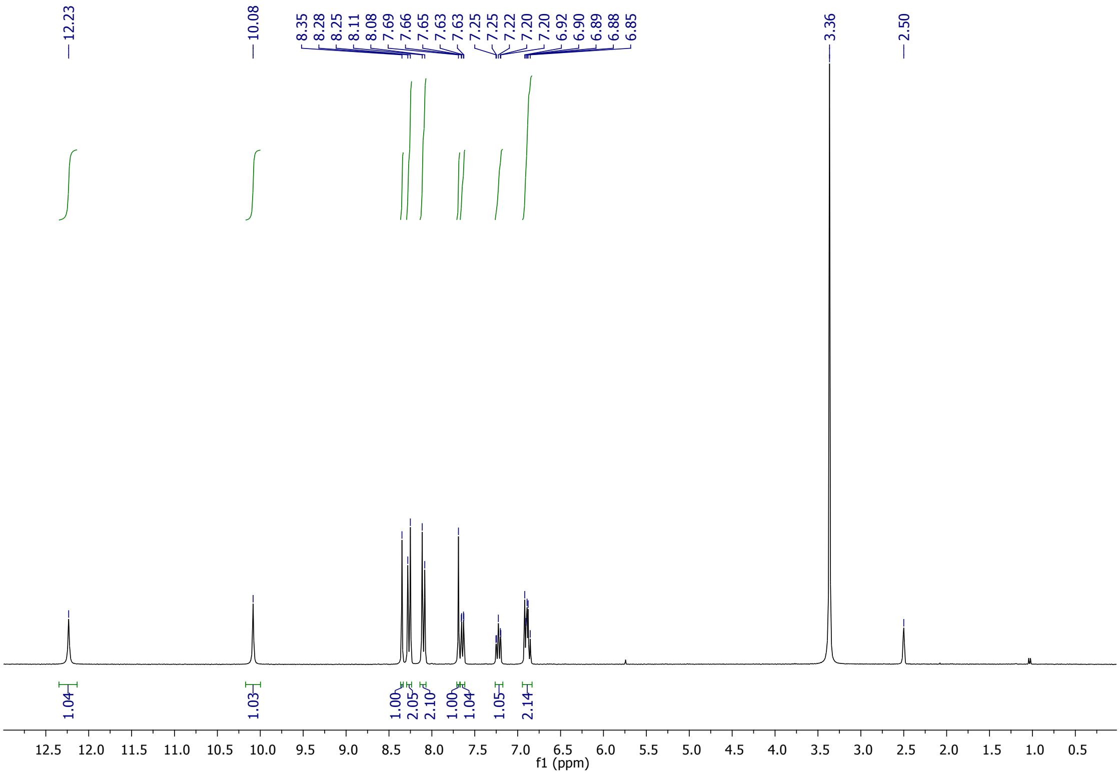

Figure S75 ^{1}H NMR spectrum of compound **26**

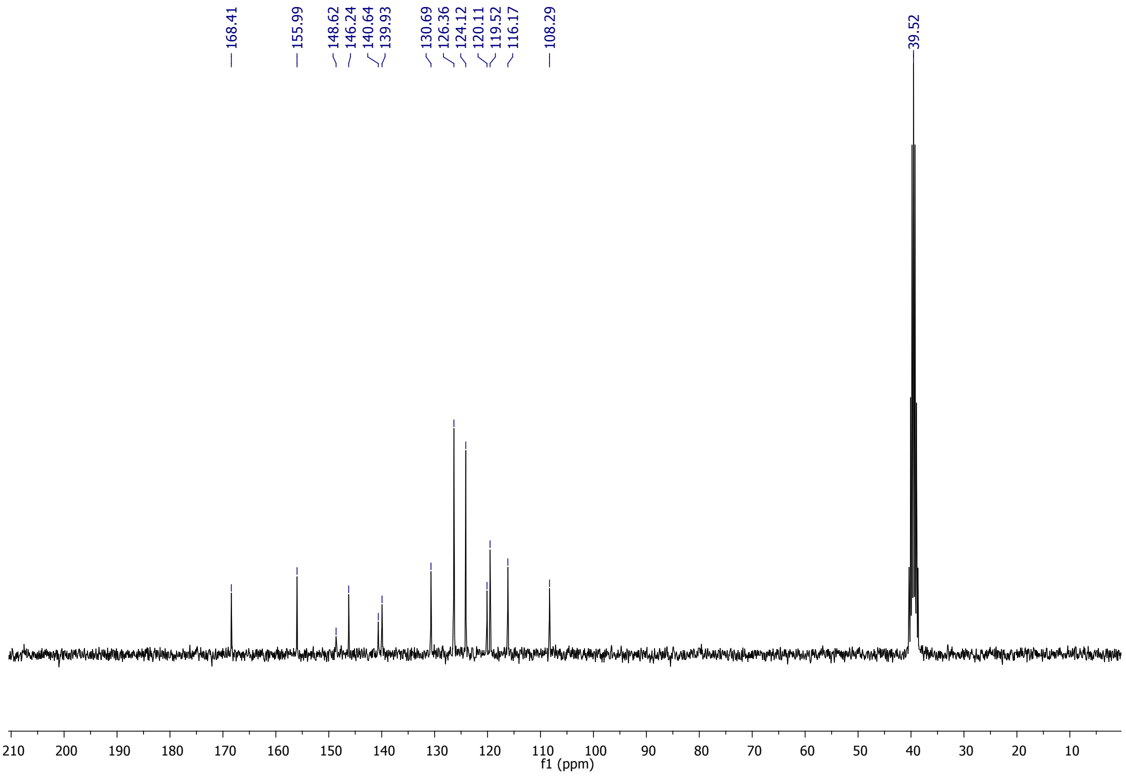

Figure S76 ^{13}C NMR spectrum of compound **26**

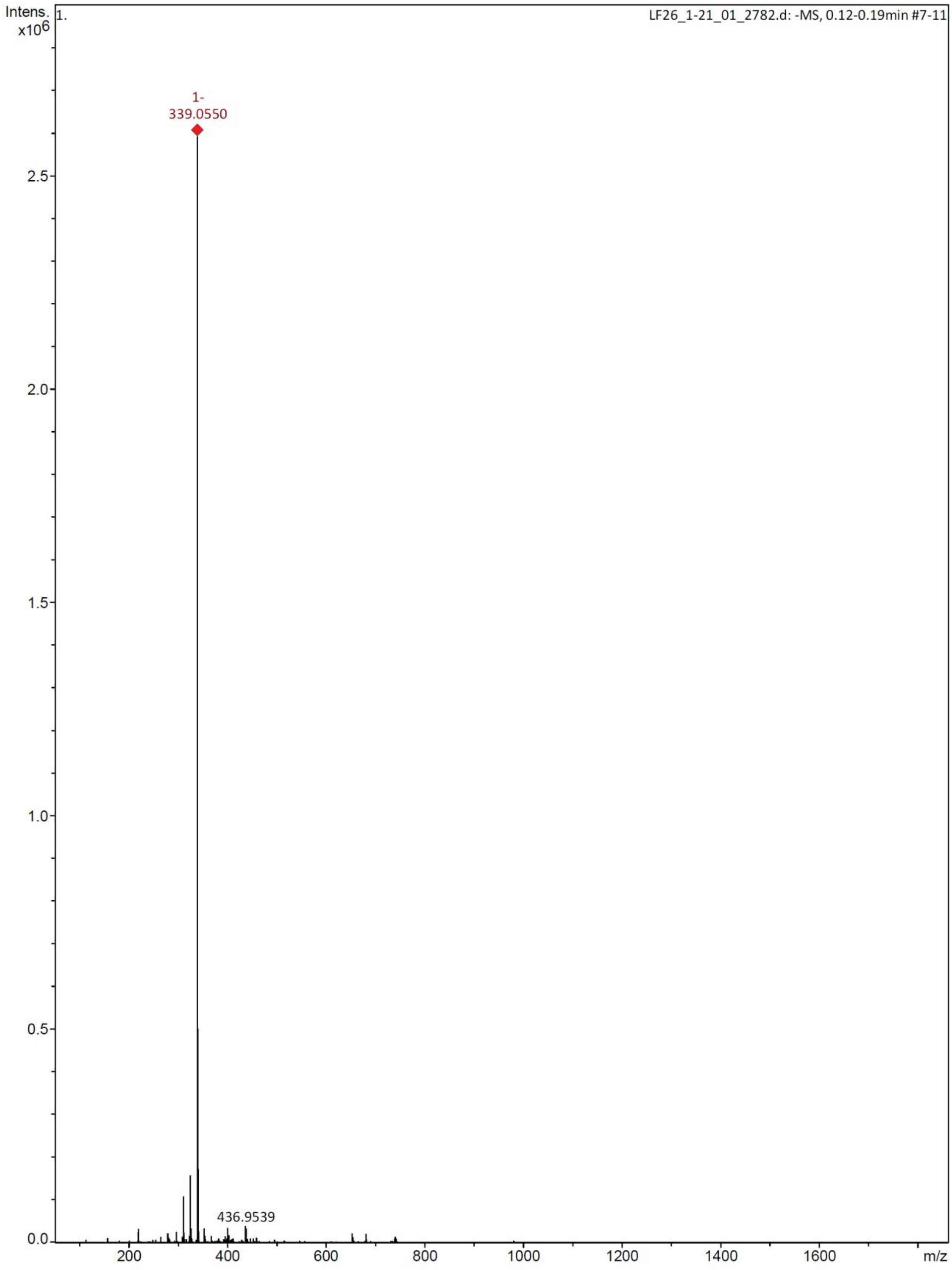

Figure S77 HRM spectrum of compound **26**
